# Regulation of Immune Checkpoints: From Molecular Mechanisms to Clinical Therapies

**DOI:** 10.1002/mco2.70771

**Published:** 2026-05-18

**Authors:** Qintao Ge, Liu Yu, Jiahe Lu, Aihetaimujiang Anwaier, Xiyue Xiao, Yonghao Chen, Dingwei Ye, Hailiang Zhang, Wenhao Xu, Wenbin Dai

**Affiliations:** ^1^ Department of Urology Shanghai Key Laboratory of Clinical Geriatric Medicine Huadong Hospital Fudan University Shanghai China; ^2^ Department of Urology, Fudan University Shanghai Cancer Center, Fudan University Shanghai China; ^3^ Department of Oncology Shanghai Medical College Fudan University Shanghai China; ^4^ Shanghai Genitourinary Cancer Institute Shanghai China; ^5^ West China Medical Center of Sichuan University Chengdu Sichuan China

**Keywords:** immune checkpoints, immune combination therapies, immune escape, immune resistance, immunotherapy, tumor microenvironment

## Abstract

Immune checkpoint inhibitors (ICIs) have transformed cancer therapy by targeting crucial coinhibitory pathways. Pathways involving PD‐1/PD‐L1 and CTLA‐4 are essential for immune homeostasis but are frequently exploited by tumors to evade immune surveillance. ICIs block these interactions, unleashing a potent antitumor immune response. However, broad clinical efficacy is limited by low response rates, immune‐related adverse events, and the pervasive challenge of primary or acquired resistance. This review summarizes the fundamental molecular mechanisms of key checkpoints (PD‐1/PD‐L1, CTLA‐4, TIM‐3) and highlights recent clinical progress in ICI monotherapies and combination strategies. We systematically explore multifaceted mechanisms of resistance, encompassing both tumor‐intrinsic and extrinsic factors. Furthermore, we outline novel therapeutic approaches being designed to overcome this resistance. Finally, we discuss promising predictive biomarkers and precision immuno‐oncology strategies. This review provides a critical framework for navigating the current landscape and offers a rationale to guide the future development of effective combination therapies to improve patient outcomes.

## Graphical abstract

Immune checkpoint inhibitors (ICIs) have revolutionized cancer treatment by unleashing antitumor immunity. This review comprehensively examines the molecular mechanisms underlying key immune checkpoints—including PD‐1/PD‐L1, CTLA‐4, and TIM‐3—and their clinical applications. We systematically evaluate ICI‐based therapeutic strategies, encompassing monotherapy and synergistic combinations with chemotherapy, radiotherapy, targeted therapy, cancer vaccines, cytokines, gut microbiota modulation, and metal ions that reshape the tumor microenvironment. We further discuss predictive biomarkers, immune‐related adverse events, and emerging technologies to advance personalized immunotherapy.

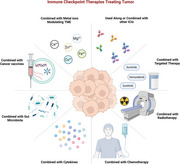

## 1 Introduction

Traditional cancer therapies, including surgery, radiotherapy, and chemotherapy, are often limited by a lack of precise molecular targeting and frequently fail to achieve complete eradication of disseminated disease. Consequently, immunotherapy has dramatically reshaped modern oncology, establishing itself as a fourth fundamental pillar of cancer treatment [1, 2]. Unlike conventional approaches that directly target tumor cells, immunotherapy activates the host's own antitumor immunity. This strategy offers distinct advantages, including high tumor specificity, the potential for durable, long‐term responses through immune memory formation, and generally lower toxicity, thereby reducing the risk of recurrence.

**FIGURE 1:**
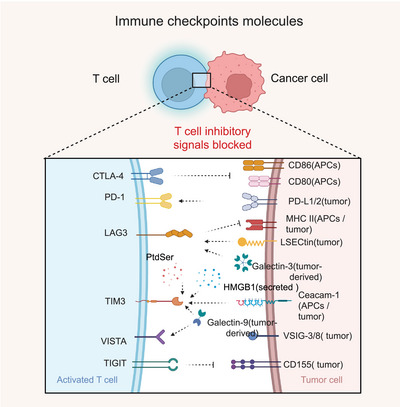
Immune checkpoint molecules mediating T cell suppression in the tumor microenvironment. This figure depicts the major inhibitory immune checkpoint pathways between activated T cells and tumor cells. Inhibitory receptors on T cells, including CTLA‐4, PD‐1, LAG‐3, TIM‐3, VISTA, and TIGIT, interact with their corresponding ligands expressed on tumor cells (e.g., PD‐L1/PD‐L2, LSECtin, galectin‐9, galectin‐3, VSIG‐3/8, CD155) or on antigen‐presenting cells (APCs) (e.g., CD80/CD86, MHC II, CEACAM‐1), as well as soluble or secreted ligands such as HMGB1 or phosphatidylserine (PtdSer). These ligand–receptor engagements deliver inhibitory signals that suppress T cell receptor (TCR) activation, reduce costimulatory signaling, and impair cytokine production, ultimately limiting T cell effector function and promoting tumor immune escape. Blocking these pathways with immune checkpoint inhibitors (ICIs) can reverse T cell dysfunction and restore antitumor cytotoxic activity (created in https://BioRender.com).

A pivotal breakthrough within this field is the development of immune checkpoint inhibitors (ICIs). Immune checkpoints are a network of regulatory molecules, comprising receptors on immune cells and their corresponding ligands, which transmit coinhibitory or costimulatory signals to maintain immune homeostasis (Figure 1). A major milestone in the exploration of immune checkpoints occurred in 1992, when Ishida et al. first identified the programmed cell death protein 1 (PD‐1) molecule in a mouse T cell hybridoma apoptosis model [3]. The subsequent translation of these fundamental discoveries into clinical practice marked a new era in oncology. This began with the United States Food and Drug Administration (US FDA) approval of ipilimumab (an anticytotoxic T‐lymphocyte‐associated antigen 4 [CTLA‐4] antibody) in 2011 for metastatic melanoma [4], followed by the approval of PD‐1 inhibitors nivolumab and pembrolizumab in 2014. Since these initial approvals, the application of ICIs has rapidly expanded to diverse cancers like renal cell carcinoma (RCC) and non‐small cell lung cancer (NSCLC) [5, 6] and significantly improved clinical outcomes even for low‐immunogenic tumors such as triple‐negative breast cancer (TNBC) [7].

The clinical impact of ICIs continues to grow, as evidenced by landmark trials. The KEYNOTE‐355 trial, for example, showed that adding pembrolizumab to chemotherapy significantly improved overall survival (OS) in TNBC patients (23.0 vs. 16.1 months) [8]. Subsequently, the 2021 KEYNOTE‐522 trial led to the US FDA approval of a pembrolizumab–chemotherapy combination for early‐stage TNBC based on a 14% absolute increase in pathological complete response rates, marking a major therapeutic advance [9]. However, despite transforming cancer treatment paradigms, significant clinical challenges persist. Response rates to ICI monotherapy in solid tumors remain modest, at approximately 20%, and many patients ultimately develop either primary or acquired resistance [10, 11]. This resistance is driven by a complex interplay of factors, including neoantigen depletion, impaired antigen presentation, T cell exclusion, dysregulated interferon signaling, metabolic abnormalities, and epigenetic changes. Furthermore, the predictive biomarker system for patient stratification remains imperfect, complicating the optimization of these powerful therapies.

To overcome these limitations, research is now focused on two key areas: developing reliable predictive biomarkers and, critically, investigating synergistic combination therapies. The rationale for combination strategies stems from the complex regulation of immune checkpoints themselves. Checkpoint expression is dynamically controlled at multiple levels, from oncogenic signaling pathways like the epidermal growth factor receptor (EGFR) and signal transducer and activator of transcription 3 (STAT3) pathways driving programmed cell death ligand protein 1 (PD‐L1) transcription [12] to posttranslational modifications (PTMs) like ubiquitination regulating protein stability [13, 14]. These intricate regulatory mechanisms contribute to monotherapy failure. Combination strategies aim to enhance efficacy through synergy; for example, chemoradiotherapy can induce immunogenic cell death (ICD) and release tumor antigens [15, 16]. Therefore, a comprehensive review that summarizes the regulatory mechanisms and clinical applications of immune checkpoint combination therapies is urgently needed.

We herein review various strategies related to immune checkpoints. First, we revisit the structures and functions of the most extensively studied immune checkpoints, including PD‐1, PD‐L1, CTLA‐4, and TIM‐3. Subsequently, we examine the primary combination therapeutic strategies involving immune checkpoint drugs currently applied in clinical practice, alongside the underlying regulatory mechanisms. Next, we summarize the opportunities for cancer combination therapy, all aimed at overcoming the immunosuppressive environment of tumor cells and maximizing the benefits of immunotherapy. Additionally, we outline the limitations of immunotherapy that targets immune checkpoints and discuss future prospects in this field. Overall, our goal is to summarize the clinical efficacy and molecular mechanisms of immune checkpoint combinations with other therapies, which may serve as potential methods to overcome the limitations of immune checkpoint drug therapy and address tumor immune escape.

## 2 ICIs: Structures and Regulation

**FIGURE 2:**
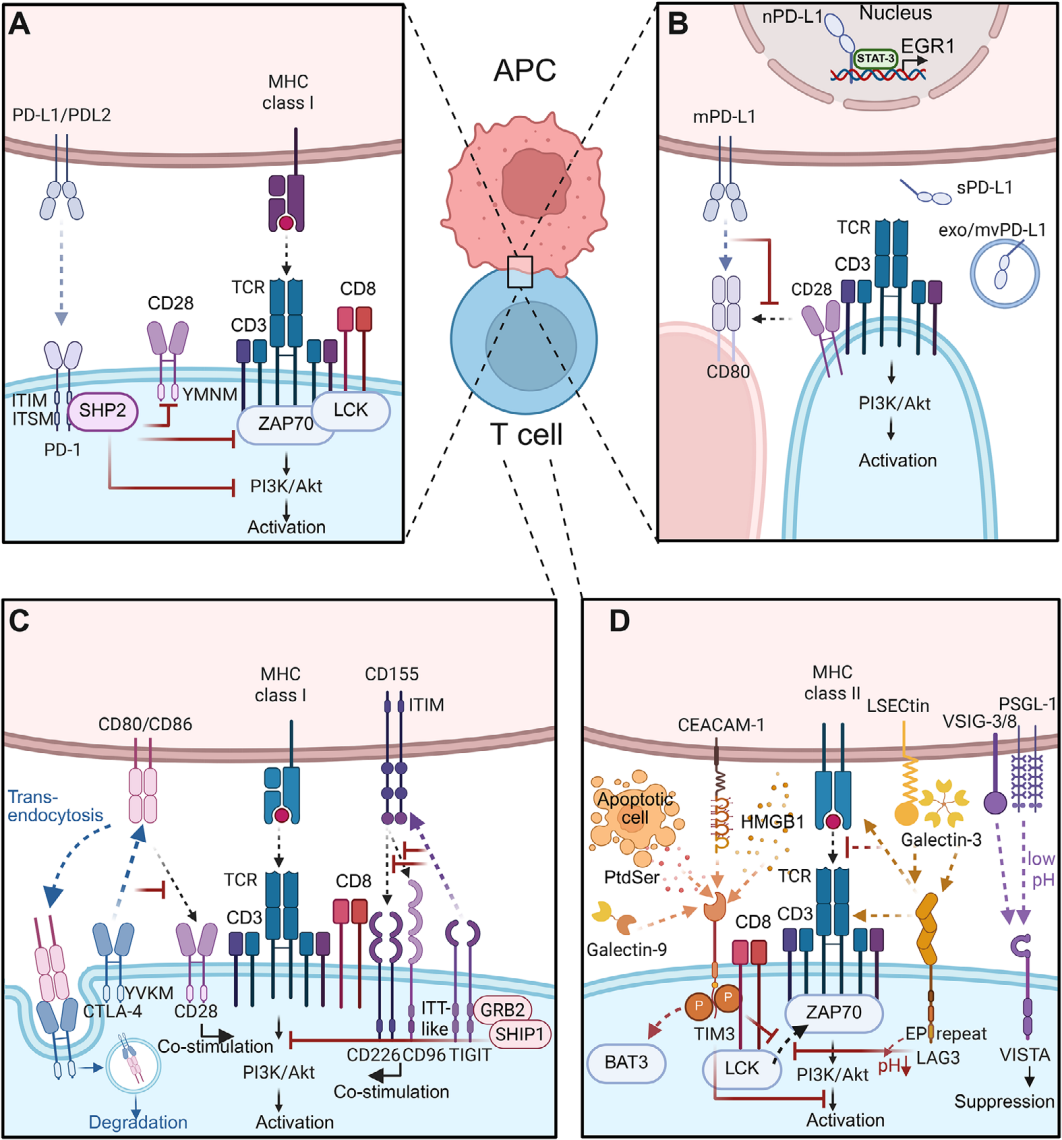
Illustrative diagram of the molecular mechanisms and regulatory pathways of major immune checkpoints. (A) PD‐1 signaling: upon engagement with PD‐L1/PD‐L2, PD‐1 ITIM/ITSM motifs are phosphorylated, recruiting and activating SHP2, which subsequently dephosphorylates CD28 and ZAP70 to suppress downstream PI3K–Akt signaling, leading to impaired T cell activation. (B) PD‐L1 regulation and signaling: tumor cells express multiple PD‐L1 isoforms, including membrane‐bound PD‐L1 (mPD‐L1), cytoplasmic PD‐L1, nuclear PD‐L1 (nPD‐L1), soluble PD‐L1 (sPD‐L1), and exosomal/microvesicle PD‐L1. mPD‐L1 modulates T cell responses via CD80 cis/trans interactions, whereas nPD‐L1 promotes transcriptional activation of oncogenic programs such as STAT3‐mediated EGR1 expression. (C) CTLA‐4 and TIGIT pathways: CTLA‐4 outcompetes CD28 for CD80/CD86 binding and mediates trans‐endocytosis of B7 ligands, thereby inhibiting early T cell priming. TIGIT competes with CD226 for CD155 binding, suppressing costimulatory signaling and dampening T cell activation. (D) TIM‐3, LAG‐3, and VISTA pathways: TIM‐3 interacts with multiple ligands, including galectin‐9, phosphatidylserine (PtdSer) on apoptotic cells, HMGB1, and CEACAM‐1. Phosphorylation of its cytoplasmic tail triggers BAT3 dissociation and subsequent inhibitory signaling. LAG‐3 binds to MHC class II molecules or, as recently elucidated, directly associates with the TCR–CD3 complex, causing pH drop at the immune synapse, which drives the dissociation of the coreceptor Lck from CD4 or CD8, thereby disrupting proximal TCR signaling through ZAP70 and downstream PI3K–Akt activation. VISTA functions as a pH‐dependent immune checkpoint, exhibiting enhanced ligand binding under acidic conditions characteristic of the tumor microenvironment, thereby mediating context‐dependent immune suppression (created in https://BioRender.com).

Immune checkpoints are the central regulators of immune homeostasis and the primary targets of ICI therapy. Understanding their fundamental biology is essential for optimizing treatment and overcoming resistance. This section will systematically review the molecular structures, signaling pathways, and complex regulatory mechanisms of the most critical immune checkpoints. We will detail the biology of the extensively studied PD‐1/PD‐L1 axis and the CTLA‐4 pathway, followed by an analysis of TIM‐3 and other emerging novel checkpoints that represent the next wave of therapeutic targets (Figure 2).

### 2.1 Programmed Cell Death Protein 1

PD‐1 (also known as PDCD1 and CD279) is the most extensively studied immune checkpoint, initially identified by Ishida et al. in apoptotic murine T cell tumors [3]. As an inhibitory receptor of the CD28/CTLA‐4 family, PD‐1 is broadly expressed on activated immune cells (T cells, B cells, monocytes) and downregulates immune activity via PD‐L1/PD‐L2 interactions. Structurally, this 288‐residue Type I membrane protein comprises an extracellular immunoglobulin variable (IgV) domain, a transmembrane region, and a cytoplasmic tail bearing both immunoreceptor tyrosine‐based inhibitory motif (ITIM) and immunoreceptor tyrosine‐based switch motif (ITSM) critical for its inhibitory function [17, 18]. Phosphorylation cycling of ITIM/ITSM motifs forms the structural platform for PD‐1‐mediated inhibition, yet the receptor's functional unit remains debated.

Current models diverge on whether PD‐1 operates as a monomer or requires dimerization for optimal inhibitory signaling. Early studies, based on 2.0 Å crystal structures of the isolated extracellular domain [18], suggested PD‐1 exists strictly as a monomer in solution. This stands in contrast to other CD28 family members like CTLA‐4, which form homodimers linked by extracellular disulfide bonds. However, this discrepancy may stem from the absence of a native membrane context in earlier crystallographic work. Recent biochemical studies by Philips et al. demonstrate that PD‐1 does form dimers, mediated specifically by interactions within the transmembrane domain [19]. Thus, the physiological form of PD‐1 is likely a dynamic, transmembrane‐driven dimer rather than a static monomer. This structural organization is functionally relevant: the PD‐1 signaling cascade requires Src homology region 2 domain‐containing phosphatase‐2 (SHP2) recruitment via phosphorylated ITSM and ITIM motifs. Transmembrane‐mediated dimerization likely aligns these motifs across adjacent receptors, creating a scaffold that facilitates the relief of SHP2 autoinhibition. Elucidating this dynamic is therefore critical for rational drug design. If dimerization is a prerequisite for signaling, standard bivalent antibodies could inadvertently cross‐link PD‐1, functioning as agonists. In this scenario, monovalent or noncross‐linking antibodies would be necessary to ensure effective antagonistic blockade.

Upon ligand binding and structural alignment, the core inhibitory signaling cascade is initiated. Activated SHP2 phosphatase dephosphorylates key signaling molecules, exhibiting a marked preference for the costimulatory receptor CD28 over T cell receptor (TCR) components such as zeta‐chain‐associated protein kinase 70 (ZAP70). This preference results in the blunting of CD28‐mediated costimulation and the subsequent inhibition of downstream cascades, including the PI3K/Akt pathway [17, 20], which suppresses IL‐2 secretion [17]. Furthermore, sustained suppression of PI3K/Akt signaling drives effector T cells toward apoptosis and exhaustion [21], while also restricting their migration across lymphatic endothelial cells [22].

Under normal physiological conditions, PD‐1 and PD‐L1 molecules play a crucial role in maintaining immune homeostasis. However, in the context of tumors, the PD‐1/PD‐L1 inhibitory axis plays a pivotal role in facilitating tumor development and progression through immune suppression. The overexpression of immune checkpoint molecules is one of the critical mechanisms through which tumors achieve immune evasion. Tumors can escape immune detection by upregulating PD‐L1 expression, which suppresses immune activity upon binding to PD‐1 on effector T cells, ultimately inducing effector T cell apoptosis [23]. Beyond its effects on effector T cells, the PD‐1/PD‐L1 axis modulates the activity of diverse immune cell populations in the tumor microenvironment (TME). By attenuating the PI3K/Akt/mTOR cascade, PD‐1 signaling creates a metabolic environment that favors the differentiation and maintenance of immunosuppressive regulatory T cells (Tregs) [24, 25]. Additionally, PD‐1 expression on tumor‐associated macrophages (TAMs) suppresses their phagocytic activity [26].

The ability of PD‐1 signaling to impair effector T cells while promoting Tregs stems from the differential reliance of these subsets on the PI3K/Akt/mTOR axis and their distinct metabolic requirements. Effector T cells require robust Akt/mTOR signaling to drive glycolysis, proliferation, and cytotoxicity [20]. PD‐1‐mediated recruitment of SHP2, which dephosphorylates key components of the PI3K/Akt cascade, restricts these metabolic processes, promoting exhaustion and apoptosis. In contrast, Treg development and stability, governed by the transcription factor Foxp3, are compromised by high Akt/mTOR activity, which downregulates Foxp3 expression [27]. By attenuating PI3K/Akt/mTOR signaling, PD‐1 relieves this repression. Furthermore, the inhibition of glycolysis shifts metabolism toward fatty acid oxidation (FAO), a state that supports Treg survival [28, 29]. Thus, the same PD‐1 signaling events that drive metabolic exhaustion in effector T cells simultaneously create a niche favorable for Treg differentiation and stability.

Given its potent capacity to reprogram the immune microenvironment and drive these metabolic shifts, the expression of PD‐1, encoded by the PDCD1 gene, is dynamically controlled at the transcriptional, epigenetic, and posttranslational levels. Transcription of PDCD1 is activated by various stimuli, including TCR and cytokine signaling [30, 31]. Upon T cell activation, the transcription factor nuclear factor of activated T cells 1 is induced and represents a critical first step for PD‐1 expression [32]. In parallel, cytokines such as IL‐2, IL‐6, IL‐12, and IFN‐α also drive PDCD1 transcription via STAT proteins [33, 34]. This activation is balanced by repressors. For instance, B lymphocyte‐induced maturation protein‐1 represses PD‐1 expression during the late phase of acute infections, whereas T‐box expressed in T cells (T‐bet) sustains effector T cell responses in chronic infections by repressing PD‐1 [35, 36]. Epigenetic modifications are also critical for determining T cell fate. In naïve T cells, the PDCD1 promoter regions are hypermethylated, keeping expression silenced. However, during chronic infection, these regions become demethylated, leading to the sustained, high‐level PD‐1 expression characteristic of effector T cell exhaustion [32, 37]. Finally, posttranslational mechanisms control the stability and localization of the PD‐1 protein itself. PD‐1 protein levels are regulated by the ubiquitin–proteasome system; the E3 ligase F‐box only protein 38 mediates polyubiquitination at K233, targeting PD‐1 for degradation [38]. This degradation is inhibited by core fucosylation (a type of glycosylation) mediated by α‐1,6‐fucosyltransferase, which is necessary to stabilize PD‐1 expression on the cell surface [39, 40]. Additionally, the exhaustion‐associated transcription factor thymocyte selection‐associated HMG BOX (TOX) plays a dual role by binding to PD‐1 in the cytoplasm, which reduces its degradation and promotes its translocation back to the cell surface [41].

### 2.2 Programmed Cell Death Ligand Protein 1

PD‐L1 (encoded by CD274) belongs to the immunoglobulin superfamily and is classified as a Type I transmembrane glycoprotein. The full‐length PD‐L1 is composed of three regions: a transmembrane region, a cytoplasmic tail region, and an extracellular region that includes a signal peptide, an Ig‐like V‐type domain, and two Ig‐like C‐type domains [42, 43]. PD‐L1 demonstrates a multifaceted subcellular distribution pattern, with localization observed at the plasma membrane, cytoplasmic compartments, nuclear envelope, and extracellular milieu through active secretion [44].

Early research and drug development primarily focused on membrane‐bound PD‐L1 (mPD‐L1), targeting its direct ligation to PD‐1. Beyond this classical blockade, mPD‐L1 also modulates immunity through a bidirectional interaction with CD80, with the biological consequence depending on its spatial orientation [45]. Intracellular cis‐interaction acts as a sequestration mechanism, neutralizing both mPD‐L1 and CD80 to preclude their engagement with PD‐1 and CTLA‐4, thereby unleashing T cell activity [46, 47]. By contrast, intercellular trans‐interaction preserves immunosuppressive function by inhibiting CD8^+^ T cell expansion and compromising antitumor responses [48].

The biological activity of PD‐L1 is not strictly confined to the plasma membrane. Emerging evidence points to noncanonical PD‐L1 isoforms, including cytoplasmic, nuclear, and secreted variants, that operate independently of the immune synapse [49, 50]. These subtypes exhibit distinct structural characteristics and trafficking pathways. Unlike mPD‐L1, which possesses a complete transmembrane domain and undergoes HIP1R‐mediated internalization, cytoplasmic PD‐L1 (cPD‐L1) often lacks membrane‐anchoring domains and is recycled via transport protein particle complex subunit 4‐dependent mechanisms [51]. Consequently, this isoform is predominantly retained within the cytoplasm, where it promotes tumor proliferation and drug resistance through nonimmune mechanisms [50, 51]. Furthermore, nuclear PD‐L1 (nPD‐L1) is formed by the shuttling of cPD‐L1 from the cytoplasm to the nucleus, as observed in circulating tumor cells collected from patients with colorectal cancer and prostate cancer, and the expression of nPD‐L1 is significantly associated with shorter survival [52]. Mechanistically, PD‐L1 can translocate to the nucleus with the assistance of Karyopherin‐β1, thereby activating the growth arrest‐specific 6/myeloid–epithelial–reproductive tyrosine kinase signaling pathway and promoting the proliferation of NSCLC cells [53]. Recent studies have demonstrated that nPD‐L1 also serves as an endogenous accelerator for cancer angiogenesis, as it promotes the binding of phosphorylated STAT3 (p‐STAT3) to the early growth response‐1 (EGR1) promoter, leading to the activation of EGR1‐mediated angiogenesis [54].

In addition to its presence on the cell surface, PD‐L1 can be released into the extracellular environment, giving rise to several forms, such as soluble PD‐L1 (sPD‐L1), exosomal PD‐L1 (exoPD‐L1), and microvesicle PD‐L1 (mvPD‐L1). Notably, exoPD‐L1 has been identified as a potential biomarker for assessing tumor progression and predicting responses to ICIs. A study by Theodoraki et al. found that the levels of PD‐L1 found in exosomes derived from tumors in patients with head and neck squamous cell carcinomas (HNSCC) exhibited a positive correlation with disease activity and lymph node involvement [55]. Mechanistically, tumor cells can secrete exoPD‐L1 from the cytoplasm to distant tissues via a “secretion–adhesion” mechanism, where it binds to PD‐1 on the surface of T cells, thereby inducing a systemic immunosuppressive state [56, 57, 58]. Recent studies have demonstrated that PD‐L1 carried by tumor‐derived extracellular vesicles can induce DNA damage and cellular senescence by activating cAMP‐response element binding protein and STAT signaling [59].

Transcriptional control of the CD274 gene (encoding PD‐L1) is broadly categorized into two mechanisms. The first is adaptive immune resistance, where inflammatory cytokines, most notably IFN‐γ secreted by effector T cells, activate the janus kinase (JAK)/STAT signaling cascade to drive PD‐L1 expression [60]. This forms a negative feedback loop in response to an active antitumor immune attack. Second, PTMs are critical for controlling PD‐L1 protein stability. N‐linked glycosylation of PD‐L1 is essential, as it shields the protein from ubiquitination and subsequent proteasomal degradation [61, 62]. This glycosylation physically hinders the access of E3 ubiquitin ligases, such as speckle‐type POZ protein and beta‐transducin repeat‐containing protein, which would otherwise mark PD‐L1 for destruction [63, 64, 65]. The interplay between ubiquitination and deubiquitination thus serves as a rapid mechanism to modulate cell surface PD‐L1 levels.

### 2.3 Cytotoxic T‐Lymphocyte‐Associated Antigen 4

CTLA‐4 is an immune checkpoint molecule primarily expressed by T cells. CTLA‐4 is a 25 kDa transmembrane receptor whose structure is similar to that of CD28 [66]. Unlike CD28, which is predominantly expressed on the cell surface, CTLA‐4 is localized within the trans Golgi network (TGN), endosomes, secretory granules, and lysosomal vesicles [67]. Emerging evidence indicates that CTLA‐4 is expressed not only by T lymphocytes but also across diverse neoplastic cell populations, encompassing both solid tumors and hematopoietic malignancies [68, 69, 70, 71]. CTLA‐4 serves as a critical regulator of immune tolerance by competitively binding to CD80/CD86 ligands. This interaction blocks the CD28‐mediated costimulatory signals required for T cell proliferation during early immune activation [72].

#### 2.3.1 CTLA‐4 as a Competitive Inhibitor of CD28 Costimulation

T cell activation requires coordinated dual signaling. The primary signal occurs through TCR engagement with peptide major histocompatibility complex (MHC) complexes, while the secondary signal involves CD28‐mediated costimulation following interaction with B7 family molecules (CD80/CD86) expressed on antigen‐presenting cells (APCs). Notably, although CTLA‐4 competes with CD28 for binding to CD80 and CD86, it demonstrates superior binding affinity for these ligands [73, 74]. Consequently, the primary function of CTLA‐4 is to competitively bind to CD80/CD86, thereby blocking the stimulatory signal for T cell proliferation provided by CD28 during the initial phase [75].

#### 2.3.2 The Role of CTLA‐4 in Tregs

Beyond competitive binding, CTLA‐4 is fundamentally implicated in the suppressive machinery of Tregs [76]. High expression of CTLA‐4 on Tregs enables the physical removal of B7 molecules (CD80/CD86) from APCs via transendocytosis, a process driven by the molecule's high affinity and specific intracellular trafficking [77, 78, 79]. CTLA‐4 also exerts an extracellular effect by promoting the activity of IDO in dendritic cells (DCs) [80]. IDO serves as a critical immunomodulatory enzyme that facilitates tumor immune evasion through its catalytic conversion of tryptophan into kynurenine. Increased IDO activity results in a significant reduction in tryptophan levels and a substantial accumulation of kynurenine in the local microenvironment. In the absence of tryptophan, T cells experience an arrest in their cell cycle at the mid‐G1 phase, and this arrest cannot be reversed by the subsequent restoration of tryptophan [81]. The kynurenine‐mediated apoptotic pathway exhibits selective cytotoxicity toward both thymocytes and antigen‐experienced CD4^+^T effector cells through IDO‐dependent mechanisms. This process involves mitochondrial cytochrome *c* release and caspase‐8 activation, constituting a canonical intrinsic apoptosis pathway [82, 83, 84].

#### 2.3.3 Additional Regulatory Functions and Trafficking Dynamics

CTLA‐4 exists in both membrane‐bound and soluble isoforms. The soluble form, primarily produced by Tregs, inhibits T cell responses by binding to B7 molecules, thereby suppressing proliferation and cytokine secretion [85, 86]. Blocking sCTLA‐4 enhances antitumor immunity, underscoring its role in extrinsic immune regulation. CTLA‐4 is essential for maintaining the quiescent state of memory T cells. Mechanistically, this quiescence is orchestrated by Tregs, which inhibit the differentiation and proliferation pathways of effector T cells via CTLA‐4. When Treg cells are lacking, memory T cells initiate genome‐wide transcriptional programs typical of effector T cells, leading to a loss of their quiescence [87]. In TME, Zappasodi et al. demonstrated that CTLA‐4 blockade enhances T cell metabolic fitness and destabilizes Tregs in glucose‐deficient tumors, thereby promoting antitumor immunity [88]. This effect relies on Treg glycolysis and CD28 signaling, indicating that the combination of CTLA‐4 inhibitors with tumor glycolysis inhibitors may enhance immunotherapy outcomes by alleviating glucose competition within the TME. Additionally, CTLA‐4 can indirectly regulate B cell responses. Sage et al. [89] discovered that CTLA‐4 mediates humoral immunity through its multifaceted roles in T follicular helper (Tfh), T follicular regulatory, and Tregs.

A unique feature of CTLA‐4 is that its inhibitory function is regulated less by transcriptional induction and more by its dynamic protein trafficking [90]. On conventional T cells, CTLA‐4 is synthesized but rapidly internalized and sequestered in intracellular compartments, including the TGN [91] and recycling endosomes [92]. Following strong T cell activation, this internalization pathway is transiently inhibited, leading to a rapid accumulation of CTLA‐4 on the plasma membrane [93]. This mechanism allows CTLA‐4 to be deployed precisely when T cell activation peaks, providing a potent negative feedback signal. In contrast, in Tregs, where CTLA‐4 is critical for their suppressive function, this trafficking is modulated differently to allow for more sustained surface expression [94, 95].

### 2.4 T Cell Immunoglobulin and Mucin Domain‐Containing Protein 3

TIM‐3 is a member of the TIM receptor family, which includes three members in humans: TIM‐1, TIM‐3, and TIM‐4. Among these, TIM‐3 has attracted significant attention due to its strong association with T cell exhaustion and dysfunction in cancer [96]. TIM‐3 is predominantly expressed on interferon‐γ‐producing CD4^+^T helper 1 (Th1) cells and CD8^+^cytotoxic T lymphocyte 1 cells [97]. Its family members, TIM‐1 and TIM‐2, are predominantly expressed on T helper Type 2 cells [98]. TIM‐3 demonstrates broad cellular distribution across both immune and nonimmune compartments. In addition to conventional T lymphocytes, TIM‐3 expression has been documented on natural killer cells (NK cells), macrophages, DCs, myeloid‐derived suppressor cells (MDSCs), mast cells, and malignant cells originating from diverse tissue types [99]. Recent studies have also demonstrated that B cells can express TIM‐3 [100, 101]. The human TIM‐3 gene is mapped to chromosomal region 5q33.2 and encodes a Type I membrane‐anchored glycoprotein. Its extracellular tail features an N‐terminal IgV‐like domain, which, along with the mucin domain, constitutes the extracellular domain of TIM‐3. This continues with a transbilayer domain and a C‐terminal cytoplasmic region [102, 103]. In contrast to canonical inhibitory checkpoints such as PD‐1 and CTLA‐4, TIM‐3 exhibits a distinct structural feature—the lack of conserved domains responsible for phosphatase recruitment that mediate immunosuppressive signaling [104]. The intracellular domain of TIM‐3 harbors multiple conserved tyrosine residues (including Y256 and Y263) within functional signaling motifs, which have been implicated as critical mediators of its immunoregulatory activity [105]. To date, four ligands of TIM‐3 have been identified: galectin‐9 (GAL‐9), high mobility group box 1 (HMGB1) [106], carcinoembryonic antigen cell adhesion molecule 1 (CEACAM‐1) [107], and phosphatidylserine [108]. GAL‐9 is the first identified and most extensively studied ligand of TIM‐3. It is widely expressed in tumor cells and is associated with tumor immune evasion [109, 110, 111, 112]. Interestingly, GAL‐9 also functions as a ligand for PD‐1, promoting the persistence of PD‐1^+^TIM‐3^+^ T cells by binding to PD‐1, while attenuating GAL‐9/TIM‐3‐induced cell death [113]. TIM‐3 is primarily regarded as a negative regulator of the immune function of CD8^+^T cells. Under physiological conditions, the cytoplasmic domain of TIM‐3 in CD8^+^T cells associates with HLA‐B‐associated transcript 3 (BAT3), suppressing TIM‐3‐mediated signaling and promoting T cell activation. Upon engagement with GAL‐9, TIM‐3 undergoes phosphorylation at tyrosine residues Y256 and Y263, leading to BAT3 dissociation. This event facilitates the transduction of inhibitory signals through TIM‐3 [105]. Emerging evidence indicates that HMGB1 interacts with TIM‐3 expressed on CD4^+^T cells, resulting in sustained suppression of nuclear factor kappa‐light‐chain‐enhancer of activated B cells (NF‐κB) signaling in TIM‐3^+^CD4^+^ T cell populations. This regulatory mechanism serves to constrain excessive T cell activation [114]. Beyond its role in T cell regulation, TIM‐3 significantly modulates myeloid cell activity. In macrophages, TIM‐3 serves as a key inhibitory receptor that suppresses their polarization into proinflammatory subsets. This mechanism involves TIM‐3 binding to STAT1 at Y256/Y263, which enhances suppressor of cytokine signaling 1 activity and promotes M2‐like macrophage polarization [115, 116]. Jiang et al. demonstrated that in dextran sulfate sodium‐induced murine colitis models, TIM‐3 inhibits the polarization of proinflammatory M1 macrophages, whereas its deletion or inhibition enhances the M1 response [117]. In conventional DCs, the regulation of extracellular DNA endocytosis and activation of the cGAS–STING pathway is governed by TIM‐3, which restricts chemokine (C–X–C motif) ligand 9 (CXCL9) production and antitumor immunity. Inhibition of TIM‐3 boosts DNA absorption and enhances the immune response of DCs [118]. As previously mentioned, TIM‐3 is widely expressed in various tumor tissues, including colon cancer, NSCLC, myeloid leukemia [119], hepatocellular carcinoma (HCC) [120], and melanoma [121]. Within the TME, TIM‐3 is predominantly localized on the surfaces of antigen‐specific CD8^+^ T cells, CD4^+^ T cells, and NK cells [122]. Similar to PD‐1 and CTLA‐4, TIM‐3 plays a significant role in tumor immune escape. In TNBC, anti‐TIM‐3 antibodies can enhance the response to paclitaxel chemotherapy through a mechanism dependent on CD8^+^T cells and granzyme B [123]. Fucikova et al. [124] discovered that PD‐1^+^TIM‐3^+^CD8^+^ T cells in high‐grade serous carcinoma are significantly associated with poor tumor prognosis, while exhibiting characteristics of T cell exhaustion. In NSCLC, high TIM‐3 expression in T cells correlates with the expression of proapoptotic markers and patient survival rates [125]. The underlying mechanism involves TIM‐3‐mediated signal transduction following engagement with tumor‐expressed ligands such as Gal‐9 and CEACAM‐1. These interactions promote T cell exhaustion and functional impairment, thereby supporting tumor cell survival and self‐renewal. Consequently, TIM‐3 blockade has emerged as a promising immunotherapeutic approach currently under investigation in multiple clinical trials [126].

The regulation of TIM‐3 (encoded by HAVCR2 gene) expression is intrinsically tied to T cell differentiation status and is a key marker of exhaustion [127]. In naïve T cells, the HAVCR2 gene is silenced. Upon activation and differentiation into Th1 effector cells, the master transcription factor T‐bet binds to the HAVCR2 promoter to initiate TIM‐3 expression [128].

### 2.5 Novel Immune Checkpoints

*Inhibitory immune checkpoints (TIGIT, VISTA, LAG‐3)*: These checkpoints play crucial roles in immune suppression within the TME. T cell immunoreceptor with Ig and ITIM domains (TIGIT), a member of the immunoglobulin superfamily, exerts a dual immunosuppressive effect [129]. Extracellularly, it competitively binds to the poliovirus receptor CD155 with higher affinity than the costimulatory receptor CD226, thereby abrogating positive costimulation [130]. Intracellularly, upon ligand binding, the phosphorylation of its immunoreceptor tail tyrosine‐like motif recruits the adapter Grb2, which in turn recruits SH2‐containing inositol phosphatase 1 [131]. This phosphatase attenuates the PI3K/mitogen‐activated protein kinase (MAPK) signaling cascades essential for NK and T cell activation and cytotoxicity.

V‐domain Ig suppressor of T cell activation (VISTA) operates through a distinct mechanism. Its structure confers pH‐dependent binding properties, and its predominant expression on myeloid cells allows it to suppress T cell responses through both receptor‐ and ligand‐like modalities [132].

Lymphocyte‐activation gene 3 (LAG‐3), meanwhile, has undergone a recent reinterpretation. While its interaction with MHC‐II is well‐established, Guy et al. demonstrated that LAG‐3 can function independently of this ligand by associating directly with the TCR‐CD3 complex [133]. Mechanistically, the acidic cytoplasmic tail of LAG‐3 lowers the local pH at the immune synapse, which drives the dissociation of the Src family kinase Lck from CD4 or CD8 coreceptors. This dissociation enforces dominant suppression of proximal TCR signaling and limits T cell expansion.

*Stimulatory immune checkpoints (ICOS, 4‐1BB, OX40, CD40L)*: These costimulatory molecules enhance antitumor immunity through distinct activation mechanisms. Inducible T cell costimulator (ICOS) promotes T cell proliferation and effector functions upon binding ICOS‐L on APCs, with its modulation showing potential to counteract Treg‐mediated suppression [134]. 4‐1BB (CD137) signaling activates NF‐κB and MAPK pathways to augment T cell survival and cytotoxic activity, while OX40(CD134)–OX40L interactions promote memory T cell formation and suppress Treg differentiation through tumor necrosis factor receptor‐associated factors‐mediated NF‐κB activation [135, 136, 137, 138]. CD40–CD40L engagement bridges adaptive and innate immunity by enhancing APC maturation and cytokine production, critically influencing T cell‐dependent B cell responses [139].

## 3 Expanding the Clinical Landscape of Immune Checkpoint Therapies

The translation of basic research on immune checkpoints into clinical practice has generated remarkable survival benefits across numerous malignancies. This section reviews the clinical evolution of immune checkpoint‐based treatments. We will first discuss the foundational success of ICI monotherapy, which established this approach as a pillar of oncology. Subsequently, we will focus on the primary driver of modern immuno‐oncology: synergistic combination strategies. This includes a comprehensive summary of dual‐ICIs blockade, as well as combinations with chemotherapy, targeted therapy, oncolytic viruses (OVs), and radiotherapy. We will also explore other emerging strategies, including cytokine therapy, gut microbiota modulation, and metal ion intervention, detailing the mechanistic rationale and landmark clinical trial data for each approach.

### 3.1 ICI Monotherapy

Following the approval of the initial ICI (the anti‐CTLA‐4 antibody ipilimumab) in 2011 [140], the indications for ICIs have rapidly expanded, making them an important treatment modality for various malignancies. The subsequent emergence of anti‐PD‐1 monoclonal antibodies has further enhanced therapeutic efficacy. Nivolumab and pembrolizumab were the first two PD‐1 inhibitors to be marketed. In the CheckMate‐066 trial evaluating first‐line therapy for advanced melanoma, nivolumab demonstrated a substantial survival benefit over conventional chemotherapy. The median OS was 37.5 months in the nivolumab arm versus 11.2 months in the chemotherapy group. Additionally, the 3‐year OS rate reached 52% with nivolumab, highlighting its superior long‐term efficacy [141]. In the treatment of NSCLC, nivolumab has shown superior survival outcomes compared with docetaxel in two landmark Phase III trials (CheckMate‐017 and CheckMate‐057), encompassing both squamous and nonsquamous histologies. Among patients with squamous cell carcinoma, nivolumab extended median OS from 6.0 months with docetaxel to 9.2 months (HR ≈ 0.59) [142]. In the KEYNOTE‐006 study, pembrolizumab demonstrated higher response rates and survival advantages compared with ipilimumab for the treatment of melanoma, leading to its approval for melanoma in 2014 [143]. The KEYNOTE‐024 trial marked a transformative shift in first‐line NSCLC treatment by demonstrating pembrolizumab's efficacy in patients with advanced disease and high PD‐L1 expression (defined as tumor proportion score, TPS ≥ 50%). The results indicated that pembrolizumab monotherapy achieved a median OS of 26.3 months, which was significantly better than the 13.4 months observed in the chemotherapy group (HR = 0.62) [144]. PD‐L1 inhibitors, exemplified by atezolizumab, have demonstrated efficacy across various tumor types. The OAK trial, conducted with NSCLC patients who had previously failed treatment, revealed that atezolizumab monotherapy as a second‐line treatment extended the median OS to 13.8 months, a significant improvement compared with 9.6 months in the control group (HR = 0.74, *p* < 0.001) [145].

Overall, the indications for ICI monotherapy have expanded to encompass dozens of tumors, significantly altering the treatment landscape for advanced cancers. ICI monotherapy has demonstrated durable tumor control and long‐term survival in many patients. However, the overall response rate and the applicable population remain limited, which poses one of the major challenges currently faced. In most solid tumors, the objective response rate (ORR) of PD‐1/PD‐L1 monoclonal antibody monotherapy ranges from 15 to 40%. For instance, the ORR is approximately 30–45% in melanoma [143], 15–20% in NSCLC [142], about 13% in HNSCC [146], and 22–25% in RCC [147]. This suggests that more than half, or even the majority, of patients still exhibit no significant response to monotherapy. Even within the subgroup of patients with high PD‐L1 expression, clinical observations have revealed that over 50% do not respond to PD‐1/PD‐L1 blockade [144]. Furthermore, even among those who initially respond favorably, some patients subsequently develop acquired resistance, leading to disease progression. For example, in long‐term follow‐up studies of melanoma, while approximately 25.8% of patients achieved complete remission [148], some of these individuals experienced disease recurrence several years later, necessitating further therapeutic intervention.

**FIGURE 3:**
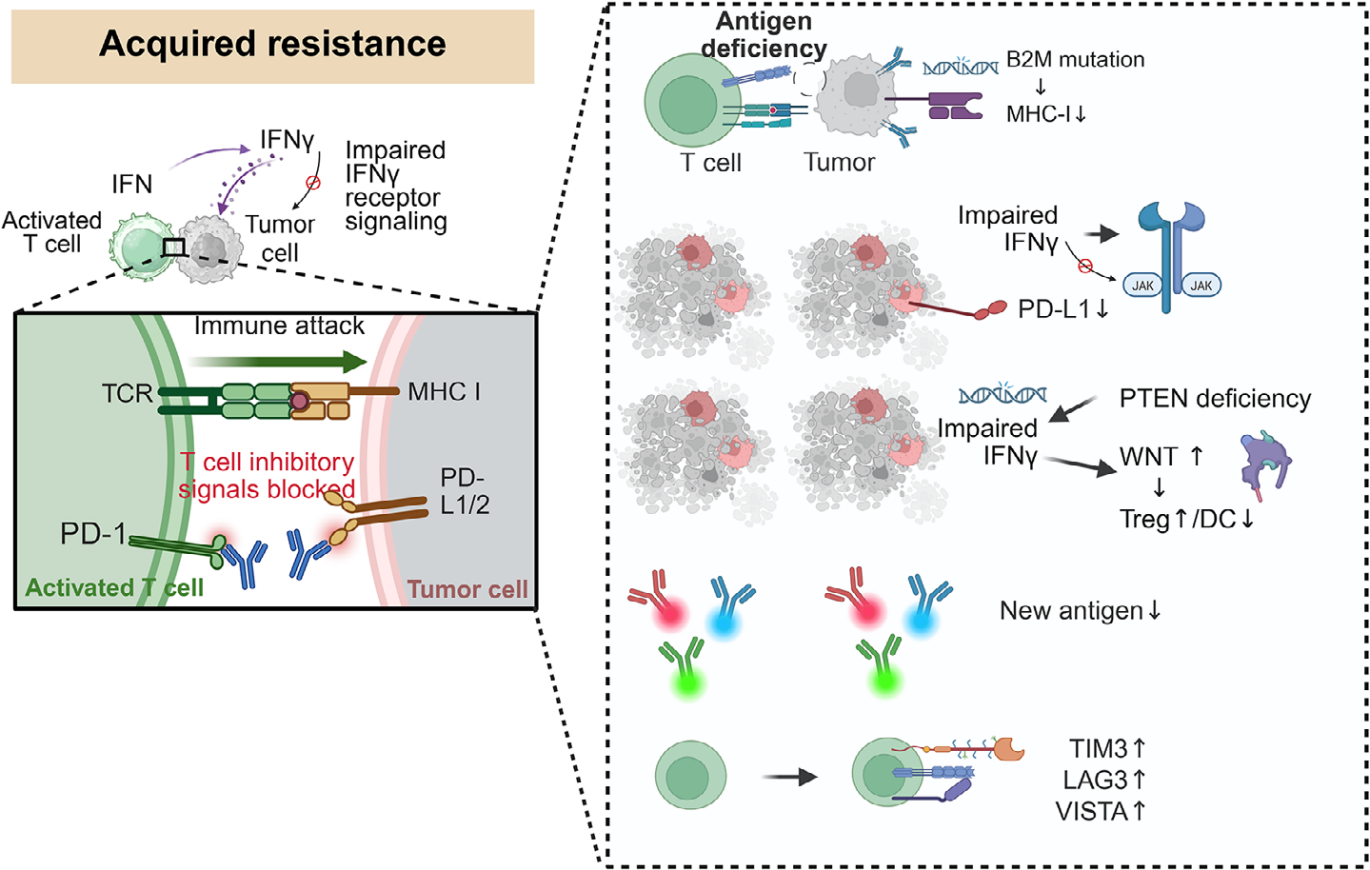
Mechanisms of acquired resistance to immune checkpoint inhibitors (ICIs). In the initial response to ICI therapy, T cells recognize tumor cells through antigen presentation by MHC‐I and exert immune attack by blocking PD‐1/PD‐L1 inhibitory signaling. However, tumor cells can develop various mechanisms of acquired resistance over time. These include: (1) antigen presentation defects due to B2M mutations, leading to downregulation of MHC‐I and impaired T cell recognition; (2) defects in IFN‐γ signaling, such as JAK1/JAK2 inactivation, resulting in impaired PD‐L1 signaling; (3) immune suppressive remodeling driven by loss of PTEN or activation of WNT, promoting accumulation of regulatory T cells (Treg) and rejection of dendritic cells (DC); (4) loss of neoantigens due to clonal evolution or epigenetic silencing, leading to immune evasion; (5) upregulation of alternative inhibitory immune checkpoints such as TIM‐3, LAG‐3, and VISTA, resulting in T cell exhaustion and persistent immune suppression (created in https://BioRender.com).

Despite initial therapeutic responses, many patients eventually develop resistance to ICI monotherapy (Figure 3). Reported mechanisms include: (1) *β2‐microglobulin (B2M) mutations*. Loss‐of‐function mutations in B2M, a critical component of MHC‐I complex assembly, disrupt cell‐surface MHC‐I expression. This defect impairs the presentation of tumor neoantigens to CD8^+^ T cells, leading to evasion of cytotoxic T lymphocyte‐mediated killing [149]. (2) *Defects in IFN‐γ signaling*. In colorectal cancer, subclonal inactivation of the IFN‐γ pathway reduces MHC expression and T cell infiltration. Polycomb repressive complex 2‐deficient tumors epigenetically silence IFN‐γ‐responsive genes, creating an immune‐desert phenotype. These defects diminish tumor immunogenicity, facilitating immune escape. (3) *Phosphatase and tensin homolog (PTEN) loss or wingless‐related integration site (Wnt) activation*. PTEN deficiency actively remodels the TME by inducing TGF‐β/CXCL10 secretion, which promotes Treg‐mediated immunosuppression and fibrosis, ultimately rendering tumors refractory to anti‐PD‐1 therapy [150]. Similarly, aberrant Wnt activation impairs immune surveillance and exacerbates an inflammatory, immunosuppressive state [151]. (4) *Loss of neoantigens*. Clonal evolution or epigenetic silencing reduces tumor immunogenicity, impairing T cell cytotoxicity. Gong et al. discovered that, in aging lung adenocarcinoma patients, diminished neoantigen burden correlates with reduced cytolytic activity despite increased CD8^+^T cell infiltration, while immunosuppressive elements (Tregs, TIM‐3, TIGIT) accumulate, fostering an immune‐evasive microenvironment unresponsive to PD‐1/PD‐L1 blockade [152]. (5) *Upregulation of alternative immune checkpoints*. TIM‐3, LAG‐3, and VISTA drive resistance by inducing T cell exhaustion [153]. Soluble TIM‐3 (sTIM‐3) exacerbates exhaustion via CEACAM‐1, while LAG‐3 synergizes with PD‐1 to sustain TOX‐mediated dysfunction and suppress IFN‐γ responses [154]. Dual blockade (e.g., anti‐LAG‐3 plus anti‐PD‐1) restores CD8^+^T cell clonality and effector function, highlighting coinhibitory pathways as promising therapeutic targets [153]. (6) *Nonimmune resistance driven by intracellular PD‐L1 isoforms*. As detailed in Section 2.2, beyond its checkpoint function, cPD‐L1 promotes tumor proliferation and resistance to DNA damage‐inducing therapies, suggesting it could be a novel target to sensitize tumors to chemoradiotherapy.

### 3.2 Combination Therapies With ICIs

#### 3.2.1 Combination of Multiple ICIs

**FIGURE 4:**
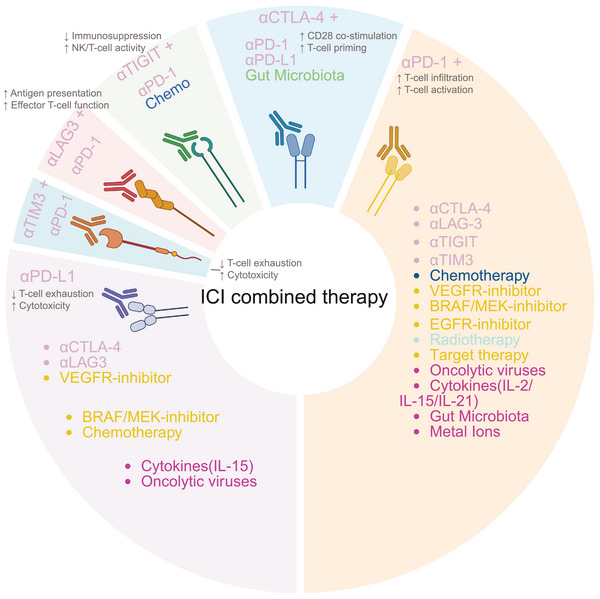
Overview of the classification of combination therapy strategies with ICIs. This figure summarizes the primary combination therapy strategies involving ICIs, such as anti‐PD‐1, anti‐PD‐L1, and anti‐CTLA‐4, in both clinical and research settings. Common combination approaches include: (1) combining with other immune checkpoint antibodies, such as anti‐CTLA‐4, anti‐LAG‐3, anti‐TIGIT, and anti‐TIM‐3; (2) combining with traditional treatment methods, including chemotherapy and radiotherapy; (3) combining with targeted therapeutic drugs, encompassing inhibitors of pathways such as VEGFR, BRAF/MEK, and EGFR; (4) combining with novel therapies, such as cytokines (IL‐2/15/21), gut microbiota modulation, metal ion intervention, and oncolytic viruses. These strategies enhance the antitumor immune response through synergy, improve the tumor immune microenvironment, and reverse immune tolerance, thereby increasing the efficacy and clinical benefit rate of ICIs (created in https://BioRender.com).

To address the low therapeutic efficiency and drug resistance in monotherapy, multiple combination strategies were studied (Figure 4 and Table 1). While Table 1 indicates that ICI combinations generally outperform monotherapies, direct cross‐trial comparisons are limited by substantial heterogeneity. This variability primarily stems from HCC's intrinsically immunosuppressive microenvironment, disparate trial designs (e.g., active comparators vs. single‐arm), and diverse patient baselines. Consequently, these findings underscore the urgent clinical need for validated predictive biomarkers to guide personalized treatment.

**TABLE 1:** Summary of clinical trials investigating immune checkpoint inhibitors and combination therapies in cancers.

Drugs	Drug type	Cancer type	Control regimen	Phase	NCT number	ORR^a^ (%)	DCR^b^ (%)	mPFS^c^ (months)	mOS^d^ (months)
**Monotherapy**									
Tremelimumab	Anti‐CTLA‐4	Hepatocellular carcinoma	No control (single‐arm)	Phase II	NCT01008358	NA	76.4	6.5	8.2
Nivolumab	Anti‐PD‐1	Hepatocellular carcinoma	No control (dose‐escalation)	Phase I/II	NCT01658878	15/20	58/64	3.4/4.0	15.0/NR
		—	Sorafenib	Phase III	NCT02576509	NA	NA	NA	16.4
Pembrolizumab	Anti‐PD‐1	Hepatocellular carcinoma	No control	Phase II	NCT02702414	17	62	4.9	12.9
		—	Placebo + BSC	Phase III	NCT02702401	18.3	62.2	3	13.9
**ICIs combinations**									
Nivolumab + ipilimumab	Anti‐PD‐1 + anti‐CTLA‐4	Hepatocellular carcinoma	Nivolumab monotherapy	Phase I/II	NCT01658878	31	49	NA	22.8
Durvalumab + tremelimumab	Anti‐PD‐1 + anti‐CTLA‐4	Hepatocellular carcinoma	Sorafenib	Phase III	NCT03298451	20.1	60.1	3.8	16.4
**ICIs combined with targeted drugs**									
Atezolizumab + bevacizumab	Anti‐PD‐1 + anti‐VEGF	Hepatocellular carcinoma	Sorafenib	Phase III	NCT03434379	27.3	NA	6.8	NR
Pembrolizumab + lenvatinib	Anti‐PD‐1 + TKIs	Hepatocellular carcinoma	No control	Phase I	NCT03006926	46	NA	9.3	22
Nivolumab + lenvatinib	Anti‐PD‐1 + TKIs	Hepatocellular carcinoma	No control	Phase I	NCT03418922	76.7	96.7	NA	NA
Sintilimab + IBI305	Anti‐PD‐1 + anti‐VEGF	Hepatocellular carcinoma	Sorafenib	Phase II/III	NCT03794440	NA	NA	4.6	NR
Camrelizumab + apatinib	Anti‐PD‐1 + TKIs	Hepatocellular carcinoma	Sorafenib	Phase III	NCT03764293	33.1	78.3	5.6	22.1

Data sources from clinical registration website.

^a^ Objective response rate.

^b^ Disease control rate.

^c^ Median progression‐free survival.

^d^ Median overall survival.

Mechanistically, CTLA‐4 functions as a “brake” during T cell priming in secondary lymphoid organs, whereas the PD‐1 axis predominantly restricts effector T cells within peripheral tissues. Initial investigations revealed that monotherapy targeting either checkpoint, while augmenting T cell activity, often triggers compensatory upregulation of the alternative inhibitory pathway, thereby limiting durable antitumor immunity [155]. This underscores the rationale for dual blockade: CTLA‐4 inhibition broadens the peripheral T cell repertoire, while PD‐1 blockade acts within the peripheral TME to reinvigorate exhausted effector T cells. Synergistic targeting of these nonredundant pathways disrupts both compensatory and primary immunosuppressive circuits, promoting enhanced intratumoral T cell infiltration, proinflammatory cytokine production, and robust antitumor responses.

Building upon these mechanistic insights, the first clinical evidence supporting the efficacy and safety of nivolumab + ipilimumab combination blockade in melanoma patients was reported in 2013 [156]. The ORR of the combination therapy was approximately 40%. However, at the recommended dose (nivolumab 1 mg/kg + ipilimumab 3 mg/kg), the ORR could reach about 53%, with some patients experiencing tumor shrinkage of ≥80%. This finding demonstrates that the combination of the PD‐1 inhibitor and the CTLA‐4 inhibitor exhibits strong antitumor activity in clinical practice and is both safe and manageable. The 5‐year follow‐up results from the CheckMate 067 Phase III study [157], which compared three treatment groups, nivolumab + ipilimumab, nivolumab monotherapy, and ipilimumab monotherapy, demonstrated differential survival outcomes among treatment arms, with 5‐year OS rates of 52% for combination therapy (nivolumab plus ipilimumab), 44% for nivolumab monotherapy, and 26% for ipilimumab monotherapy. These results indicate that the combination therapy significantly prolongs survival in advanced melanoma patients, with a 5‐year survival rate exceeding 50% (52%). In 2025, Wolchok et al. reported the final 10‐year follow‐up results of the CheckMate 067 trial [158]. The median OS in the combination group was approximately 71.9 months (nearly 6 years), with 37% of patients still alive at the 10‐year mark. The median OS was significantly longer with nivolumab monotherapy (36.9 months) compared with ipilimumab (19.9 months). The final trial results demonstrated that for patients with advanced melanoma, both nivolumab combined with ipilimumab and nivolumab monotherapy provided sustained survival benefits compared with ipilimumab monotherapy.

The combined blockade of PD‐1/PD‐L1 and CTLA‐4 immune checkpoints has received regulatory approval for multiple malignancies, notably including melanoma and RCC (CheckMate 214, Phase III) [159], colorectal cancer (CheckMate 8HW, Phase III) [15], HCC (CheckMate 040, Phase III) [160], and esophageal squamous cell carcinoma (CheckMate 648, Phase III) [161]. Dual ICIs employing durvalumab (PD‐L1) and tremelimumab (CTLA‐4) represent an emerging therapeutic approach for hepatic malignancies [162], while in lung cancer, dual ICIs combinations demonstrate promising potential. For instance, in the CheckMate 012 trial (Phase I) [163], the combination of nivolumab at a dose of 3 mg/kg every 2 weeks and ipilimumab at 1 mg/kg every 12 weeks or every 6 weeks in NSCLC yielded remarkable results, with an ORR of 43%, compared with 23% for monotherapy. In the CheckMate 568 trial (Phase II) [164], among 288 previously untreated advanced NSCLC patients, the overall response rate reached 30%, with stratification revealing differential efficacy based on PD‐L1 status (41% ORR for PD‐L1 ≥1% cohort compared with 15% ORR for PD‐L1<1% subgroup). Furthermore, the CheckMate 227 trial (Phase III) [165] confirmed that for patients with PD‐L1 expression ≥1%, dual ICI therapy increased OS from 14.9 months in the chemotherapy group to 17.1 months, with a 2‐year survival rate of 49 versus 11%. For patients with PD‐L1<1%, OS was also extended by 5 months compared with chemotherapy, and those with tumor mutational burden (TMB) ≥10 mutations/Mb experienced even greater benefits. This evidence supports the efficacy of dual ICI therapy in populations with low PD‐L1 expression.

Research indicates that the expression of TIGIT is closely associated with CD8^+^ T cell infiltration and PD‐1 expression. Mechanistically, PD‐1 and TIGIT can inhibit the activity of the costimulatory molecule CD226 through distinct pathways: TIGIT blocks its activation by competitively binding to its ligand, while PD‐1 induces CD226 dephosphorylation by recruiting SHP2. In comparison, anti‐PD‐1 therapy is more effective than anti‐TIGIT in activating CD226. When both are coexpressed, the activity of CD226 is significantly reduced, suggesting that only the combined blockade of PD‐1 and TIGIT can fully restore CD226 function, thereby achieving effective T cell activation [166].

The combination therapy of PD‐1/PD‐L1 with third‐generation ICIs is currently being actively explored. Among these, the LAG‐3 inhibitor relatlimab was approved in 2022 for the treatment of advanced melanoma [167], marking it as the first marketed LAG‐3 antibody. The RELATIVITY‐047 trial (Phase II/III) established that the relatlimab‐nivolumab combination significantly improved progression‐free survival (PFS) in advanced melanoma patients versus nivolumab monotherapy, with benefits observed across all PD‐L1 and LAG‐3 expression subgroups. This combination regimen is now being further investigated in adjuvant and neoadjuvant therapy (NCT05002569, NCT05418972). An indirect comparative study based on RELATIVITY‐047 (nivolumab + relatlimab) versus CheckMate 067 (nivolumab + ipilimumab) indicated that the overall population exhibited approximately equivalent PFS, confirmed ORR and OS [168]. However, for specific subgroups, such as acral melanoma, B‐Raf proto‐oncogene, serine/threonine kinase (BRAF) mutation, and lactate dehydrogenase levels greater than twice the upper limit of normal, nivolumab + ipilimumab may demonstrate a more favorable trend. Conversely, nivolumab + relatlimab was associated with significantly fewer adverse reactions and a lower discontinuation rate. Both regimens have similar efficacy, and nivolumab + relatlimab has a superior safety profile, with fewer Grade 3–4 irAEs (23 vs. 61%) and discontinuations (17 vs. 41%) [168]. Nevertheless, the results should be interpreted with caution and are intended for reference only.

In the research on the TIGIT pathway, the combination of vibostolimab and pembrolizumab (MK‐7684A) [169, 170], which serves as the core drug of the KEYVIBE series, has been undergoing clinical trials across multiple indications. However, the current research results have generally fallen short of expectations. In studies involving advanced NSCLC (KEYVIBE‐002), adjuvant therapy for high‐risk melanoma (KEYVIBE‐010), relapsed or refractory lymphoma (KEYVIBE‐004), advanced cervical cancer (KEYVIBE‐005), and extensive‐stage small cell lung cancer (KEYVIBE‐008), the primary efficacy endpoints were not achieved, and some trial groups experienced higher rates of adverse events. Currently, three lung cancer‐related studies (KEYVIBE‐003, ‐006, ‐007, KEYVIBE‐008), and one high‐risk melanoma (KEYVIBE‐010) are ongoing. In addition to MK‐7684A, other TIGIT‐targeting agents in pivotal clinical stages include domvanalimab [171], ociperlimab (NCT04693234), rilvegostomig (NCT06868277, NCT04995523), and belrestotug (NCT06472076), with indications covering gastric cancer, cervical cancer, biliary tract cancer, bladder cancer, and esophageal cancer [172, 173].

Early‐phase clinical trials have shown promising antitumor activity with TIM‐3/PD‐1 dual blockade, leading to accelerated development of several investigational agents. Notable candidates in active clinical evaluation include verzistobart (AZD7789) and cobolimab (GSK4069889), which are currently being advanced through the development pipeline. Notably, the overall efficacy of third‐generation ICIs combination regimens remains limited, possibly due to overlapping mechanisms of action with PD‐1/PD‐L1 [174]. In contrast, the combination of ipilimumab and nivolumab, which enhances the regulation of T cell initiation and activation, remains the most effective combination regimen. This suggests that future immune therapy pairings should be prioritized based on mechanistic complementarity.

The development of “third‐generation” immune checkpoints such as TIGIT, LAG‐3, and TIM‐3 was initially driven by a straightforward premise: combining them with PD‐1/PD‐L1 inhibitors would yield superior efficacy. However, the clinical results have been decidedly mixed, prompting a critical reassessment of this combinatorial strategy.

The recent setbacks with TIGIT targeted therapies clearly illustrate the challenges of combination strategies. While preclinical data were promising, the failure of several Phase III trials, such as the KEYVIBE series, highlights a stark translational gap. This disconnect largely stems from two key issues: target selection bias and mechanistic redundancy. TIGIT blockade relies on unleashing the costimulatory receptor CD226. However, terminally exhausted CD8^+^ T cells in the human TME often downregulate CD226, meaning TIGIT blockade fails to provide positive costimulation and makes the combination with anti‐PD‐1 functionally redundant rather than synergistic [175, 176]. Second is the functional paradox of the Fc domain in antibody design. TIGIT is highly expressed on both effector T cells and immunosuppressive Tregs [129]. An active Fc domain (e.g., IgG1) can deplete intratumoral Tregs via ADCC but risks depleting CD8+ effectors, while an Fc‐silent domain (e.g., IgG4) preserves effectors but sacrifices Treg depletion [177]. The failure to balance this trade‐off, coupled with TIGIT's complex expression profile, likely drove the inconsistent efficacy observed in pivotal trials. Ultimately, future combination strategies must move beyond simply stacking targets and instead focus on specific, nonredundant resistance pathways.

In contrast, the approval of the LAG‐3 inhibitor relatlimab provided a new, less toxic therapeutic option. Nonetheless, its PFS benefit was modest (HR = 0.75), particularly when compared with the high bar set by CTLA‐4 combinations [168]. These divergent outcomes underscore a critical lesson: the future of immuno‐oncology lies not in empirically “stacking” targets, but in rationally pairing them based on a deep mechanistic understanding. The relative success of LAG‐3 blockade likely stems from its complementary mechanism to PD‐1, whereas the failure of TIGIT strategies warns that targeting functionally redundant pathways offers limited clinical gain. Future efforts must, therefore, evolve from broad combination approaches to synergistic designs guided by specific resistance pathways.

#### 3.2.2 ICIs Combined With Chemotherapy

Chemotherapy not only exerts direct cytotoxic effects on tumor cells but also synergizes with ICIs through various immunomodulatory mechanisms: (1) *Induction of ICD*: Chemotherapy promotes the release of damage‐associated molecular patterns, such as ATP and HMGB1, which activate APCs, enhance tumor antigen recognition, and initiate adaptive immune responses [178]. (2) *Enhancement of antigen presentation capacity*: Certain chemotherapeutic agents can upregulate MHC molecules and antigen processing pathways, thereby increasing the recognizability of tumor cells by T cells [179]. (3) *Elimination of immunosuppressive cells*: Chemotherapy can significantly reduce Tregs, immunosuppressive macrophages, and MDSCs, thereby contributing to the remodeling of the immune microenvironment and enhancing immune responses [180]. (4) *Regulating gene expression*: Epigenetic regulators, such as histone deacetylase (HDAC) or DNA methyltransferase inhibitors [181], can activate antigen processing and costimulation pathways, thereby enhancing immune responses. (5) *Restoring or enhancing chemotherapy sensitivity*: Some studies have found that immunotherapy can reverse chemotherapy resistance, improving tumor response to subsequent chemotherapy [182]. A critical consideration in chemo‐immunotherapy is that not all chemotherapeutic agents offer the same synergistic potential. The ideal agent should induce ICD to stimulate an immune response, while carefully avoiding significant lymphodepletion that would undermine concomitant immunotherapy [178]. This is why agents like anthracyclines and oxaliplatin, known for their potent ICD induction, are such attractive partners. The primary challenge in clinical translation, then, is finding the therapeutic sweet spot: a dosing regimen that achieves robust tumor cytotoxicity and antigen release while preserving the patient's immune competence, thereby enabling chemotherapy to serve as an effective immune primer. Recently, the study by Zhang et al. [183] further elucidated the mechanistic differences between chemotherapy combined with immunotherapy in TNBC. Comparative analysis revealed that the nab‐paclitaxel/atezolizumab combination, relative to conventional paclitaxel, induced significant expansion of TCF7‐expressing stem‐like effector memory CD8^+^T cells and Tfh. This immunomodulatory effect was associated with durable antitumor immunity. Furthermore, the regimen promoted polarization of proinflammatory macrophage subsets, CXCL9+ and folate receptor beta (FOLR2)+ phenotypes, enhancing intratumoral immune activation. Nab‐paclitaxel significantly upregulates mast cells and enhances T/B cell infiltration. Further experiments indicate that the combined activation of mast cells with PD‐L1 inhibition markedly suppresses tumor progression. This study underscores the critical impact of chemotherapy drug selection on the efficacy of combination immunotherapy and, for the first time, proposes mast cells as a potential emerging target for immunotherapy, providing a new direction for personalized treatment of TNBC.

In 2019, KEYNOTE‐021 first reported the efficacy of pembrolizumab combined with chemotherapy as a first‐line treatment compared with chemotherapy alone in metastatic nonsquamous NSCLC [184]. The combination therapy demonstrated a significantly higher ORR of 55% compared with 29% in the chemotherapy‐only arm (*p* < 0.05); the median PFS was also longer (13.0 vs. 8.9 months). Subsequently, KEYNOTE‐189 further validated the survival benefits of this regimen. The combination therapy significantly improved OS, PFS, and ORR relative to single‐agent chemotherapy, while also showing improvement or maintenance in quality of life (with an improvement in Global Health Status/Quality of Life score from baseline at Week 21, *p* = 0.014) and a trend toward delayed worsening of some symptoms. Based on these findings, pembrolizumab in combination with chemotherapy has been established as the first‐line standard treatment for advanced nonsquamous NSCLC [185]. In the KEYNOTE‐062 trial for gastric cancer, the combination therapy group (pembrolizumab plus chemotherapy) exhibited a median PFS that was not reached, which was significantly better than the 6.6 months reported in the chemotherapy‐alone group. The ORR achieved 64.7%, also surpassing the 36.8% observed in the chemotherapy group. Compared with pembrolizumab monotherapy, the combination therapy demonstrated further improvements in both PFS and ORR, indicating a synergistic effect between the two treatments [186]. The current treatment strategy for advanced inoperable gastric cancer primarily relies on a combination of chemotherapy and immunotherapy, guided by biomarkers, with the incorporation of other targeted therapies being a potential avenue for future research. Landmark Phase III trials have validated the therapeutic superiority of immunotherapy–chemotherapy combinations, establishing this approach as a cornerstone of first‐line treatment for HER2)‐negative advanced gastric adenocarcinoma [187, 188, 189, 190].

Two Phase III clinical trials, namely, EV‐302/KEYNOTE‐A39 [191] and CheckMate‐901 [192], assessed the effectiveness of combination immune therapy as a primary treatment for advanced urothelial carcinoma (UC). In the EV‐302 trial, 886 patients suffering from locally advanced or metastatic UC were enrolled. The outcomes demonstrated that the combination of enfortumab vedotin and pembrolizumab led to considerable improvements in median PFS (12.5 months compared with 6.3 months, HR = 0.45) and OS (31.5 vs. 16.1 months, HR = 0.47) relative to conventional chemotherapy. Moreover, the ORR and response duration were significantly higher than those seen with chemotherapy, all while keeping a manageable safety profile. This solidified the combination as a new benchmark for first‐line therapy in advanced UC. The CheckMate‐901 trial involved 608 patients with untreated unresectable or metastatic UC. The trial demonstrated statistically significant improvements with nivolumab plus chemotherapy versus chemotherapy alone in advanced UC: median OS (21.7 vs. 18.9 months; HR = 0.78) and PFS (7.9 vs. 7.6 months; HR = 0.72). These results supported the US FDA's approval of this combination as first‐line therapy for advanced UC.

#### 3.2.3 ICIs Combined With Targeted Therapy

**FIGURE 5:**
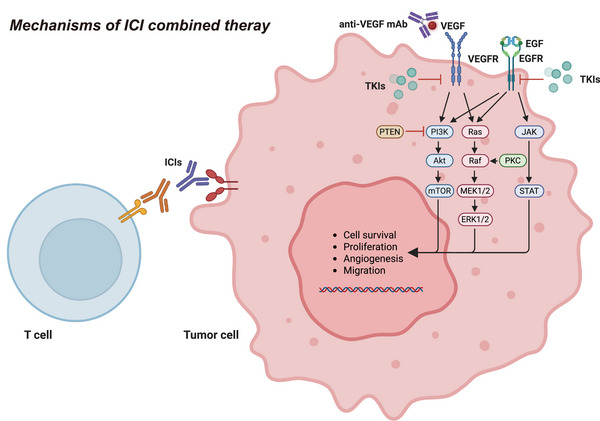
Schematic diagram of the mechanism of action of ICIs in combination therapy. This figure illustrates the synergistic mechanisms by which ICIs interact with multitargeted therapeutic strategies, such as VEGF‐targeting monoclonal antibodies and small‐molecule tyrosine kinase inhibitors (TKIs), within tumor cells. ICIs enhance the antitumor effects of T cells by blocking pathways like PD‐1/PD‐L1 and CTLA‐4, thereby alleviating the immune suppression experienced by T cells. Concurrently, antiangiogenic therapies, such as anti‐VEGF antibodies, and TKIs inhibit receptor‐mediated signaling pathways, including VEGFR and EGFR, which interferes with classical signaling cascades like PI3K–AKT–mTOR, RAS–RAF–MEK–ERK, and JAK–STAT. This disruption impedes the survival, proliferation, angiogenesis, and migration of tumor cells. The synergistic inhibition of multiple pathways enhances the tumor immune microenvironment and improves both the response rate and durability of ICI treatment (created in https://BioRender.com).

Targeted drugs not only exhibit direct antitumor effects but also induce ICD, thereby enhancing antigen presentation, eliminating immunosuppressive factors, and improving the efficacy of immunotherapy [193]. Multiple oncogenic signaling pathways are closely associated with the mechanisms of antigen presentation. For instance, the vascular endothelial growth factor (VEGF)–VEGFR pathway broadly influences immune cells by inhibiting T cell activation [194] and promoting the accumulation of immunosuppressive cells such as MDSCs [195]. In NSCLC, constitutive activation of EGFR and PI3K/AKT signaling pathways promotes immune evasion through upregulation of PD‐1/PD‐L1 immune checkpoint molecules [196]. Additionally, IDO‐mediated tryptophan metabolism inhibits the functions of effector T cells and NK cells, enhances the activities of Tregs and MDSCs, and promotes the polarization of macrophages toward an immunosuppressive phenotype [197]. The aforementioned mechanisms collectively illustrate the significant role of targeted drugs in modulating the tumor immune microenvironment, highlighting their potential to synergize with immunotherapy (Figure 5).

The biological rationale for combining antiangiogenic agents, such as bevacizumab or tyrosine kinase inhibitors (TKIs), with ICIs involves more than additive cytotoxic effects. VEGF‐driven aberrant vasculature contributes to a state of “endothelial anergy”, creating a physical barrier that limits T cell infiltration into tumors. By promoting vascular normalization, antiangiogenic therapy can help restore vessel integrity, improving the delivery of activated effector T cells to the TME following ICI treatment [198]. It should be noted, however, that combining ICIs with small‐molecule TKIs may result in overlapping toxicities, including elevated risk of hepatotoxicity, necessitating closer monitoring than with either agent alone.

Melanoma was one of the earliest cancers to which ICIs were applied, particularly PD‐1/PD‐L1 inhibitors. In the treatment of melanoma, the combination of ICIs with targeted therapy has made significant strides. The triplet regimen combining atezolizumab, vemurafenib, and cobimetinib has received regulatory approval for BRAF V600‐mutant advanced melanoma, demonstrating significant PFS and OS benefits in clinical trials [199, 200, 201, 202, 203]. According to data from the IMspire150 clinical trial, the efficacy of this triple‐drug combination surpasses that of traditional monotherapy [201].

In NSCLC, the combination of ICIs with targeted therapies has demonstrated varying clinical outcomes. Although the pairing of PD‐1 inhibitors with EGFR inhibitors has resulted in certain toxic reactions in some NSCLC patients, there has been no significant improvement in clinical efficacy [204, 205, 206]. For instance, the combination therapy of the PD‐1 inhibitor pembrolizumab with the EGFR inhibitor gefitinib in patients with EGFR mutations did not significantly enhance treatment outcomes and exhibited poor tolerability due to the high incidence of hepatic toxicity and pneumonitis [207]. Conversely, the combination regimen of PD‐L1 inhibitors and antiangiogenic drugs, such as the concurrent use of atezolizumab and bevacizumab, has demonstrated significant improvements in PFS and OS in NSCLC patients [208, 209].

In HCC, the combination of ICIs with targeted therapy has yielded relatively positive clinical outcomes [210]. The bevacizumab‐atezolizumab combination is approved for advanced HCC. The IMbrave150 trial established that the atezolizumab–bevacizumab regimen significantly prolonged both median OS and PFS compared with sorafenib monotherapy. Compared with sorafenib alone, the combination therapy group achieved a 1‐year OS rate of 67.2% [198].

In the treatment of urological tumors, the combination of ICIs and targeted therapy has shown varying prospects. Particularly in RCC, the combination of PD‐1 inhibitors and antiangiogenic agents, including bevacizumab, has been established as a first‐line therapeutic approach. Evidence from pivotal trials such as IMmotion151 and IMbrave150 indicates that the atezolizumab–bevacizumab regimen significantly enhances PFS in RCC and demonstrates improved clinical outcomes, particularly in PD‐L1‐positive subgroups [211, 212]. In summary, the combination of ICIs and targeted therapies has demonstrated potential across various types of tumors, particularly in the treatment of melanoma, NSCLC, HCC, and urological tumors. However, the differences in efficacy among various tumor types and treatment combinations, along with the accompanying side effects, remain critical challenges in current research and clinical treatment.

#### 3.2.4 ICIs Combined With OVs

OVs offer a targeted therapeutic approach, exploiting their ability to selectively replicate in and kill malignant cells. Through direct tumor cell lysis, these agents induce ICD, releasing tumor‐specific antigens that stimulate systemic antitumor immune responses. Nevertheless, numerous malignancies develop immune evasion mechanisms by suppressing immune activity via the expression of immune checkpoint proteins, including PD‐1 and CTLA‐4. ICIs restore T cell function by blocking these immune checkpoints. Consequently, the combined application of OVs and ICIs can synergistically enhance antitumor immune responses: OVs increase the exposure of tumor antigens and the infiltration of immune cells, while ICIs relieve immune suppression, thereby improving treatment efficacy.

In melanoma therapy, OVs combined with ICIs demonstrate promising efficacy. For instance, Coxsackievirus A21, commercially known as Cavatak, is an OV that has been utilized in conjunction with ICIs across multiple clinical trials [213, 214]. In the CAPRA clinical trial (NCT02565992), Cavatak was combined with pembrolizumab in patients with advanced melanoma, demonstrating enhanced antitumor activity and an acceptable safety profile [215]. In NSCLC, the therapeutic potential of combining OVs with ICIs is still under preliminary investigation. Multiple ongoing clinical trials are assessing both the safety profile and antitumor efficacy of this dual‐modality approach. For example, the STORM clinical trial (NCT02043665) is investigating the effects of combining Cavatak with pembrolizumab in NSCLC patients, and preliminary results indicate some clinical benefits [216]. For HCC, OV plus ICI therapy demonstrates promising efficacy. A Phase I clinical trial (NCT03764787) evaluated the safety and efficacy of the oncolytic adenovirus H101 in conjunction with nivolumab in advanced HCC patients who had previously undergone systemic treatment [217]. The results indicated that this combination therapy exhibited an acceptable safety profile, with antitumor activity in some patients. In the field of urological tumors, the combined research of OVs and ICIs remains in the exploratory phase. Clinical trial CANON (NCT02316171) investigated the safety profile and preliminary therapeutic outcomes of Cavatak, used either alone or concomitantly with mitomycin C, in patients diagnosed with nonmuscle invasive bladder cancer [217]. The results indicated that Cavatak could replicate in bladder cancer tissues and elicit an antitumor immune response, suggesting that its combined application with ICIs may yield a synergistic effect.

The combination therapy of OVs and ICIs has shown potential clinical benefits across various cancer types. However, further large‐scale clinical trials are required to validate their efficacy and safety, as well as to optimize treatment protocols, thereby providing patients with more effective therapeutic options.

#### 3.2.5 ICIs Combined With Radiotherapy

Radiotherapy not only directly kills tumor cells through DNA damage but also significantly remodels the TME, thereby enhancing the efficacy of ICIs [218]. The primary mechanisms involved include: (1) Radiotherapy can trigger ICD, inducing tumor‐associated antigen release, and promote DC activation, which initiates T cell responses and enhances the effectiveness of ICIs; (2) by remodeling the tumor physical microenvironment, radiotherapy can reduce interstitial fluid pressure, decrease solid stress, and degrade collagen fibers, thereby improving the distribution and penetration of ICIs within tumor tissues [219, 220, 221]. (3) Furthermore, radiotherapy enhances vascular permeability and promotes T cell infiltration [222], particularly the recruitment of CD8^+^T cells, thereby augmenting both local and systemic antitumor responses (i.e., the “abscopal effect”). (4) Additionally, radiotherapy reduces Tregs and MDSCs, while inducing the transformation of TAMs into the M1 phenotype, thereby reversing the immunosuppressive TME [221, 223].

In clinical studies that combine radiotherapy with ICIs, the CHEERS study (*JAMA Oncol*, 2023) stands out as a significant Phase II multicenter randomized controlled trial [224]. This study evaluated the effectiveness of ICI monotherapy versus ICI combined with stereotactic body radiotherapy (SBRT, 3×8 Gy) in patients with locally advanced or metastatic solid tumors, such as RCC, UC, NSCLC, and melanoma. Median PFS was significantly prolonged with combination therapy (4.4 months) compared with monotherapy (2.8 months), though this difference lacked statistical significance (HR = 0.95, *p* = 0.82) [224]. The OS was reported as 14.3 months for the combination therapy group and 11.0 months for the monotherapy group, with no significant difference noted (HR = 0.82, *p* = 0.47) [224]. Nonetheless, the local control rate in the combination therapy group reached 75%, suggesting that SBRT plays a crucial role in controlling local lesions. Importantly, this treatment did not lead to an increase in grade 3 or higher toxicity, indicating that it is generally safe and manageable. Although the primary endpoint did not demonstrate significant improvement, this study still provides clinical evidence for the adjuvant role of radiotherapy in immunotherapy, particularly within the oligometastatic setting. Various radiotherapy strategies, in terms of dosage and fractionation (for example, SBRT versus low‐dose total body irradiation), have a considerable impact on the activation of immune responses [225]. Some studies have even indicated that radiotherapy can convert the originally “cold” into a “hot” immune environment, thereby enhancing the therapeutic response rate of ICIs [226, 227, 228].

#### 3.2.6 ICIs Combined With Other Strategies

##### 3.2.6.1 Cytokines

In recent years, cytokines have been extensively studied as immunomodulators, demonstrating significant potential in cancer immunotherapy. Recombinant IL‐2 enhances the antitumor activity of CD8^+^T cells and NK cells through activation of the intermediate‐affinity IL‐2 receptor β and γ subunits [229, 230]. Currently, various “next‐generation” IL‐2 derivatives are undergoing Phase I clinical trials aimed at enhancing specificity and reducing toxicity [231, 232]. Early studies indicate that these IL‐2 derivatives exhibit clinical activity both as monotherapy and in combination with anti‐PD‐1 therapy across multiple tumor types, with related Phase I and III clinical trials underway for NSCLC (NCT04830124) [233] and ovarian cancer (NCT05092360) [234], respectively.

In addition to IL‐2, cytokines such as IL‐15 and IL‐21 are frequently used to augment the expansion, persistence, and memory development of T lymphocytes and natural killer cells, thereby enhancing tumoricidal activity [235, 236, 237]. Engineered cytokines or cytokine‐antibody fusion proteins, such as KD033, which combines a PD‐L1 monoclonal antibody with IL‐15, are currently under evaluation in Phase I clinical trials [238, 239]. These trials aim to achieve tumor‐targeted immune activation while minimizing systemic toxicity. Furthermore, the regulation of cytokine signaling pathways represents a crucial direction in combination strategies for immunotherapy.

##### 3.2.6.2 Gut Microbiota

Recent studies have demonstrated that the gut microbiota can activate innate immune pathways (such as STING) and adaptive immune responses through metabolites (including short‐chain fatty acids, indole derivatives, and inosine) and interactions with host immune cells [240, 241, 242], thereby enhancing T cell activity and improving the efficacy of ICIs. Furthermore, certain microbiota can further activate antitumor immunity by modulating antigen presentation, promoting IFN‐γ secretion, and inhibiting immunosuppressive cells such as Tregs and MDSCs [243, 244, 245]. Multiple studies have identified core microbiota closely associated with the efficacy of ICIs: Akkermansia muciniphila, Faecalibacterium prausnitzii, and Bifidobacterium longum are abundant in responders, while Bacteroides and Escherichia coli are more common in nonresponders [246, 247, 248, 249]. The use of antibiotics has been shown to diminish the efficacy of ICIs, indicating that the stability of the gut microbiome is crucial for the success of treatment.

Current therapeutic enhancement approaches incorporate fecal microbiota transplantation (FMT), probiotic administration, prebiotic supplementation, and genetically modified bacteria. Among these, FMT combined with PD‐1 inhibitors has shown significant promise in clinical trials for solid tumors, maintaining a favorable safety profile while demonstrating efficacy in melanoma patients [250, 251, 252]. This combination has successfully overcome anti‐PD‐1 resistance in previously refractory patients. Notably, FMT induced favorable shifts in the immune cell composition and gene expression profiles within both the gut lamina propria and the TME [252].

Beyond FMT, other interventions are also being explored. For example, the live bacterial preparation Clostridium butyricum, when combined with nivolumab/ipilimumab, has significantly prolonged PFS in RCC cancer patients [253]. Similarly, nutritional strategies are gaining traction; studies have indicated that a high dietary fiber intake promotes the proliferation of beneficial bacteria and enhances the response to ICIs [254]. In summary, the gut microbiota not only serves as a predictor for the efficacy of ICIs but is also emerging as a key target for intervention to enhance immunotherapy response rates. This paves the way for precise and personalized “microbiota + immune” combination therapy strategies in the future.

##### 3.2.6.3 Metal Ions

Metal ions can enhance the therapeutic efficacy of ICIs by modulating the tumor immune microenvironment, thereby emerging as a novel combination therapy strategy. Different metal ions possess unique immunomodulatory mechanisms: Manganese ions (Mn^2^^+^) activate the cGAS–STING pathway, enhancing DC maturation and CD8^+^ T cell activity [255]; iron ions (Fe^2^^+^) induce tumor cell ferroptosis to release tumor antigens while regulating M1/M2 macrophage polarization [256, 257]; elevated levels of copper (Cu^2^^+^) can induce PD‐L1 expression, but immune suppression can be reversed through copper chelation or ionophores [258, 259]; magnesium (Mg^2^^+^) maintains TCR signal transduction and T cell metabolic homeostasis [260]. Various animal models, including melanoma, NSCLC, and colorectal cancer, have confirmed their synergistic antitumor effects with ICIs.

### 3.3 Summary of ICIs Therapies

Combining ICIs with molecularly targeted agents has become a cornerstone of modern oncology. These strategies aim to leverage synergistic mechanisms to overcome drug resistance and boost antitumor immunity. In practice, however, their clinical success hinges on developing next‐generation biomarkers that can refine patient selection and optimize regimens.

Targeted agents do more than block oncogenic pathways like VEGF/VEGFR or EGFR/MAPK; they actively reprogram the immunosuppressive TME. This modulation enhances tumor immunogenicity, effectively “priming” the system for a better ICIs response. The field is now expanding into broader multimodal combinations, incorporating chemotherapy, radiation, and emerging approaches like microbiome modulation. Yet, the rational design of these complex regimens demands more sophisticated tools: dynamic platforms to track temporal tumor–immune interactions and mechanism‐based signatures that match patients to therapies based on dominant resistance pathways. Furthermore, we need computational algorithms capable of truly integrating multiomics data, such as single‐cell RNA‐seq and spatial proteomics, with real‐world evidence to predict synergy. Finally, we cannot overlook the significant economic burden these regimens impose. Future translational research must prioritize precision approaches that maximize therapeutic synergy while limiting toxicity, both physical and financial, to ensure these advances remain accessible in the new era of combination immunotherapy.

## 4 Biomarkers for ICI Therapy

Early research efforts were largely directed toward evaluating single predictive markers, such as PD‐L1 expression, TMB, and microsatellite instability (MSI) [261, 262]. Among these, TMB demonstrated significant predictive value in specific cancer types. For instance, analysis of 151 patients receiving immunotherapy showed that those with higher TMB had significantly better ORRs (58 vs. 20%), PFS (12.8 vs. 3.3 months), and OS (not yet reached vs. 16.3 months) compared with those with lower TMB [263]. A similar trend was observed in 102 patients on PD‐1/PD‐L1 monotherapy, where the median TMB in responders was significantly higher than in nonresponders (18.0 vs. 5.0 mutations/Mb) [263]. This clinical utility led to the US FDA's approval of pembrolizumab for treating tumors with high TMB levels. While high‐MSI tumors typically respond well to PD‐1 blockade due to an abundance of frameshift‐derived neoantigens, TMB and PD‐L1 as standalone biomarkers face inherent biological constraints. TMB indicates the potential for neoantigen generation but does not account for the efficiency of MHC‐mediated presentation, a process governed by the host's germline HLA genotype. Heterozygosity at HLA Class I loci broadens the neoantigen repertoire and correlates with improved survival following ICI therapy [264], whereas somatic loss of heterozygosity at HLA loci can drive intrinsic immune evasion, rendering even high‐TMB tumors therapeutically refractory [265]. TMB also fails to capture clonality: a high burden of subclonal neoantigens, often arising from intratumoral heterogeneity, may favor immune evasion over durable responses. Moreover, the reliability of PD‐L1 expression is compromised by spatial and temporal heterogeneity and a lack of standardized assessment methods, undermining its predictive accuracy [266, 267, 268].

These limitations have spurred a shift from single‐parameter assessments toward integrated, multidimensional approaches that capture the heterogeneity of the TME [269]. Single‐cell and spatial transcriptomics now allow simultaneous profiling of immune cell types, functional states, and their spatial organization within the TME, refining the classification of tumor inflammatory phenotypes. Crucially, responsiveness to PD‐1 blockade hinges not simply on the abundance of progenitor‐exhausted T cells, but on their physical proximity to APCs within specific niches [270, 271, 272], while tumors dominated by terminally exhausted T cells lacking such a microenvironment may exhibit primary resistance. The gut microbiota, as a host factor, has been shown to influence responses to ICIs [273], with certain bacterial genera (such as Faecalibacterium, enriched at baseline) being associated with responders and already utilized in preclinical transplantation studies [274].

Circulating biomarkers are emerging as powerful real‐time complements to tissue‐based assays. For instance, clearance of circulating tumor DNA (ctDNA), expansion of TCR clones, and peripheral T cell activation phenotypes (e.g., CD8^+^ PD‐1^+^ Ki‐67^+^) can capture early signs of treatment response and acquired resistance [275, 276]. These dynamic markers may help distinguish true progression from pseudoprogression, a distinction with significant clinical utility. Meanwhile, multiplex immunohistochemistry combined with multispectral imaging enables quantitative mapping of immune spatial architecture within the TME, moving the field beyond a binary “hot” versus “cold” classification toward a more nuanced framework for guiding personalized combination strategies [277].

Future biomarker development is increasingly shifting from static phenotypic profiling toward high‐throughput functional assessments that probe the biological basis of immune failure. Assessing cellular metabolic competency is one emerging priority: techniques such as single‐cell energetic metabolism by profiling translation inhibition can determine whether T cells harbor sufficient metabolic reserve to support immune activation [278, 279]. Deeper mechanistic insights are provided by chromatin accessibility analyses, like assay for transposase‐accessible chromatin using sequencing (ATAC‐seq), which reveal the epigenetic state and plasticity of immune cells, distinguishing between progenitor and terminally exhausted phenotypes. This distinction is therapeutically vital: terminal exhaustion implies resistance to standard checkpoint blockade, fundamentally shifting the clinical strategy toward epigenetic reprogramming (e.g., HDAC inhibitors) or adoptive cell therapies.

**FIGURE 6:**
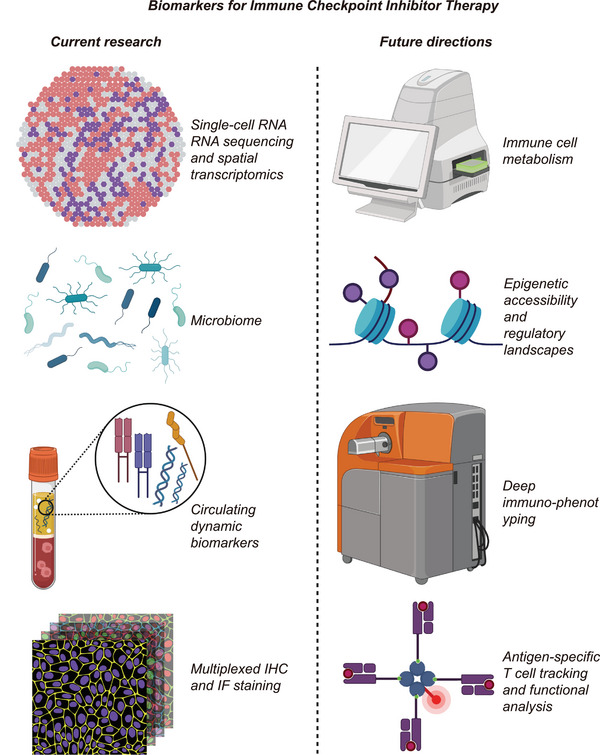
Multidimensional biomarkers and detection strategies for predicting the efficacy of ICI. This figure illustrates various types of biomarkers and their corresponding cutting‐edge technologies used to predict the efficacy of immune checkpoint inhibitors. The key sources of biomarkers listed on the left include single‐cell transcriptomic data from tumor tissues, which reveal the subtypes and states of immune cells; gut microbiota, including Akkermansia muciniphila, Faecalibacterium prausnitzii, and other bacteria associated with therapeutic efficacy; circulating biomarkers such as ctDNA, TCR clonal expansion, and other indicators that reflect dynamic changes in immunity; and spatial immune tissue mapping, which reveals the localization and interactions of immune cells within the tumor microenvironment. The right side displays the corresponding detection platforms and technical methods, including spatial transcriptomics for constructing spatial expression maps; chromatin accessibility techniques such as ATAC‐seq for assessing T cell functional states; CyTOF for high‐dimensional immune phenotyping; and MHC multimer and TCR repertoire analysis for tracking antigen‐specific T cells. These emerging multiomics and functional detection approaches are driving immunotherapy from experience‐driven practices toward precise classification and personalized treatment (created in https://BioRender.com).

This expansion in profiling breadth brings its own challenge. Deep immunophenotyping technologies such as mass cytometry (CyTOF) [280], and antigen‐specific tracking (e.g., MHC multimer staining) generate massive datasets that capture immune subset composition with high resolution [281]. The resulting shift from single‐dimensional assays to multiomics profiling creates a critical bottleneck: data integration. Here, artificial intelligence and machine learning are increasingly used to distill high‐dimensional spatial, genomic, and clinical variables into actionable predictive signatures. Deep learning models applied to routine H&E or multiplexed images can now uncover subtle topological networks of tumor‐stroma interactions that elude human quantification [282, 283]. Ultimately, biomarker development is moving toward systematic immune profiling that integrates tumor, immune, and host information through computational models to advance precision immunotherapy (Figure 6).

## 5 Adverse Reactions of ICIs

The expanding clinical use of ICIs in oncology has been accompanied by growing recognition of their treatment‐related toxicities, termed irAEs. Monotherapy typically manifests as rash, thyroid dysfunction, and mild gastrointestinal symptoms, with severe events being relatively rare [284, 285]. However, the combination of dual ICIs (e.g., PD‐1 + CTLA‐4) significantly increases the incidence and severity of irAEs, with high‐risk reactions such as hepatitis, colitis, and hypophysitis being common [286]. Mechanistically, irAEs are driven by the profound breakdown of immunological self‐tolerance, mediated by the expansion of autoreactive T cell clones, bystander activation, and autoantibody production [287, 288]. The synergistic toxicity of dual blockade stems from the distinct spatial and temporal roles of these checkpoints: CTLA‐4 regulates early T cell priming and Treg function in lymph nodes, whereas PD‐1 suppresses effector T cells in peripheral tissues. Thus, coinhibition comprehensively breaches immune homeostasis.

When ICIs are combined with targeted therapies, the toxicity is markedly additive, with hypertension, liver and kidney damage, and immune‐mediated pneumonitis frequently observed, necessitating careful monitoring of organ function [289]. The combination with OVs demonstrates a favorable overall safety profile, with side effects primarily consisting of local injection reactions and mild flu‐like symptoms [290]. The combination of ICIs with radiotherapy does not significantly increase systemic toxicity but raises the risk of local organ inflammation, such as pneumonia and enteritis [291]. Metal ions (e.g., manganese, iron) may induce oxidative stress and mild inflammatory responses while mobilizing the immune system [255]; however, systemic toxicity still requires clinical evaluation. Interventions targeting gut microbiota (e.g., FMT, probiotics) are generally safe but may modulate the risk of colitis, with certain microbiota even associated with both efficacy and toxicity [292]. Cytokines (e.g., IL‐2, IL‐15) enhance immune activity but also significantly increase the risk of systemic inflammatory responses, including fever, hypotension, and capillary leakage [293]. The toxicity of therapeutic vaccines combined with ICIs is generally mild, primarily manifesting as injection site reactions and transient fever, with no significant increase in severe irAEs [294].

A profound clinical paradox complicates ICI therapies: the occurrence of irAEs frequently correlates with improved and durable antitumor efficacy, suggesting shared immunological pathways, such as cross‐reactivity between tumor neoantigens and healthy tissue antigens. Furthermore, irAEs present diverse clinical trajectories; while mucosal inflammations (e.g., colitis) are typically reversible with intervention, immune‐mediated endocrine toxicities (e.g., hypophysitis, thyroiditis) often culminate in irreversible glandular destruction, condemning patients to lifelong hormone replacement [295, 296]. Conventionally, severe or life‐threatening irAEs are managed with broad immunosuppression, primarily high‐dose corticosteroids, which carries the inherent risk of dampening the desired antitumor CD8^+^ T cell response. To address this, a critical paradigm shift is underway toward targeted molecular therapies designed to dissociate toxicity from efficacy. Emerging evidence suggests that selectively blocking specific inflammatory cytokines that drive irAE pathogenesis, including TNF, IL‐6, IL‐17, or IL‐23, can effectively resolve steroid‐refractory toxicities [297, 298]. Mechanistically, these cytokines predominantly orchestrate collateral tissue damage via myeloid and innate immune cell hyperactivation; neutralizing them quells pathological tissue inflammation while preserving the indispensable, perforin/granzyme‐mediated cytotoxic functions of tumor‐specific CD8^+^ T cells [299, 300, 301].

Ultimately, each combination strategy carries its own toxicity burden—sometimes exacerbating known irAEs, other times inducing entirely new ones. This reality demands that we carefully balance efficacy with safety, tailoring management to each patient's baseline condition. Looking ahead, AI‐driven prediction offers a concrete path to optimize this balance. By integrating diverse patient data that include routine clinical markers, host‐specific factors such as HLA genotypes and baseline autoantibody profiles, and complex multiomics, machine learning models could identify those at highest risk for severe toxicities before treatment begins. Such proactive stratification would allow clinicians to tailor monitoring strategies and prepare preemptive targeted interventions, ensuring that potent antitumor therapy does not come at the cost of unmanageable side effects.

## 6 Emerging Technologies and Future Perspectives in Personalized Immunotherapy

As immunotherapy transitions from the exploratory phase into the era of precision medicine, the integration of technology has become pivotal in enhancing therapeutic efficacy and expanding indications. Currently, artificial intelligence, engineered antibodies, multiomics analysis, and novel biomarker identification are forming the core pillars of next‐generation immunotherapy strategies [302], shifting the paradigm from empirically overcoming resistance to rationally engineering the antitumor response.

### 6.1 AI‐Powered Radiomics and Radiogenomics

The integration of artificial intelligence with radiomics is reshaping the patient screening process in immunotherapy. Traditional tissue biopsies are constrained by profound spatial sampling bias and temporal staticity, often failing to capture the macroscopic heterogeneity of the entire tumor burden. In the study by Jiang et al. [303], the researchers constructed a magnetic resonance imaging radiomics database comprising 860 breast cancer patients, identifying a radiological feature that can distinguish TNBC subtypes and predict recurrence and OS. Notably, they discovered that “peritumoral heterogeneity features” are significantly associated with immunosuppression and abnormal fatty acid metabolism pathways. This research demonstrates that AI‐driven imaging features can not only assist in TNBC subtyping but also serve as noninvasive surrogate markers for the immune microenvironment, providing a novel approach for predicting immunotherapy response [303].

However, a critical gap remains: linking pixel‐level patterns to specific biological pathways is essential to deciphering the black box of AI. The radiological heterogeneity likely reflects underlying physiological chaos, such as disparate vascular permeability and hypoxic gradients, which mechanically exclude T cell infiltration and promote a myeloid‐suppressive phenotype [304, 305]. Establishing a radiogenomic map that correlates specific texture features with gene signatures (e.g., VEGF or TGF‐β signaling) will be key to biologically validating these markers. Beyond these biological challenges, the clinical translation faces a critical barrier: the lack of standardization across imaging protocols and scanners. Overcoming these will require robust, multicenter validated algorithms capable of tolerating data variability to ensure universal applicability.

### 6.2 Bispecific Antibodies and CAR‐T

In the field of engineered antibodies, bispecific antibodies (bsAbs) offer novel mechanisms and therapeutic options for immunotherapy. Represented by glofitamab (CD20×CD3), bsAbs have demonstrated significant efficacy in clinical settings [306]. In a study involving patients with relapsed/refractory diffuse large B cell lymphoma [307], glofitamab achieved an ORR exceeding 65%, with substantial patients achieving a complete response. Its efficacy was also notably evident in patients who had previously failed CAR‐T. Unlike the complex ex vivo manufacturing required for CAR‐T, bsAbs provide immediate availability essential for managing rapidly progressing disease. Moreover, by inducing distinct immunological synapses and utilizing alternative antigen targeting, bsAbs can bypass resistance mechanisms such as antigen loss that restrict CAR‐T efficacy [308]. This achievement demonstrates that bsAbs not only serve as an effective option beyond frontline therapy but also possess the potential to reactivate T cells and restore immune clearance capabilities in patients with drug resistance or relapse.

As the bsAb technology platform matures and multitarget strategies continue to emerge, engineered antibodies will play an increasingly critical role in overcoming tumor resistance, enhancing response depth, and expanding indications, particularly by demonstrating synergistic advantages when combined with traditional ICIs or antibody‐drug conjugates, thereby providing new therapeutic possibilities for complex tumors. However, continuous T cell stimulation by bsAbs can lead to T cell exhaustion and activation‐induced cell death [309, 310]. Addressing this will require optimizing CD3 affinity to balance efficacy with safety, alongside intermittent dosing schedules to preserve long‐term T cell fitness.

### 6.3 Multiomics Integration and Spatial Omics

The deep integration of multiomics is reshaping our understanding of the mechanisms underlying immunotherapy. Recent studies have discovered that CD8^+^ T cells in the tumors of immunotherapy responders exhibited a resident (CD103^+^) phenotype [311], along with specific TCR clonal expansion and expression of IFN response genes. Furthermore, spatial transcriptomics revealed that these T cells penetrated deeply into the core regions of the tumor, forming immunologically active zones in conjunction with APCs. Spatial omics have shifted focus from simple infiltration metrics to the critical role of stromal barriers. Interactions between T cells and stromal components, notably cancer‐associated fibroblasts, establish physical and chemical exclusion zones [312]. Mapping these spatial niches and ligand‐receptor networks is therefore essential for identifying targets to dismantle these barriers. These high‐resolution data are instrumental for predicting responses and guiding tailored combination strategies. Moreover, longitudinal monitoring of these landscapes during treatment will be essential for stratifying responders early in the therapeutic course.

### 6.4 Novel Biomarkers

The field of immunotherapy biomarkers is transitioning from reliance on isolated indicators to an integrated assessment of multidimensional biological profiles. Tumor‐associated tertiary lymphoid structures (TLS) are organized immune cell aggregates that form ectopically within tumor tissues. Emerging evidence indicates that TLS in malignancies such as melanoma and lung cancer exhibit a strong association with improved clinical responses to immune checkpoint blockade therapy [313]. These structures are enriched with mature B cell and T cell zones, which are capable of supporting local immune responses induced by neoantigens. Their gene expression signatures, such as CXCL13, have been validated as biomarkers for favorable prognosis and response, and have been integrated into various immune scoring systems. In our prior work, we validated the presence of TLS in renal cancer [314], and deciphered the implications of the heterogeneous pattern of TLS in renal cancer. Our results revealed that most TLS in renal cancer were located in the tumor‐distal area and presented immature features, contributing to shaping the immunosuppressive TME [315].

Recent research highlights a critical distinction in TLS based on their location and maturity. Peritumoral TLS were predominantly in an early developmental stage, often appearing as primary follicles. These clusters also showed elevated infiltration of TAMs and Tregs, suggesting they contribute to an immunosuppressive microenvironment. In contrast, intratumoral and intertumoral TLS mainly consisted of mature, secondary follicle‐like TLS (SFL‐TLS), which are characterized by active germinal centers [316]. These mature structures are strongly linked to enhanced patient survival. Specifically, both intertumoral TLS and SFL‐TLS were associated with prolonged survival and higher ORRs in RCC patients undergoing PD‐1/PD‐L1 blockade [317]. Mechanistically, these mature TLS were enriched with plasma cells capable of secreting IgA and IgG, further supporting their positive role in the immunotherapy response [318]. Beyond antibody production, B cells within mature TLS serve as potent APCs, presenting neoantigens to CD4^+^ and CD8^+^ T cells [319]. This process facilitates epitope spreading, reinforcing the cancer‐immunity cycle and promoting durable responses. Overall, the spatial distribution and maturation status of TLS significantly shape the immune microenvironment and clinical outcomes in clear cell RCC, potentially offering a new basis for precise immunotherapy [313]. The dichotomy between mature and immature phenotypes supports targeting TLS not just as prognostic markers, but as therapeutic entities [315, 320]. Current efforts focus on inducing functional, mature TLS in “immune‐cold” tumors. Approaches using STING agonists or TLS‐promoting cytokines (e.g., CXCL13, LTβ) aim to drive the neogenesis of these lymphoid niches, thereby sensitizing resistant tumors to checkpoint blockade [321, 322].

In summary, AI‐assisted diagnosis, engineered antibody design, integrated biomics, and precision biomarker discovery are collectively advancing immunotherapy from “generalized medication” to “individualized customization.” Future immune strategies will place greater emphasis on dynamic regulation, multimodal collaboration, and predictive decision‐making, driving breakthroughs in immunotherapy across a wider array of tumor types (Figure 6).

## 7 Conclusion and Future Perspectives

Immune checkpoint therapy revolutionized oncology in its first decade, but has since hit a plateau. Low response rates and pervasive resistance remain stubbornly unresolved. The explosion of combination strategies, which pair checkpoint inhibitors with everything from chemotherapy to microbiome modulators, reflects a field scrambling to break the stalemate. However, these combinations offer only marginal benefits at the cost of significant added toxicity. The field is shifting from the brute‐force era of broad inhibition to a more refined paradigm grounded in precision and mechanistic synergy. The next transformative leap will not be a singular magic bullet, but rather the integration of three interconnected principles:

### 7.1 Prioritizing Mechanistic Synergy Over Empirical Combinations

The era of “try everything with PD‐1” is nearing its end. Future combinations must be grounded in clear mechanistic logic, both for action and resistance. Chemotherapy, for example, reshapes the immune environment beyond just inducing cell death [178]. Similarly, anti‐VEGF agents enhance T cell infiltration via vascular normalization [198]. The critical challenge has shifted from identifying candidate combinations to establishing mechanistically justified and temporally optimized strategies, grounded in an understanding of both therapeutic action and resistance pathways.

### 7.2 Advancing From Static Biomarkers to Dynamic Immune Monitoring

As mentioned above, static biomarkers like baseline PD‐L1 expression or TMB offer limited predictive insight. The immune response to cancer is a dynamic process, necessitating a shift toward real‐time monitoring. Approaches such as tracking the kinetics of ctDNA clearance, monitoring the expansion of the TCR repertoire, or employing validated radiomic signatures could enable adaptive therapeutic strategies. Ultimately, such biomarkers enable a transition from static treatment paradigms to adaptive, mechanism‑informed modulation of therapeutic intensity, allowing real‑time alignment between biological response dynamics and clinical decision‑making.

### 7.3 Integrating a Holistic “Host–TME” Perspective

The historical focus of oncology has been predominantly tumor‐intrinsic. However, growing evidence underscores the critical importance of the host's biology and the TME. Insights into the influence of the gut microbiota, host systemic metabolism, and the structural architecture of the TME, such as the presence and maturity of TLS, collectively demonstrate that the host's physiological state and the immunological context of the tumor are fundamental determinants of therapeutic outcome. A patient's unique microbiome, metabolic profile, and the spatial organization of immune cells within the TME establish the necessary conditions for a successful response to checkpoint blockade.

Therefore, the critical next phase is not merely to adopt these concepts, but to execute them with precision. The central challenge now is one of integration: weaving together multiomics data streams, AI‐powered causal inference, and dynamic monitoring into a seamless clinical feedback loop. This will elevate combination therapy from an empirical arsenal into a predictive, adaptive system. The ultimate goal is a new paradigm of precision immune engineering, where treatment is continuously tailored to the evolving biology of the patient, finally overcoming the pervasive challenge of resistance.

## Author Contributions

*Conceptualization*: Qintao Ge, Liu Yu, Jiahe Lu, Aihetaimujiang Anwaier, and Wenhao Xu. *Methodology*: Qintao Ge, Liu Yu, Jiahe Lu, Aihetaimujiang Anwaier, and Xiyue Xiao. *Formal analysis*: Qintao Ge, Liu Yu, Jiahe Lu, and Yonghao Chen. *Investigation*: Qintao Ge and Wenhao Xu. *Writing the original draft*: Qintao Ge, Wenhao Xu, Liu Yu, and Jiahe Lu. *Visualization*: Qintao Ge, Liu Yu, and Jiahe Lu. *Funding acquisition*: Dingwei Ye, Hailiang Zhang, Wenhao Xu, and Wenbin Dai. *Supervision*: Dingwei Ye, Wenhao Xu, Hailiang Zhang, and Wenbin Dai. All authors have read and approved the final manuscript.

## Funding

This work was supported by grants from the Noncommunicable Chronic Diseases‐National Science and Technology Major Project (No. 2023ZD0510300), the National Natural Science Foundation of China (No. 82403377, No. 82474506), the China Postdoctoral Science Foundation (No. GZC20230500, 2024M750538), and the Natural Science Foundation of Shanghai (No. 25ZR1401067).

## Ethics Statement

The authors have nothing to report.

## Conflicts of Interest

The authors declare no conflicts of interest.

## Data Availability

The raw data for this study were generated at the corresponding archives. Further inquiries can be directed to the corresponding authors upon reasonable request.

## References

[1] P. S. Adusumilli , E. Cha , M. Cornfeld , et al., “New Cancer Immunotherapy Agents in Development: A Report From an Associated Program of the 31stAnnual Meeting of the Society for Immunotherapy of Cancer, 2016,” Journal for Immunotherapy of Cancer 5 (2017): 50. 28649381 10.1186/s40425-017-0253-2 PMC5477277

[2] Y. Xu , Z. Xiang , M. Alnaggar , et al., “Allogeneic Vγ9Vδ2 T‐Cell Immunotherapy Exhibits Promising Clinical Safety and Prolongs the Survival of Patients With Late‐Stage Lung or Liver Cancer,” Cellular & Molecular Immunology 18, no. 2 (2021): 427–439. 32939032 10.1038/s41423-020-0515-7 PMC8027668

[3] Y. Ishida , Y. Agata , K. Shibahara , and T. Honjo , “Induced Expression of PD‐1, a Novel Member of the Immunoglobulin Gene Superfamily, Upon Programmed Cell Death,” The EMBO Journal 11, no. 11 (1992): 3887–3895. 1396582 10.1002/j.1460-2075.1992.tb05481.x PMC556898

[4] F. S. Hodi , S. J. O'Day , D. F. McDermott , et al., “Improved Survival With Ipilimumab in Patients With Metastatic Melanoma,” The New England Journal of Medicine 363, no. 8 (2010): 711–723. 20525992 10.1056/NEJMoa1003466 PMC3549297

[5] S. Arora , S. Balasubramaniam , W. Zhang , et al., “FDA Approval Summary: Pembrolizumab Plus Lenvatinib for Endometrial Carcinoma, a Collaborative International Review Under Project Orbis,” Clinical Cancer Research: An Official Journal of the American Association for Cancer Research 26, no. 19 (2020): 5062–5067. 32295834 10.1158/1078-0432.CCR-19-3979

[6] J. Paik , “Nivolumab Plus Relatlimab: First Approval,” Drugs 82, no. 8 (2022): 925–931. 35543970 10.1007/s40265-022-01723-1

[7] P. Schmid , J. Cortes , L. Pusztai , et al., “Pembrolizumab for Early Triple‐Negative Breast Cancer,” The New England Journal of Medicine 382, no. 9 (2020): 810–821. 32101663 10.1056/NEJMoa1910549

[8] J. Cortes , H. S. Rugo , D. W. Cescon , et al., “Pembrolizumab Plus Chemotherapy in Advanced Triple‐Negative Breast Cancer,” The New England Journal of Medicine 387, no. 3 (2022): 217–226. 35857659 10.1056/NEJMoa2202809

[9] P. Schmid , J. Cortes , R. Dent , et al., “Event‐Free Survival With Pembrolizumab in Early Triple‐Negative Breast Cancer,” The New England Journal of Medicine 386, no. 6 (2022): 556–567. 35139274 10.1056/NEJMoa2112651

[10] M. Jin , J. Fang , J. Peng , et al., “PD‐1/PD‐L1 Immune Checkpoint Blockade in Breast Cancer: Research Insights and Sensitization Strategies,” Molecular Cancer 23 (2024): 266. 39614285 10.1186/s12943-024-02176-8 PMC11605969

[11] M. Vesely , T. Zhang , and L. Chen , “Resistance Mechanisms to Anti‐PD Cancer Immunotherapy,” Annual Review of Immunology 40 (2022): 45–74. 10.1146/annurev-immunol-070621-030155 35471840

[12] T. Li , C. Zhang , G. Zhao , et al., “IGFBP2 Regulates PD‐L1 Expression by Activating the EGFR‐STAT3 Signaling Pathway in Malignant Melanoma,” Cancer Letters 477 (2020): 19–30. 32120023 10.1016/j.canlet.2020.02.036 PMC7816098

[13] X. Xiao , J. Shi , C. He , et al., “ERK and USP5 Govern PD‐1 Homeostasis via Deubiquitination to Modulate Tumor Immunotherapy,” Nature Communications 14, no. 1 (2023): 2859. 10.1038/s41467-023-38605-3 PMC10199079 37208329

[14] K. Gao , Q. Shi , Y. Gu , et al., “SPOP Mutations Promote Tumor Immune Escape in Endometrial Cancer via the IRF1‐PD‐L1 Axis,” Cell Death and Differentiation 30, no. 2 (2023): 475–487. 36481790 10.1038/s41418-022-01097-7 PMC9950446

[15] T. André , E. Elez , H. J. Lenz , et al., “Nivolumab Plus Ipilimumab Versus Nivolumab in Microsatellite Instability‐High Metastatic Colorectal Cancer (CheckMate 8HW): A Randomised, Open‐Label, Phase 3 Trial,” Lancet 405, no. 10476 (2025): 383–395. 39874977 10.1016/S0140-6736(24)02848-4

[16] J. Chen , Z. Amoozgar , X. Liu , et al., “Reprogramming the Intrahepatic Cholangiocarcinoma Immune Microenvironment by Chemotherapy and CTLA‐4 Blockade Enhances Anti‐PD‐1 Therapy,” Cancer Immunology Research 12, no. 4 (2024): 400–412. 38260999 10.1158/2326-6066.CIR-23-0486 PMC10985468

[17] T. Yokosuka , M. Takamatsu , W. Kobayashi‐Imanishi , A. Hashimoto‐Tane , M. Azuma , and T. Saito , “Programmed Cell Death 1 Forms Negative Costimulatory Microclusters That Directly Inhibit T Cell Receptor Signaling by Recruiting Phosphatase SHP2,” Journal of Experimental Medicine 209, no. 6 (2012): 1201–1217. 22641383 10.1084/jem.20112741 PMC3371732

[18] X. Zhang , J. D. Schwartz , X. Guo , et al., “Structural and Functional Analysis of the Costimulatory Receptor Programmed Death‐1,” Immunity 20, no. 3 (2004): 337–347. 15030777 10.1016/s1074-7613(04)00051-2

[19] E. A. Philips , J. Liu , A. Kvalvaag , et al., “Transmembrane Domain‐Driven PD‐1 Dimers Mediate T Cell Inhibition,” Science Immunology 9, no. 93 (2024): eade6256. 38457513 10.1126/sciimmunol.ade6256 PMC11166110

[20] E. Hui , J. Cheung , J. Zhu , et al., “T Cell Costimulatory Receptor CD28 Is a Primary Target for PD‐1‐Mediated Inhibition,” Science 355, no. 6332 (2017): 1428–1433. 28280247 10.1126/science.aaf1292 PMC6286077

[21] K. Hofmeyer , H. Jeon , and X. Zang , “The PD‐1/PD‐L1 (B7‐H1) Pathway in Chronic Infection‐Induced Cytotoxic T Lymphocyte Exhaustion,” Journal of Biomedicine and Biotechnology 2011 (2011): 451694. 21960736 10.1155/2011/451694 PMC3180079

[22] W. Piao , L. Li , V. Saxena , et al., “PD‐L1 Signaling Selectively Regulates T Cell Lymphatic Transendothelial Migration,” Nature Communications 13, no. 1 (2022): 2176. 10.1038/s41467-022-29930-0 PMC9023578 35449134

[23] A. Salmaninejad , S. F. Valilou , A. G. Shabgah , et al., “PD‐1/PD‐L1 Pathway: Basic Biology and Role in Cancer Immunotherapy,” Journal of Cellular Physiology 234, no. 10 (2019): 16824–16837. 30784085 10.1002/jcp.28358

[24] J. A. Turner , E. Stephen‐Victor , S. Wang , et al., “Regulatory T Cell‐Derived TGF‐β1 Controls Multiple Checkpoints Governing Allergy and Autoimmunity,” Immunity 53, no. 6 (2020): 1202–1214.e6. 33086036 10.1016/j.immuni.2020.10.002 PMC7744401

[25] L. M. Francisco , V. H. Salinas , K. E. Brown , et al., “PD‐L1 Regulates the Development, Maintenance, and Function of Induced Regulatory T Cells,” Journal of Experimental Medicine 206, no. 13 (2009): 3015–3029. 20008522 10.1084/jem.20090847 PMC2806460

[26] S. R. Gordon , R. L. Maute , B. W. Dulken , et al., “PD‐1 Expression by Tumour‐Associated Macrophages Inhibits Phagocytosis and Tumour Immunity,” Nature 545, no. 7655 (2017): 495–499. 28514441 10.1038/nature22396 PMC5931375

[27] S. Sauer , L. Bruno , A. Hertweck , et al., “T Cell Receptor Signaling Controls Foxp3 Expression via PI3K, Akt, and mTOR,” Proceedings of the National Academy of Sciences of the United States of America 105, no. 22 (2008): 7797–7802. 18509048 10.1073/pnas.0800928105 PMC2409380

[28] Y. Hu and C. Liu , “SIRT3‐SUMO Regulated Treg Cell Differentiation and Asthma Development by Mediating N‐Glycosylation Through the FAO Pathway,” Cell Biology and Toxicology 41, no. 1 (2025): 164. 41388161 10.1007/s10565-025-10105-8 PMC12700947

[29] C. McDonald‐Hyman , E. G. Aguilar , E. B. Compeer , et al., “Acetyl‐CoA Carboxylase 1 Inhibition Increases Treg Metabolism and Graft‐Versus‐Host Disease Treatment Efficacy via Mitochondrial Fusion,” Journal of Clinical Investigation 135, no. 23 (2025): e182480. 41026528 10.1172/JCI182480 PMC12646659

[30] Z. Chi , Y. Lu , Y. Yang , B. Li , and P. Lu , “Transcriptional and Epigenetic Regulation of PD‐1 Expression,” Cellular and Molecular Life Sciences 78, no. 7 (2021): 3239–3246. 33738533 10.1007/s00018-020-03737-y PMC11073161

[31] A. Bally , J. Austin , and J. Boss , “Genetic and Epigenetic Regulation of PD‐1 Expression,” Journal of Immunology 196, no. 6 (2016): 2431–2437. 10.4049/jimmunol.1502643 PMC4780223 26945088

[32] K. J. Oestreich , H. Yoon , R. Ahmed , and J. M. Boss , “NFATc1 Regulates PD‐1 Expression Upon T Cell Activation,” Journal of Immunology 181, no. 7 (2008): 4832–4839. 10.4049/jimmunol.181.7.4832 PMC2645436 18802087

[33] J. W. Austin , P. Lu , P. Majumder , R. Ahmed , and J. M. Boss , “STAT3, STAT4, NFATc1, and CTCF Regulate PD‐1 Through Multiple Novel Regulatory Regions in Murine T Cells,” Journal of Immunology 192, no. 10 (2014): 4876–4886. 10.4049/jimmunol.1302750 PMC4011967 24711622

[34] S. Terawaki , S. Chikuma , S. Shibayama , et al., “IFN‐α Directly Promotes Programmed Cell Death‐1 Transcription and Limits the Duration of T Cell‐Mediated Immunity,” Journal of Immunology 186, no. 5 (2011): 2772–2779. 10.4049/jimmunol.1003208 21263073

[35] P. Lu , B. A. Youngblood , J. W. Austin , et al., “Blimp‐1 Represses CD8 T Cell Expression of PD‐1 Using a Feed‐Forward Transcriptional Circuit During Acute Viral Infection,” Journal of Experimental Medicine 211, no. 3 (2014): 515–527. 24590765 10.1084/jem.20130208 PMC3949569

[36] C. Kao , K. J. Oestreich , M. A. Paley , et al., “Transcription Factor T‐Bet Represses Expression of the Inhibitory Receptor PD‐1 and Sustains Virus‐Specific CD8+ T Cell Responses During Chronic Infection,” Nature Immunology 12, no. 7 (2011): 663–671. 21623380 10.1038/ni.2046 PMC3306165

[37] B. Youngblood , A. Noto , F. Porichis , et al., “Cutting Edge: Prolonged Exposure to HIV Reinforces a Poised Epigenetic Program for PD‐1 Expression in Virus‐Specific CD8 T Cells,” Journal of Immunology 191, no. 2 (2013): 540–544. 10.4049/jimmunol.1203161 PMC3702641 23772031

[38] X. Meng , X. Liu , X. Guo , et al., “FBXO38 Mediates PD‐1 Ubiquitination and Regulates Anti‐Tumour Immunity of T Cells,” Nature 564, no. 7734 (2018): 130–135. 30487606 10.1038/s41586-018-0756-0

[39] N. Zhang , M. Li , X. Xu , et al., “Loss of Core Fucosylation Enhances the Anticancer Activity of Cytotoxic T Lymphocytes by Increasing PD‐1 Degradation,” European Journal of Immunology 50, no. 11 (2020): 1820–1833. 32460355 10.1002/eji.202048543

[40] M. Okada , S. Chikuma , T. Kondo , et al., “Blockage of Core Fucosylation Reduces Cell‐Surface Expression of PD‐1 and Promotes Anti‐Tumor Immune Responses of T Cells,” Cell Reports 20, no. 5 (2017): 1017–1028. 28768188 10.1016/j.celrep.2017.07.027

[41] X. Wang , Q. He , H. Shen , et al., “TOX Promotes the Exhaustion of Antitumor CD8(+) T Cells by Preventing PD1 Degradation in Hepatocellular Carcinoma,” Journal of Hepatology 71, no. 4 (2019): 731–741. 31173813 10.1016/j.jhep.2019.05.015

[42] M. E. Keir , M. J. Butte , G. J. Freeman , and A. H. Sharpe , “PD‐1 and Its Ligands in Tolerance and Immunity,” Annual Review of Immunology 26 (2008): 677–704. 10.1146/annurev.immunol.26.021607.090331 PMC10637733 18173375

[43] D. Y. Lin , Y. Tanaka , M. Iwasaki , et al., “The PD‐1/PD‐L1 Complex Resembles the Antigen‐Binding Fv Domains of Antibodies and T Cell Receptors,” Proceedings of the National Academy of Sciences of the United States of America 105, no. 8 (2008): 3011–3016. 18287011 10.1073/pnas.0712278105 PMC2268576

[44] A. V. Kornepati , J. T. Boyd , C. E. Murray , et al., “Tumor Intrinsic PD‐L1 Promotes DNA Repair in Distinct Cancers and Suppresses PARP Inhibitor‐Induced Synthetic Lethality,” Cancer Research 82, no. 11 (2022): 2156–2170. 35247877 10.1158/0008-5472.CAN-21-2076 PMC9987177

[45] X. He and C. Xu , “Immune Checkpoint Signaling and Cancer Immunotherapy,” Cell Research 30, no. 8 (2020): 660–669. 32467592 10.1038/s41422-020-0343-4 PMC7395714

[46] A. Chaudhri , Y. Xiao , A. N. Klee , X. Wang , B. Zhu , and G. J. Freeman , “PD‐L1 Binds to B7‐1 Only in Cis on the Same Cell Surface,” Cancer Immunology Research 6, no. 8 (2018): 921–929. 29871885 10.1158/2326-6066.CIR-17-0316 PMC7394266

[47] D. Sugiura , T. Maruhashi , I. Okazaki , et al., “Restriction of PD‐1 Function by Cis‐PD‐L1/CD80 Interactions Is Required for Optimal T Cell Responses,” Science 364, no. 6440 (2019): 558–566. 31000591 10.1126/science.aav7062

[48] Y. Zhang , Q. Song , K. Cassady , et al., “Blockade of Trans PD‐L1 Interaction With CD80 Augments Antitumor Immunity,” Proceedings of the National Academy of Sciences of the United States of America 120, no. 16 (2023): e2205085120. 37036990 10.1073/pnas.2205085120 PMC10120074

[49] Y. Liu , Z. Yang , S. Wang , et al., “Nuclear PD‐L1 Compartmentalization Suppresses Tumorigenesis and Overcomes Immunocheckpoint Therapy Resistance in Mice via Histone macroH2A1,” Journal of Clinical Investigation 134, no. 22 (2024): e181314. 39545415 10.1172/JCI181314 PMC11563670

[50] Y. Wang , Y. Zhou , L. Yang , et al., “Challenges Coexist With Opportunities: Spatial Heterogeneity Expression of PD‐L1 in Cancer Therapy,” Advanced Science (Weinheim, Baden‐Wurttemberg, Germany) 11, no. 1 (2024): e2303175. 37934012 10.1002/advs.202303175 PMC10767451

[51] H. Wang , H. Yao , C. Li , et al., “HIP1R Targets PD‐L1 to Lysosomal Degradation to Alter T Cell‐Mediated Cytotoxicity,” Nature Chemical Biology 15, no. 1 (2019): 42–50. 30397328 10.1038/s41589-018-0161-x

[52] A. Satelli , I. S. Batth , Z. Brownlee , et al., “Potential Role of Nuclear PD‐L1 Expression in Cell‐Surface Vimentin Positive Circulating Tumor Cells as a Prognostic Marker in Cancer Patients,” Scientific Reports 6 (2016): 28910. 27363678 10.1038/srep28910 PMC4929464

[53] W. Du , J. Zhu , Y. Zeng , et al., “KPNB1‐Mediated Nuclear Translocation of PD‐L1 Promotes Non‐Small Cell Lung Cancer Cell Proliferation via the Gas6/MerTK Signaling Pathway,” Cell Death and Differentiation 28, no. 4 (2021): 1284–1300. 33139930 10.1038/s41418-020-00651-5 PMC8027631

[54] J. Yu , A. Zhuang , X. Gu , et al., “Nuclear PD‐L1 Promotes EGR1‐Mediated Angiogenesis and Accelerates Tumorigenesis,” Cell Discovery 9, no. 1 (2023): 33. 36977660 10.1038/s41421-023-00521-7 PMC10050073

[55] M. Theodoraki , S. S. Yerneni , T. K. Hoffmann , W. E. Gooding , and T. L. Whiteside , “Clinical Significance of PD‐L1(+) Exosomes in Plasma of Head and Neck Cancer Patients,” Clinical Cancer Research 24, no. 4 (2018): 896–905. 29233903 10.1158/1078-0432.CCR-17-2664 PMC6126905

[56] M. Poggio , T. Hu , C. Pai , et al., “Suppression of Exosomal PD‐L1 Induces Systemic Anti‐Tumor Immunity and Memory,” Cell 177, no. 2 (2019): 414–427.e13. 30951669 10.1016/j.cell.2019.02.016 PMC6499401

[57] J. Yang , J. Chen , H. Liang , and Y. Yu , “Nasopharyngeal Cancer Cell‐Derived Exosomal PD‐L1 Inhibits CD8+ T‐Cell Activity and Promotes Immune Escape,” Cancer Science 113, no. 9 (2022): 3044–3054. 35598173 10.1111/cas.15433 PMC9459270

[58] G. Chen , A. C. Huang , W. Zhang , et al., “Exosomal PD‐L1 Contributes to Immunosuppression and Is Associated With Anti‐PD‐1 Response,” Nature 560, no. 7718 (2018): 382–386. 30089911 10.1038/s41586-018-0392-8 PMC6095740

[59] F. Ma , X. Liu , Y. Zhang , et al., “Tumor Extracellular Vesicle‐Derived PD‐L1 Promotes T Cell Senescence Through Lipid Metabolism Reprogramming,” Science Translational Medicine 17, no. 785 (2025): eadm7269. 39937879 10.1126/scitranslmed.adm7269 PMC12063564

[60] I. Zerdes , A. Matikas , J. Bergh , G. Z. Rassidakis , and T. Foukakis , “Genetic, Transcriptional and Post‐Translational Regulation of the Programmed Death Protein Ligand 1 in Cancer: Biology and Clinical Correlations,” Oncogene 37, no. 34 (2018): 4639–4661. 29765155 10.1038/s41388-018-0303-3 PMC6107481

[61] B. Liu , Y. Wu , A. Xiang , et al., “The Hexosamine Biosynthetic Pathway Drives Tumor Immune Evasion via Translational Control of PD‐L1 at the Elongation Level,” Cell Reports 44, no. 9 (2025): 116249. 40938752 10.1016/j.celrep.2025.116249

[62] P. Srivastava , M. Rütter , G. M. Antoniraj , Y. Ventura , and A. David , “Mesoporous Silica Nanoparticles With Extra‐Large Pores for Boosting Antigen Presentation and PD‐L1 Immune Checkpoint Blockade,” ACS Applied Bio Materials 8, no. 11 (2025): 9864–9874. 10.1021/acsabm.5c01211 41166685

[63] Y. Li , J. Zhu , F. Zhai , et al., “LMNB2‐Mediated High PD‐L1 Transcription Triggers the Immune Escape of Hepatocellular Carcinoma,” Cell Death Discovery 11, no. 1 (2025): 269. 40483310 10.1038/s41420-025-02540-7 PMC12145441

[64] X. Wang , X. Yang , L. Xing , et al., “CircLRBA Promotes Epithelial‐Mesenchymal Transition, Immune Evasion, Chemoimmunotherapy Resistance and Metastasis Through Stabilizing Twist1,” Advanced science (Weinheim, Baden‐Wurttemberg, Germany) 12, no. 48 (2025): e08918. 41082269 10.1002/advs.202508918 PMC12752550

[65] S. Liu , Y. Pan , W. Liu , et al., “Lactylation‐Driven MVP Upregulation Boosts Immunotherapy Resistance by Inhibiting PD‐L1 Degradation in Hepatocellular Carcinoma,” Journal for ImmunoTherapy of Cancer 13, no. 9 (2025): e012230. 40983342 10.1136/jitc-2025-012230 PMC12458742

[66] T. Iida , H. Ohno , C. Nakaseko , et al., “Regulation of Cell Surface Expression of CTLA‐4 by Secretion of CTLA‐4‐Containing Lysosomes Upon Activation of CD4+ T Cells,” Journal of Immunology 165, no. 9 (2000): 5062–5068. 10.4049/jimmunol.165.9.5062 11046036

[67] E. Valk , C. Rudd , and H. Schneider , “CTLA‐4 Trafficking and Surface Expression,” Trends in Immunology 29, no. 6 (2008): 272–279. 18468488 10.1016/j.it.2008.02.011 PMC4186961

[68] D. Oyewole‐Said , V. Konduri , J. Vazquez‐Perez , S. A. Weldon , J. M. Levitt , and W. K. Decker , “Beyond T‐Cells: Functional Characterization of CTLA‐4 Expression in Immune and Non‐Immune Cell Types,” Frontiers in Immunology 11 (2020): 608024. 33384695 10.3389/fimmu.2020.608024 PMC7770141

[69] M. M. Muthana , X. Du , M. Liu , et al., “CTLA‐4 Antibody‐Drug Conjugate Reveals Autologous Destruction of B‐Lymphocytes Associated With Regulatory T Cell Impairment,” Elife 12 (2023): RP87281. 38127423 10.7554/eLife.87281 PMC10735222

[70] P. Do , K. A. Beckwith , C. Cheney , et al., “Leukemic B Cell CTLA‐4 Suppresses Costimulation of T Cells,” Journal of Immunology 202, no. 9 (2019): 2806–2816. 10.4049/jimmunol.1801359 PMC6478536 30910862

[71] D. Quandt , H. Hoff , M. Rudolph , S. Fillatreau , and M. C. Brunner‐Weinzierl , “A New Role of CTLA‐4 on B Cells in Thymus‐Dependent Immune Responses in Vivo,” Journal of Immunology 179, no. 11 (2007): 7316–7324. 10.4049/jimmunol.179.11.7316 18025174

[72] Y. Chen and H. Shi , “CD28/CTLA‐4–CD80/CD86 and ICOS–B7RP‐1 Costimulatory Pathway in Bronchial Asthma,” Allergy 61, no. 1 (2006): 15–26. 10.1111/j.1398-9995.2006.01008.x 16364152

[73] W. Teft , M. Kirchhof , and J. Madrenas , “A Molecular Perspective of CTLA‐4 Function,” Annual Review of Immunology 24 (2006): 65–97. 10.1146/annurev.immunol.24.021605.090535 16551244

[74] K. Chattopadhyay , E. Lazar‐Molnar , Q. Yan , et al., “Sequence, Structure, Function, Immunity: Structural Genomics of Costimulation,” Immunological Reviews 229, no. 1 (2009): 356–386. 19426233 10.1111/j.1600-065X.2009.00778.x PMC3001128

[75] J. Egen , M. Kuhns , and J. Allison , “CTLA‐4: New Insights Into Its Biological Function and Use in Tumor Immunotherapy,” Nature Immunology 3, no. 7 (2002): 611–618. 12087419 10.1038/ni0702-611

[76] K. Wing , Y. Onishi , P. Prieto‐Martin , et al., “CTLA‐4 Control Over Foxp3+ Regulatory T Cell Function,” Science 322, no. 5899 (2008): 271–275. 18845758 10.1126/science.1160062

[77] P. S. Linsley , J. L. Greene , W. Brady , J. Bajorath , J. A. Ledbetter , and R. Peach , “Human B7‐1 (CD80) and B7‐2 (CD86) Bind With Similar Avidities but Distinct Kinetics to CD28 and CTLA‐4 Receptors,” Immunity 1, no. 9 (1994): 793–801. 7534620 10.1016/s1074-7613(94)80021-9

[78] J. L. Greene , G. M. Leytze , J. Emswiler , et al., “Covalent Dimerization of CD28/CTLA‐4 and Oligomerization of CD80/CD86 Regulate T Cell Costimulatory Interactions,” Journal of Biological Chemistry 271, no. 43 (1996): 26762–26771. 8900156 10.1074/jbc.271.43.26762

[79] A. Kennedy , E. Waters , B. Rowshanravan , et al., “Differences in CD80 and CD86 Transendocytosis Reveal CD86 as a Key Target for CTLA‐4 Immune Regulation,” Nature Immunology 23, no. 9 (2022): 1365–1378. 35999394 10.1038/s41590-022-01289-w PMC9477731

[80] F. Fallarino , U. Grohmann , K. W. Hwang , et al., “Modulation of Tryptophan Catabolism by Regulatory T Cells,” Nature Immunology 4, no. 12 (2003): 1206–1212. 14578884 10.1038/ni1003

[81] D. H. Munn , E. Shafizadeh , J. T. Attwood , I. Bondarev , A. Pashine , and A. L. Mellor , “Inhibition of T Cell Proliferation by Macrophage Tryptophan Catabolism,” Journal of Experimental Medicine 189, no. 9 (1999): 1363–1372. 10224276 10.1084/jem.189.9.1363 PMC2193062

[82] U. Grohmann , “Tolerance, DCs and Tryptophan: Much Ado About IDO,” Trends in Immunology 24, no. 5 (2003): 242–248. 12738417 10.1016/s1471-4906(03)00072-3

[83] U. Grohmann , C. Orabona , F. Fallarino , et al., “CTLA‐4‐Ig Regulates Tryptophan Catabolism in Vivo,” Nature Immunology 3, no. 11 (2002): 1097–1101. 12368911 10.1038/ni846

[84] F. Fallarino , U. Grohmann , C. Vacca , et al., “T Cell Apoptosis by Tryptophan Catabolism,” Cell Death and Differentiation 9, no. 10 (2002): 1069–1077. 12232795 10.1038/sj.cdd.4401073

[85] M. K. Oaks , K. M. Hallett , R. Penwell , E. C. Stauber , S. J. Warren , and A. J. Tector , “A Native Soluble Form of CTLA‐4,” Cellular Immunology 201, no. 2 (2000): 144–153. 10831323 10.1006/cimm.2000.1649

[86] F. J. Ward , L. N. Dahal , S. K. Wijesekera , et al., “The Soluble Isoform of CTLA‐4 as a Regulator of T‐Cell Responses,” European Journal of Immunology 43, no. 5 (2013): 1274–1285. 23400950 10.1002/eji.201242529

[87] V. Kalia , L. Penny , Y. Yuzefpolskiy , F. Baumann , and S. Sarkar , “Quiescence of Memory CD8(+) T Cells Is Mediated by Regulatory T Cells Through Inhibitory Receptor CTLA‐4,” Immunity 42, no. 6 (2015): 1116–1129. 26084026 10.1016/j.immuni.2015.05.023

[88] R. Zappasodi , I. Serganova , I. J. Cohen , et al., “CTLA‐4 Blockade Drives Loss of T(reg) Stability in Glycolysis‐Low Tumours,” Nature 591, no. 7851 (2021): 652–658. 33588426 10.1038/s41586-021-03326-4 PMC8057670

[89] P. Sage , A. Paterson , S. Lovitch , and A. Sharpe , “The Coinhibitory Receptor CTLA‐4 Controls B Cell Responses by Modulating T Follicular Helper, T Follicular Regulatory, and T Regulatory Cells,” Immunity 41, no. 6 (2014): 1026–1039. 25526313 10.1016/j.immuni.2014.12.005 PMC4309019

[90] X. Chen , M. Yang , Y. Huang , J. Tu , Y. Cai , and X. Yuan , “Molecular Mechanisms Underlying the Abscopal Effect Induced by Radiotherapy and Its Synergistic Translational Potential With Immunotherapy,” Therapeutic Advances in Medical Oncology 17 (2025): 17588359251387534. 41179116 10.1177/17588359251387534 PMC12579151

[91] V. Szentgyörgyi , L. M. Lueck , D. Overwijn , et al., “Arf1‐Dependent LRBA Recruitment to Rab4 Endosomes is Required for Endolysosome Homeostasis,” Journal of Cell Biology 223, no. 11 (2024): e202401167. 39325073 10.1083/jcb.202401167 PMC11449124

[92] M. Zhang , J. Li , K. Yan , et al., “pH‐Dependent Dissociation From CTLA‐4 in Early Endosomes Improves Both Safety and Antitumor Activity of Anti‐CTLA‐4 Antibodies,” Proceedings of the National Academy of Sciences of the United States of America 122, no. 8 (2025): e2422731122. 39964714 10.1073/pnas.2422731122 PMC11874271

[93] X. Wang , J. He , G. Ding , Y. Tang , and Q. Wang , “Overcoming Resistance to PD‐1 and CTLA‐4 Blockade Mechanisms and Therapeutic Strategies,” Frontiers in Immunology 16 (2025): 1688699. 41112253 10.3389/fimmu.2025.1688699 PMC12531160

[94] X. Tai , F. Van Laethem , L. Pobezinsky , et al., “Basis of CTLA‐4 Function in Regulatory and Conventional CD4(+) T Cells,” Blood 119, no. 22 (2012): 5155–5163. 22403258 10.1182/blood-2011-11-388918 PMC3369608

[95] B. Rowshanravan , N. Halliday , and D. Sansom , “CTLA‐4: A Moving Target in Immunotherapy,” Blood 131, no. 1 (2018): 58–67. 29118008 10.1182/blood-2017-06-741033 PMC6317697

[96] K. O. Dixon , M. Tabaka , M. A. Schramm , et al., “TIM‐3 Restrains Anti‐Tumour Immunity by Regulating Inflammasome Activation,” Nature 595, no. 7865 (2021): 101–106. 34108686 10.1038/s41586-021-03626-9 PMC8627694

[97] L. Monney , C. A. Sabatos , J. L. Gaglia , et al., “Th1‐Specific Cell Surface Protein Tim‐3 Regulates Macrophage Activation and Severity of an Autoimmune Disease,” Nature 415, no. 6871 (2002): 536–541. 11823861 10.1038/415536a

[98] C. Santiago , A. Ballesteros , L. Martínez‐Muñoz , et al., “Structures of T Cell Immunoglobulin Mucin Protein 4 Show a Metal‐Ion‐Dependent Ligand Binding Site Where Phosphatidylserine Binds,” Immunity 27, no. 6 (2007): 941–951. 18083575 10.1016/j.immuni.2007.11.008 PMC2330274

[99] K. Dixon , G. Lahore , and V. Kuchroo , “Beyond T Cell Exhaustion: TIM‐3 Regulation of Myeloid Cells,” Science Immunology 9, no. 93 (2024): eadf2223. 38457514 10.1126/sciimmunol.adf2223

[100] L. Bod , Y. Kye , J. Shi , et al., “B‐Cell‐Specific Checkpoint Molecules That Regulate Anti‐Tumour Immunity,” Nature 619, no. 7969 (2023): 348–356. 37344597 10.1038/s41586-023-06231-0 PMC10795478

[101] W. Zhong , X. Liu , Z. Zhu , Q. Li , and K. Li , “High Levels of Tim‐3(+)Foxp3(+)Treg Cells in the Tumor Microenvironment Is a Prognostic Indicator of Poor Survival of Diffuse Large B Cell Lymphoma Patients,” International Immunopharmacology 96 (2021): 107662. 33864956 10.1016/j.intimp.2021.107662

[102] Y. Asahina , S. Kamitori , T. Takao , N. Nishi , and H. Hojo , “Chemoenzymatic Synthesis of the Immunoglobulin Domain of Tim‐3 Carrying a Complex‐Type N‐Glycan by Using a One‐Pot Ligation,” Angewandte Chemie 52, no. 37 (2013): 9733–9737. 23868473 10.1002/anie.201303073

[103] Y. Kikushige , “TIM‐3 in Normal and Malignant Hematopoiesis: Structure, Function, and Signaling Pathways,” Cancer Science 112, no. 9 (2021): 3419–3426. 34159709 10.1111/cas.15042 PMC8409405

[104] R. Ferris , B. Lu , and L. Kane , “Too Much of a Good Thing? Tim‐3 and TCR Signaling in T Cell Exhaustion,” Journal of Immunology 193, no. 4 (2014): 1525–1530. 10.4049/jimmunol.1400557 PMC4120324 25086175

[105] M. Rangachari , C. Zhu , K. Sakuishi , et al., “Bat3 Promotes T Cell Responses and Autoimmunity by Repressing Tim‐3–mediated Cell Death and Exhaustion,” Nature Medicine 18, no. 9 (2012): 1394–1400. 10.1038/nm.2871 PMC3491118 22863785

[106] S. Chiba , M. Baghdadi , H. Akiba , et al., “Tumor‐Infiltrating DCs Suppress Nucleic Acid‐Mediated Innate Immune Responses Through Interactions Between the Receptor TIM‐3 and the Alarmin HMGB1,” Nature Immunology 13, no. 9 (2012): 832–842. 22842346 10.1038/ni.2376 PMC3622453

[107] Y. Huang , C. Zhu , Y. Kondo , et al., “CEACAM1 Regulates TIM‐3‐Mediated Tolerance and Exhaustion,” Nature 517, no. 7534 (2015): 386–390. 25363763 10.1038/nature13848 PMC4297519

[108] C. M. Smith , A. Li , N. Krishnamurthy , and M. A. Lemmon , “Phosphatidylserine Binding Directly Regulates TIM‐3 Function,” Biochemical Journal 478, no. 17 (2021): 3331–3349. 34435619 10.1042/BCJ20210425 PMC8454703

[109] Y. Che , Z. Luo , Y. Cao , et al., “Integrated Pathological Analysis to Develop a Gal‐9 Based Immune Survival Stratification to Predict the Outcome of Lung Large Cell Neuroendocrine Carcinoma and Its Usefulness in Immunotherapy,” International Journal of Biological Sciences 18, no. 15 (2022): 5913–5927. 36263183 10.7150/ijbs.76936 PMC9576518

[110] J. Jiao , D. Jiao , F. Yang , et al., “Galectin‐9 Expression Predicts Poor Prognosis in Hepatitis B Virus‐Associated Hepatocellular Carcinoma,” Aging (Albany NY) 14, no. 4 (2022): 1879–1890. 35202002 10.18632/aging.203909 PMC8908941

[111] P. Chen , L. Zhang , W. Zhang , et al., “Galectin‐9‐Based Immune Risk Score Model Helps to Predict Relapse in Stage I‐III Small Cell Lung Cancer,” Journal for ImmunoTherapy of Cancer 8, no. 2 (2020): e001391. 33082168 10.1136/jitc-2020-001391 PMC7577067

[112] L. Liang , Y. Zhang , Y. Shen , et al., “Aberrantly Expressed Galectin‐9 Is Involved in the Immunopathogenesis of Anti‐MDA5‐Positive Dermatomyositis‐Associated Interstitial Lung Disease,” Frontiers in Cell and Developmental Biology 9 (2021): 628128. 33842457 10.3389/fcell.2021.628128 PMC8027128

[113] R. Yang , L. Sun , C. Li , et al., “Galectin‐9 Interacts With PD‐1 and TIM‐3 to Regulate T Cell Death and Is a Target for Cancer Immunotherapy,” Nature Communications 12, no. 1 (2021): 832. 10.1038/s41467-021-21099-2 PMC7864927 33547304

[114] S. Huang , D. Liu , J. Sun , et al., “Tim‐3 Regulates Sepsis‐Induced Immunosuppression by Inhibiting the NF‐κB Signaling Pathway in CD4 T Cells,” Molecular Therapy 30, no. 3 (2022): 1227–1238. 34933101 10.1016/j.ymthe.2021.12.013 PMC8899604

[115] L. Tang , G. Li , Y. Zheng , et al., “Tim‐3 Relieves Experimental Autoimmune Encephalomyelitis by Suppressing MHC‐II,” Frontiers in Immunology 12 (2021): 770402. 35095844 10.3389/fimmu.2021.770402 PMC8793033

[116] X. Jiang , T. Zhou , Y. Xiao , et al., “Tim‐3 Promotes Tumor‐Promoting M2 Macrophage Polarization by Binding to STAT1 and Suppressing the STAT1‐miR‐155 Signaling Axis,” Oncoimmunology 5, no. 9 (2016): e1211219. 27757304 10.1080/2162402X.2016.1211219 PMC5048770

[117] X. Jiang , J. Yu , Q. Shi , et al., “Tim‐3 Promotes Intestinal Homeostasis in DSS Colitis by Inhibiting M1 Polarization of Macrophages,” Clinical Immunology 160, no. 2 (2015): 328–335. 26208474 10.1016/j.clim.2015.07.008

[118] Á. de Mingo Pulido , K. Hänggi , D. P. Celias , et al., “The Inhibitory Receptor TIM‐3 Limits Activation of the cGAS‐STING Pathway in Intra‐Tumoral Dendritic Cells by Suppressing Extracellular DNA Uptake,” Immunity 54, no. 6 (2021): 1154–1167.e7. 33979578 10.1016/j.immuni.2021.04.019 PMC8192496

[119] X. Li , H. Lu , Y. Gu , et al., “Tim‐3 Suppresses the Killing Effect of Vγ9Vδ2 T Cells on Colon Cancer Cells by Reducing Perforin and Granzyme B Expression,” Experimental Cell Research 386, no. 1 (2020): 111719. 31726050 10.1016/j.yexcr.2019.111719

[120] F. Liu , Y. Liu , and Z. Chen , “Tim‐3 Expression and Its Role in Hepatocellular Carcinoma,” Journal of Hematology & Oncology 11, no. 1 (2018): 126. 30309387 10.1186/s13045-018-0667-4 PMC6182863

[121] C. Zhang , H. Shen , T. Yang , et al., “A Single‐Cell Analysis Reveals Tumor Heterogeneity and Immune Environment of Acral Melanoma,” Nature Communications 13, no. 1 (2022): 7250. 10.1038/s41467-022-34877-3 PMC9700682 36433984

[122] L. C. Ndhlovu , S. Lopez‐Vergès , J. D. Barbour , et al., “Tim‐3 Marks Human Natural Killer Cell Maturation and Suppresses Cell‐Mediated Cytotoxicity,” Blood 119, no. 16 (2012): 3734–3743. 22383801 10.1182/blood-2011-11-392951 PMC3335380

[123] Á. de Mingo Pulido , A. Gardner , S. Hiebler , et al., “TIM‐3 Regulates CD103(+) Dendritic Cell Function and Response to Chemotherapy in Breast Cancer,” Cancer Cell 33, no. 1 (2018): 60–74.e6. 29316433 10.1016/j.ccell.2017.11.019 PMC5764109

[124] J. Fucikova , J. Rakova , M. Hensler , et al., “TIM‐3 Dictates Functional Orientation of the Immune Infiltrate in Ovarian Cancer,” Clinical Cancer Research 25, no. 15 (2019): 4820–4831. 31076549 10.1158/1078-0432.CCR-18-4175

[125] I. Datar , M. F. Sanmamed , J. Wang , et al., “Expression Analysis and Significance of PD‐1, LAG‐3, and TIM‐3 in Human Non‐Small Cell Lung Cancer Using Spatially Resolved and Multiparametric Single‐Cell Analysis,” Clinical Cancer Research 25, no. 15 (2019): 4663–4673. 31053602 10.1158/1078-0432.CCR-18-4142 PMC7444693

[126] Y. Kikushige , T. Miyamoto , J. Yuda , et al., “A TIM‐3/Gal‐9 Autocrine Stimulatory Loop Drives Self‐Renewal of Human Myeloid Leukemia Stem Cells and Leukemic Progression,” Cell Stem Cell 17, no. 3 (2015): 341–352. 26279267 10.1016/j.stem.2015.07.011

[127] R. Bouguerra , S. El Hajji , C. Wassmer , et al., “Hepatectomy Alters Adjuvant Anti‐PD‐1 Action in a Mouse Model of HCC but Does Not Compromise Neoadjuvant Efficacy,” Hepatology (2025). 10.1097/HEP.0000000000001575 41128453

[128] L. M. McLane , S. F. Ngiow , Z. Chen , et al., “Role of Nuclear Localization in the Regulation and Function of T‐Bet and Eomes in Exhausted CD8 T Cells,” Cell Reports 35, no. 6 (2021): 109120. 33979613 10.1016/j.celrep.2021.109120 PMC8195461

[129] Y. Lai , S. Wang , T. Ren , et al., “TIGIT Deficiency Promotes Autoreactive CD4(+) T‐Cell Responses Through a Metabolic‒Epigenetic Mechanism in Autoimmune Myositis,” Nature Communications 16, no. 1 (2025): 4502. 10.1038/s41467-025-59786-z PMC12081758 40374622

[130] N. Joller , A. Anderson , and V. Kuchroo , “LAG‐3, TIM‐3, and TIGIT: Distinct Functions in Immune Regulation,” Immunity 57, no. 2 (2024): 206–222. 38354701 10.1016/j.immuni.2024.01.010 PMC10919259

[131] S. Liu , H. Zhang , M. Li , et al., “Recruitment of Grb2 and SHIP1 by the ITT‐Like Motif of TIGIT Suppresses Granule Polarization and Cytotoxicity of NK Cells,” Cell Death and Differentiation 20, no. 3 (2013): 456–464. 23154388 10.1038/cdd.2012.141 PMC3569986

[132] X. Huang , X. Zhang , E. Li , et al., “VISTA: An Immune Regulatory Protein Checking Tumor and Immune Cells in Cancer Immunotherapy,” Journal of Hematology & Oncology 13, no. 1 (2020): 83. 32600443 10.1186/s13045-020-00917-y PMC7325042

[133] C. Guy , D. M. Mitrea , P. Chou , et al., “LAG3 Associates With TCR‐CD3 Complexes and Suppresses Signaling by Driving Co‐Receptor‐Lck Dissociation,” Nature Immunology 23, no. 5 (2022): 757–767. 35437325 10.1038/s41590-022-01176-4 PMC9106921

[134] C. Solinas , C. Gu‐Trantien , and K. Willard‐Gallo , “The Rationale Behind Targeting the ICOS‐ICOS Ligand Costimulatory Pathway in Cancer Immunotherapy,” ESMO Open 5, no. 1 (2020): e000544. 32516116 10.1136/esmoopen-2019-000544 PMC7003380

[135] R. Singh , Y. Kim , S. Lee , H. Eom , and B. K. Choi , “4‐1BB Immunotherapy: Advances and Hurdles,” Experimental & Molecular Medicine 56, no. 1 (2024): 32–39. 38172595 10.1038/s12276-023-01136-4 PMC10834507

[136] G. Liu and P. Luo , “Targeting CD137 (4‐1BB) Towards Improved Safety and Efficacy for Cancer Immunotherapy,” Frontiers in Immunology 14 (2023): 1208788. 37334375 10.3389/fimmu.2023.1208788 PMC10272836

[137] E. Guttman‐Yassky , M. Croft , B. Geng , et al., “The Role of OX40 Ligand/OX40 Axis Signalling in Atopic Dermatitis,” British Journal of Dermatology 191, no. 4 (2024): 488–496. 38836560 10.1093/bjd/ljae230

[138] B. Thapa , S. Kato , D. Nishizaki , et al., “OX40/OX40 Ligand and Its Role in Precision Immune Oncology,” Cancer and Metastasis Reviews 43, no. 3 (2024): 1001–1013. 38526805 10.1007/s10555-024-10184-9 PMC11300540

[139] Y. Zhou , A. Richmond , and C. Yan , “Harnessing the Potential of CD40 Agonism in Cancer Therapy,” Cytokine & Growth Factor Reviews 75 (2024): 40–56. 38102001 10.1016/j.cytogfr.2023.11.002 PMC10922420

[140] H. Ledford , “Melanoma Drug Wins US Approval,” Nature 471, no. 7340 (2011): 561. 21455150 10.1038/471561a

[141] C. Robert , G. V. Long , B. Brady , et al., “Five‐Year Outcomes With Nivolumab in Patients With Wild‐Type BRAF Advanced Melanoma,” Journal of Clinical Oncology 38, no. 33 (2020): 3937–3946. 32997575 10.1200/JCO.20.00995 PMC7676881

[142] H. Borghaei , L. Paz‐Ares , L. Horn , et al., “Nivolumab Versus Docetaxel in Advanced Nonsquamous Non‐Small‐Cell Lung Cancer,” New England Journal of Medicine 373, no. 17 (2015): 1627–1639. 26412456 10.1056/NEJMoa1507643 PMC5705936

[143] C. Robert , J. Schachter , G. V. Long , et al., “Pembrolizumab Versus Ipilimumab in Advanced Melanoma,” New England Journal of Medicine 372, no. 26 (2015): 2521–2532. 25891173 10.1056/NEJMoa1503093

[144] M. Reck , D. Rodríguez‐Abreu , A. G. Robinson , et al., “Pembrolizumab Versus Chemotherapy for PD‐L1‐Positive Non‐Small‐Cell Lung Cancer,” New England Journal of Medicine 375, no. 19 (2016): 1823–1833. 27718847 10.1056/NEJMoa1606774

[145] A. Rittmeyer , F. Barlesi , D. Waterkamp , et al., “Atezolizumab Versus Docetaxel in Patients With Previously Treated Non‐Small‐Cell Lung Cancer (OAK): A Phase 3, Open‐Label, Multicentre Randomised Controlled Trial,” Lancet 389, no. 10066 (2017): 255–265. 27979383 10.1016/S0140-6736(16)32517-X PMC6886121

[146] R. L. Ferris , G. Blumenschein , J. Fayette , et al., “Nivolumab for Recurrent Squamous‐Cell Carcinoma of the Head and Neck,” New England Journal of Medicine 375, no. 19 (2016): 1856–1867. 27718784 10.1056/NEJMoa1602252 PMC5564292

[147] R. J. Motzer , B. Escudier , D. F. McDermott , et al., “Nivolumab Versus Everolimus in Advanced Renal‐Cell Carcinoma,” New England Journal of Medicine 373, no. 19 (2015): 1803–1813. 26406148 10.1056/NEJMoa1510665 PMC5719487

[148] A. Betof Warner , J. S. Palmer , A. N. Shoushtari , et al., “Long‐Term Outcomes and Responses to Retreatment in Patients With Melanoma Treated With PD‐1 Blockade,” Journal of Clinical Oncology 38, no. 15 (2020): 1655–1663. 32053428 10.1200/JCO.19.01464 PMC7238490

[149] F. Garrido , N. Aptsiauri , E. M. Doorduijn , A. M. Garcia Lora , and T. van Hall , “The Urgent Need to Recover MHC Class I in Cancers for Effective Immunotherapy,” Current Opinion in Immunology 39 (2016): 44–51. 26796069 10.1016/j.coi.2015.12.007 PMC5138279

[150] F. Exposito , M. Redrado , M. Houry , et al., “PTEN Loss Confers Resistance to Anti‐PD‐1 Therapy in Non‐Small Cell Lung Cancer by Increasing Tumor Infiltration of Regulatory T Cells,” Cancer Research 83, no. 15 (2023): 2513–2526. 37311042 10.1158/0008-5472.CAN-22-3023

[151] H. Harb , E. Stephen‐Victor , E. Crestani , et al., “A Regulatory T Cell Notch4‐GDF15 Axis Licenses Tissue Inflammation in Asthma,” Nature Immunology 21, no. 11 (2020): 1359–1370. 32929274 10.1038/s41590-020-0777-3 PMC7578174

[152] Z. Gong , Q. Jia , J. Chen , et al., “Impaired Cytolytic Activity and Loss of Clonal Neoantigens in Elderly Patients With Lung Adenocarcinoma,” Journal of Thoracic Oncology 14, no. 5 (2019): 857–866. 30768970 10.1016/j.jtho.2019.01.024

[153] M. Borgeaud , J. Sandoval , M. Obeid , et al., “Novel Targets for Immune‐Checkpoint Inhibition in Cancer,” Cancer Treatment Reviews 120 (2023): 102614. 37603905 10.1016/j.ctrv.2023.102614

[154] C. Chen , F. Zhao , J. Peng , et al., “Soluble Tim‐3 Serves as a Tumor Prognostic Marker and Therapeutic Target for CD8(+) T Cell Exhaustion and Anti‐PD‐1 Resistance,” Cell Reports Medicine 5, no. 8 (2024): 101686. 39168104 10.1016/j.xcrm.2024.101686 PMC11384939

[155] M. A. Curran , W. Montalvo , H. Yagita , and J. P. Allison , “PD‐1 and CTLA‐4 Combination Blockade Expands Infiltrating T Cells and Reduces Regulatory T and Myeloid Cells Within B16 Melanoma Tumors,” Proceedings of the National Academy of Sciences of the United States of America 107, no. 9 (2010): 4275–4280. 20160101 10.1073/pnas.0915174107 PMC2840093

[156] J. D. Wolchok , H. Kluger , M. K. Callahan , et al., “Nivolumab Plus Ipilimumab in Advanced Melanoma,” New England Journal of Medicine 369, no. 2 (2013): 122–133. 23724867 10.1056/NEJMoa1302369 PMC5698004

[157] J. Larkin , V. Chiarion‐Sileni , R. Gonzalez , et al., “Five‐Year Survival With Combined Nivolumab and Ipilimumab in Advanced Melanoma,” New England Journal of Medicine 381, no. 16 (2019): 1535–1546. 31562797 10.1056/NEJMoa1910836

[158] J. D. Wolchok , V. Chiarion‐Sileni , P. Rutkowski , et al., “Final, 10‐Year Outcomes With Nivolumab Plus Ipilimumab in Advanced Melanoma,” New England Journal of Medicine 392, no. 1 (2025): 11–22. 39282897 10.1056/NEJMoa2407417 PMC12080919

[159] N. Tannir , L. Albigès , D. McDermott , et al., “Nivolumab Plus Ipilimumab Versus Sunitinib for First‐Line Treatment of Advanced Renal Cell Carcinoma: Extended 8‐Year Follow‐Up Results of Efficacy and Safety From the Phase III CheckMate 214 Trial,” Annals of Oncology 35, no. 11 (2024): 1026–1038. 39098455 10.1016/j.annonc.2024.07.727 PMC11907766

[160] I. Melero , T. Yau , Y. Kang , et al., “Nivolumab Plus Ipilimumab Combination Therapy in Patients With Advanced Hepatocellular Carcinoma Previously Treated With Sorafenib: 5‐Year Results From CheckMate 040,” Annals of Oncology 35, no. 6 (2024): 537–548. 38844309 10.1016/j.annonc.2024.03.005

[161] K. Kato , Y. Doki , I. Chau , et al., “Nivolumab Plus Chemotherapy or Ipilimumab Versus Chemotherapy in Patients With Advanced Esophageal Squamous Cell Carcinoma (CheckMate 648): 29‐Month Follow‐Up From a Randomized, Open‐Label, Phase III Trial,” Cancer Medicine 13, no. 9 (2024): e7235. 38716626 10.1002/cam4.7235 PMC11077338

[162] B. Sangro , S. Chan , R. Kelley , et al., “Four‐Year Overall Survival Update From the Phase III HIMALAYA Study of Tremelimumab Plus Durvalumab in Unresectable Hepatocellular Carcinoma,” Annals of Oncology 35, no. 5 (2024): 448–457. 38382875 10.1016/j.annonc.2024.02.005

[163] M. D. Hellmann , N. A. Rizvi , J. W. Goldman , et al., “Nivolumab Plus Ipilimumab as First‐Line Treatment for Advanced Non‐Small‐Cell Lung Cancer (CheckMate 012): Results of an Open‐Label, Phase 1, Multicohort Study,” The Lancet Oncology 18, no. 1 (2017): 31–41. 27932067 10.1016/S1470-2045(16)30624-6 PMC5476941

[164] N. Ready , M. D. Hellmann , M. M. Awad , et al., “First‐Line Nivolumab Plus Ipilimumab in Advanced Non‐Small‐Cell Lung Cancer (CheckMate 568): Outcomes by Programmed Death Ligand 1 and Tumor Mutational Burden as Biomarkers,” Journal of Clinical Oncology 37, no. 12 (2019): 992–1000. 30785829 10.1200/JCO.18.01042 PMC6494267

[165] J. R. Brahmer , J. Lee , T. Ciuleanu , et al., “Five‐Year Survival Outcomes With Nivolumab Plus Ipilimumab Versus Chemotherapy as First‐Line Treatment for Metastatic Non‐Small‐Cell Lung Cancer in CheckMate 227,” Journal of Clinical Oncology 41, no. 6 (2023): 1200–1212. 36223558 10.1200/JCO.22.01503 PMC9937094

[166] K. Nutsch , K. L. Banta , T. D. Wu , et al., “TIGIT and PD‐L1 Co‐Blockade Promotes Clonal Expansion of Multipotent, Non‐Exhausted Antitumor T Cells by Facilitating Co‐Stimulation,” Nature Cancer 5, no. 12 (2024): 1834–1851. 39681653 10.1038/s43018-024-00870-6 PMC11663793

[167] H. A. Tawbi , D. Schadendorf , E. J. Lipson , et al., “Relatlimab and Nivolumab Versus Nivolumab in Untreated Advanced Melanoma,” New England Journal of Medicine 386, no. 1 (2022): 24–34. 34986285 10.1056/NEJMoa2109970 PMC9844513

[168] G. V. Long , E. J. Lipson , F. S. Hodi , et al., “First‐Line Nivolumab Plus Relatlimab Versus Nivolumab Plus Ipilimumab in Advanced Melanoma: An Indirect Treatment Comparison Using RELATIVITY‐047 and CheckMate 067 Trial Data,” Journal of Clinical Oncology 42, no. 33 (2024): 3926–3934. 39137386 10.1200/JCO.24.01125 PMC11575907

[169] J. Niu , C. Maurice‐Dror , D. Lee , et al., “First‐in‐Human Phase 1 Study of the Anti‐TIGIT Antibody Vibostolimab as Monotherapy or With Pembrolizumab for Advanced Solid Tumors, Including Non‐Small‐Cell Lung Cancer(☆),” Annals of Oncology 33, no. 2 (2022): 169–180. 34800678 10.1016/j.annonc.2021.11.002

[170] R. Dummer , C. Robert , R. A. Scolyer , et al., “Neoadjuvant Anti‐PD‐1 Alone or in Combination With Anti‐TIGIT or an Oncolytic Virus in Resectable Stage IIIB‐D Melanoma: A Phase 1/2 Trial,” Nature Medicine 31, no. 1 (2025): 144–151. 10.1038/s41591-024-03411-x PMC11750705 39775043

[171] D. Rodriguez‐Abreu , J. Bosch‐Barrera , J. E. Gray , et al., “STAR‐121: A Phase III Randomized Study of Domvanalimab and Zimberelimab in Combination With Chemotherapy Versus Pembrolizumab With Chemotherapy in Untreated Metastatic Non‐Small Cell Lung Cancer With no Actionable Gene Alterations,” Clinical Lung Cancer 25, no. 3 (2024): 274–279. 38310035 10.1016/j.cllc.2023.12.010

[172] J. Sun , X. Zhang , L. Xue , et al., “Structural Insights Into the Unique pH‐Responsive Characteristics of the Anti‐TIGIT Therapeutic Antibody Ociperlimab,” Structure 32, no. 5 (2024): 550–561.e5. 38460520 10.1016/j.str.2024.02.009

[173] S. Frentzas , S. Kao , R. Gao , et al., “AdvanTIG‐105: A Phase I Dose Escalation Study of the Anti‐TIGIT Monoclonal Antibody Ociperlimab in Combination With Tislelizumab in Patients With Advanced Solid Tumors,” Journal for ImmunoTherapy of Cancer 11, no. 10 (2023): e005829. 37857528 10.1136/jitc-2022-005829 PMC10603446

[174] O. Hamid , M. Gutierrez , I. Mehmi , et al., “A Phase 1/2 Study of Retifanlimab (INCMGA00012, Anti–PD‐1), INCAGN02385 (Anti–LAG‐3), and INCAGN02390 (Anti–TIM‐3) Combination Therapy in Patients (Pts) With Advanced Solid Tumors,” Journal of Clinical Oncology 41, no. 16_suppl (2023): 2599–2599.

[175] J. Chauvin , O. Pagliano , J. Fourcade , et al., “TIGIT and PD‐1 Impair Tumor Antigen‐Specific CD8^+^ T Cells in Melanoma Patients,” Journal of Clinical Investigation 125, no. 5 (2015): 2046–2058. 25866972 10.1172/JCI80445 PMC4463210

[176] E. Chiang and I. Mellman , “TIGIT‐CD226‐PVR Axis: Advancing Immune Checkpoint Blockade for Cancer Immunotherapy,” Journal for ImmunoTherapy of Cancer 10, no. 4 (2022): e004711. 35379739 10.1136/jitc-2022-004711 PMC8981293

[177] D. Piovesan , A. E. de Groot , S. Cho , et al., “Fc‐Silent Anti‐TIGIT Antibodies Potentiate Antitumor Immunity Without Depleting Regulatory T Cells,” Cancer Research 84, no. 12 (2024): 1978–1995. 38635895 10.1158/0008-5472.CAN-23-2455

[178] L. Galluzzi , E. Guilbaud , D. Schmidt , G. Kroemer , and F. M. Marincola , “Targeting Immunogenic Cell Stress and Death for Cancer Therapy,” Nature Reviews Drug Discovery 23, no. 6 (2024): 445–460. 38622310 10.1038/s41573-024-00920-9 PMC11153000

[179] Z. Li , X. Lai , S. Fu , et al., “Immunogenic Cell Death Activates the Tumor Immune Microenvironment to Boost the Immunotherapy Efficiency,” Advanced Science (Weinheim, Baden‐Wurttemberg, Germany) 9, no. 22 (2022): e2201734. 35652198 10.1002/advs.202201734 PMC9353475

[180] N. Li , Y. Li , J. Li , S. Tang , H. Gao , and Y. Li , “Correlation of the Abundance of MDSCs, Tregs, PD‐1, and PD‐L1 With the Efficacy of Chemotherapy and Prognosis in Gastric Cancer,” Laboratory Medicine 56, no. 3 (2024): 259–270. 10.1093/labmed/lmae090 39566022

[181] A. Goyal , J. Bauer , J. Hey , et al., “DNMT and HDAC Inhibition Induces Immunogenic Neoantigens From Human Endogenous Retroviral Element‐Derived Transcripts,” Nature Communications 14, no. 1 (2023): 6731. 10.1038/s41467-023-42417-w PMC10593957 37872136

[182] L. Zhang , C. Zhou , S. Zhang , et al., “Chemotherapy Reinforces Anti‐Tumor Immune Response and Enhances Clinical Efficacy of Immune Checkpoint Inhibitors,” Frontiers in Oncology 12 (2022): 939249. 36003765 10.3389/fonc.2022.939249 PMC9393416

[183] Y. Zhang , H. Chen , H. Mo , et al., “Distinct Cellular Mechanisms Underlie Chemotherapies and PD‐L1 Blockade Combinations in Triple‐Negative Breast Cancer,” Cancer Cell 43, no. 3 (2025): 446–463.e7. 39919737 10.1016/j.ccell.2025.01.007

[184] C. J. Langer , S. M. Gadgeel , H. Borghaei , et al., “Carboplatin and Pemetrexed With or Without Pembrolizumab for Advanced, Non‐Squamous Non‐Small‐Cell Lung Cancer: A Randomised, Phase 2 Cohort of the Open‐Label KEYNOTE‐021 Study,” The Lancet Oncology 17, no. 11 (2016): 1497–1508. 27745820 10.1016/S1470-2045(16)30498-3 PMC6886237

[185] M. C. Garassino , S. Gadgeel , E. Esteban , et al., “Patient‐Reported Outcomes Following Pembrolizumab or Placebo Plus Pemetrexed and Platinum in Patients With Previously Untreated, Metastatic, Non‐Squamous Non‐Small‐Cell Lung Cancer (KEYNOTE‐189): A Multicentre, Double‐Blind, Randomised, Placebo‐Controlled, Phase 3 Trial,” The Lancet Oncology 21, no. 3 (2020): 387–397. 32035514 10.1016/S1470-2045(19)30801-0

[186] J. Chao , C. S. Fuchs , K. Shitara , et al., “Assessment of Pembrolizumab Therapy for the Treatment of Microsatellite Instability‐High Gastric or Gastroesophageal Junction Cancer Among Patients in the KEYNOTE‐059, KEYNOTE‐061, and KEYNOTE‐062 Clinical Trials,” JAMA Oncology 7, no. 6 (2021): 895–902. 33792646 10.1001/jamaoncol.2021.0275 PMC8017478

[187] S. Y. Rha , D. Oh , P. Yañez , et al., “Pembrolizumab Plus Chemotherapy Versus Placebo Plus Chemotherapy for HER2‐Negative Advanced Gastric Cancer (KEYNOTE‐859): A Multicentre, Randomised, Double‐Blind, Phase 3 Trial,” The Lancet Oncology 24, no. 11 (2023): 1181–1195. 37875143 10.1016/S1470-2045(23)00515-6

[188] J. Xu , H. Jiang , Y. Pan , et al., “Sintilimab Plus Chemotherapy for Unresectable Gastric or Gastroesophageal Junction Cancer: The ORIENT‐16 Randomized Clinical Trial,” Jama 330, no. 21 (2023): 2064–2074. 38051328 10.1001/jama.2023.19918 PMC10698618

[189] Y. K. Kang , M. Terashima , Y. W. Kim , et al., “Adjuvant Nivolumab Plus Chemotherapy Versus Placebo Plus Chemotherapy for Stage III Gastric or Gastro‐Oesophageal Junction Cancer After Gastrectomy With D2 or More Extensive Lymph‐Node Dissection (ATTRACTION‐5): A Randomised, Multicentre, Double‐Blind, Placebo‐Controlled, Phase 3 Trial,” Lancet Gastroenterology and Hepatology 9, no. 8 (2024): 705–717. 38906161 10.1016/S2468-1253(24)00156-0

[190] K. Shitara , S. Y. Rha , L. S. Wyrwicz , et al., “Neoadjuvant and Adjuvant Pembrolizumab Plus Chemotherapy in Locally Advanced Gastric or Gastro‐Oesophageal Cancer (KEYNOTE‐585): An Interim Analysis of the Multicentre, Double‐Blind, Randomised Phase 3 Study,” The Lancet Oncology 25, no. 2 (2024): 212–224. 38134948 10.1016/S1470-2045(23)00541-7

[191] C. J. Hoimes , T. W. Flaig , M. I. Milowsky , et al., “Enfortumab Vedotin Plus Pembrolizumab in Previously Untreated Advanced Urothelial Cancer,” Journal of Clinical Oncology 41, no. 1 (2023): 22–31. 36041086 10.1200/JCO.22.01643 PMC10476837

[192] M. S. van der Heijden , G. Sonpavde , T. Powles , et al., “Nivolumab Plus Gemcitabine‐Cisplatin in Advanced Urothelial Carcinoma,” New England Journal of Medicine 389, no. 19 (2023): 1778–1789. 37870949 10.1056/NEJMoa2309863 PMC12314471

[193] J. Zhai , X. Gu , Y. Liu , Y. Hu , Y. Jiang , and Z. Zhang , “Chemotherapeutic and Targeted Drugs‐Induced Immunogenic Cell Death in Cancer Models and Antitumor Therapy: An Update Review,” Frontiers in Pharmacology 14 (2023): 1152934. 37153795 10.3389/fphar.2023.1152934 PMC10160433

[194] A. Basu , A. Hoerning , D. Datta , et al., “Cutting Edge: Vascular Endothelial Growth Factor‐Mediated Signaling in Human CD45RO+ CD4+ T Cells Promotes Akt and ERK Activation and Costimulates IFN‐Gamma Production,” Journal of Immunology 184, no. 2 (2010): 545–549. 10.4049/jimmunol.0900397 PMC3495980 20008289

[195] S. Karakhanova , J. Link , M. Heinrich , et al., “Characterization of Myeloid Leukocytes and Soluble Mediators in Pancreatic Cancer: Importance of Myeloid‐Derived Suppressor Cells,” Oncoimmunology 4, no. 4 (2015): e998519. 26137414 10.1080/2162402X.2014.998519 PMC4485765

[196] S. Peng , R. Wang , X. Zhang , et al., “EGFR‐TKI Resistance Promotes Immune Escape in Lung Cancer via Increased PD‐L1 Expression,” Molecular Cancer 18, no. 1 (2019): 165. 31747941 10.1186/s12943-019-1073-4 PMC6864970

[197] D. Munn and A. Mellor , “IDO in the Tumor Microenvironment: Inflammation, Counter‐Regulation, and Tolerance,” Trends in Immunology 37, no. 3 (2016): 193–207. 26839260 10.1016/j.it.2016.01.002 PMC4916957

[198] R. S. Finn , S. Qin , M. Ikeda , et al., “Atezolizumab Plus Bevacizumab in Unresectable Hepatocellular Carcinoma,” New England Journal of Medicine 382, no. 20 (2020): 1894–1905. 32402160 10.1056/NEJMoa1915745

[199] P. A. Ascierto , D. Stroyakovskiy , H. Gogas , et al., “Overall Survival With First‐Line Atezolizumab in Combination With Vemurafenib and Cobimetinib in BRAF(V600) Mutation‐Positive Advanced Melanoma (IMspire150): Second Interim Analysis of a Multicentre, Randomised, Phase 3 Study,” The Lancet Oncology 24, no. 1 (2023): 33–44. 36460017 10.1016/S1470-2045(22)00687-8

[200] C. Cai , I. Yunusa , and A. Tarhini , “Estimated Cost‐Effectiveness of Atezolizumab Plus Cobimetinib and Vemurafenib for Treatment of BRAF V600 Variation Metastatic Melanoma,” JAMA Network Open 4, no. 11 (2021): e2132262. 34762112 10.1001/jamanetworkopen.2021.32262 PMC8586909

[201] R. Gutzmer , D. Stroyakovskiy , H. Gogas , et al., “Atezolizumab, Vemurafenib, and Cobimetinib as First‐Line Treatment for Unresectable Advanced BRAF(V600) Mutation‐Positive Melanoma (IMspire150): Primary Analysis of the Randomised, Double‐Blind, Placebo‐Controlled, Phase 3 Trial,” Lancet 395, no. 10240 (2020): 1835–1844. 32534646 10.1016/S0140-6736(20)30934-X

[202] C. Robert , K. Lewis , R. Gutzmer , et al., “Biomarkers of Treatment Benefit With Atezolizumab Plus Vemurafenib Plus Cobimetinib in BRAF(V600) Mutation‐Positive Melanoma,” Annals of Oncology 33, no. 5 (2022): 544–555. 35131452 10.1016/j.annonc.2022.01.076

[203] J. Saal , M. Eckstein , M. Ritter , et al., “Dissection of Progressive Disease Patterns for a Modified Classification for Immunotherapy,” JAMA Oncology 11, no. 2 (2025): 154–161. 39724246 10.1001/jamaoncol.2024.5672 PMC11843377

[204] X. Chen , A. Gao , F. Zhang , et al., “ILT4 Inhibition Prevents TAM‐ and Dysfunctional T Cell‐Mediated Immunosuppression and Enhances the Efficacy of Anti‐PD‐L1 Therapy in NSCLC With EGFR Activation,” Theranostics 11, no. 7 (2021): 3392–3416. 33537094 10.7150/thno.52435 PMC7847666

[205] E. Sugiyama , Y. Togashi , Y. Takeuchi , et al., “Blockade of EGFR Improves Responsiveness to PD‐1 Blockade in EGFR‐Mutated Non‐Small Cell Lung Cancer,” Science Immunology 5, no. 43 (2020): eaav3937. 32005679 10.1126/sciimmunol.aav3937

[206] Y. Zhao , G. Chen , J. Chen , et al., “AK112, a Novel PD‐1/VEGF Bispecific Antibody, in Combination With Chemotherapy in Patients With Advanced Non‐Small Cell Lung Cancer (NSCLC): An Open‐Label, Multicenter, Phase II Trial,” EClinicalMedicine 62 (2023): 102106. 37593227 10.1016/j.eclinm.2023.102106 PMC10430160

[207] J. C. Yang , S. M. Gadgeel , L. V. Sequist , et al., “Pembrolizumab in Combination With Erlotinib or Gefitinib as First‐Line Therapy for Advanced NSCLC With Sensitizing EGFR Mutation,” Journal of Thoracic Oncology 14, no. 3 (2019): 553–559. 30529597 10.1016/j.jtho.2018.11.028

[208] N. Nogami , F. Barlesi , M. A. Socinski , et al., “IMpower150 Final Exploratory Analyses for Atezolizumab Plus Bevacizumab and Chemotherapy in Key NSCLC Patient Subgroups With EGFR Mutations or Metastases in the Liver or Brain,” Journal of Thoracic Oncology 17, no. 2 (2022): 309–323. 34626838 10.1016/j.jtho.2021.09.014

[209] M. A. Socinski , M. Nishio , R. M. Jotte , et al., “IMpower150 Final Overall Survival Analyses for Atezolizumab Plus Bevacizumab and Chemotherapy in First‐Line Metastatic Nonsquamous NSCLC,” Journal of Thoracic Oncology 16, no. 11 (2021): 1909–1924. 34311108 10.1016/j.jtho.2021.07.009

[210] L. Rimassa , R. Finn , and B. Sangro , “Combination Immunotherapy for Hepatocellular Carcinoma,” Journal of Hepatology 79, no. 2 (2023): 506–515. 36933770 10.1016/j.jhep.2023.03.003

[211] A. Venniyoor , “Synergism Between Anti‐Angiogenic and Immune Checkpoint Inhibitor Drugs: A Hypothesis,” Medical Hypotheses 146 (2021): 110399. 33239232 10.1016/j.mehy.2020.110399

[212] F. A. Büttner , S. Winter , V. Stühler , et al., “A Novel Molecular Signature Identifies Mixed Subtypes in Renal Cell Carcinoma With Poor Prognosis and Independent Response to Immunotherapy,” Genome Medicine 14, no. 1 (2022): 105. 36109798 10.1186/s13073-022-01105-y PMC9476269

[213] R. H. I. Andtbacka , B. Curti , G. A. Daniels , et al., “Clinical Responses of Oncolytic Coxsackievirus A21 (V937) in Patients With Unresectable Melanoma,” Journal of Clinical Oncology 39, no. 34 (2021): 3829–3838. 34464163 10.1200/JCO.20.03246

[214] Coxsackievirus A21 Synergizes With Checkpoint Inhibitors. Cancer Discovery 2017. 7(6): p. Of9. 10.1158/2159-8290.CD-NB2017-051 28381405

[215] A. W. Silk , S. J. O'Day , H. L. Kaufman , et al., “A Phase 1b Single‐Arm Trial of Intratumoral Oncolytic Virus V937 in Combination With Pembrolizumab in Patients With Advanced Melanoma: Results From the CAPRA Study,” Cancer Immunology, Immunotherapy 72, no. 6 (2023): 1405–1415. 36445410 10.1007/s00262-022-03314-1 PMC10198910

[216] C. M. Rudin , H. S. Pandha , M. Zibelman , et al., “Phase 1, Open‐Label, Dose‐Escalation Study on the Safety, Pharmacokinetics, and Preliminary Efficacy of Intravenous Coxsackievirus A21 (V937), With or Without Pembrolizumab, in Patients With Advanced Solid Tumors,” Journal for ImmunoTherapy of Cancer 11, no. 1 (2023): e005007. 36669791 10.1136/jitc-2022-005007 PMC9872507

[217] L. Yi , Z. Ning , L. Xu , et al., “The Combination Treatment of Oncolytic Adenovirus H101 With Nivolumab for Refractory Advanced Hepatocellular Carcinoma: An Open‐Label, Single‐Arm, Pilot Study,” ESMO Open 9, no. 2 (2024): 102239. 38325225 10.1016/j.esmoop.2024.102239 PMC10937204

[218] M. McLaughlin , E. C. Patin , M. Pedersen , et al., “Inflammatory Microenvironment Remodelling by Tumour Cells After Radiotherapy,” Nature Reviews Cancer 20, no. 4 (2020): 203–217. 32161398 10.1038/s41568-020-0246-1

[219] W. Zhen , R. Weichselbaum , and W. Lin , “Nanoparticle‐Mediated Radiotherapy Remodels the Tumor Microenvironment to Enhance Antitumor Efficacy,” Advanced Materials 35, no. 21 (2023): e2206370. 36524978 10.1002/adma.202206370 PMC10213153

[220] O. Ozpiskin , L. Zhang , and J. Li , “Immune Targets in the Tumor Microenvironment Treated by Radiotherapy,” Theranostics 9, no. 5 (2019): 1215–1231. 30867826 10.7150/thno.32648 PMC6401500

[221] M. Jarosz‐Biej , R. Smolarczyk , T. Cichoń , and N. Kułach , “Tumor Microenvironment as a “Game Changer” in Cancer Radiotherapy,” International Journal of Molecular Sciences 20, no. 13 (2019): 3212. 31261963 10.3390/ijms20133212 PMC6650939

[222] M. Yang , J. Li , P. Gu , and X. Fan , “The Application of Nanoparticles in Cancer Immunotherapy: Targeting Tumor Microenvironment,” Bioactive Materials 6, no. 7 (2021): 1973–1987. 33426371 10.1016/j.bioactmat.2020.12.010 PMC7773537

[223] M. Ashrafizadeh , B. Farhood , A. E. Musa , S. Taeb , and M. Najafi , “The Interactions and Communications in Tumor Resistance to Radiotherapy: Therapy Perspectives,” International Immunopharmacology 87 (2020): 106807. 32683299 10.1016/j.intimp.2020.106807

[224] M. Spaas , N. Sundahl , V. Kruse , et al., “Checkpoint Inhibitors in Combination With Stereotactic Body Radiotherapy in Patients With Advanced Solid Tumors: The CHEERS Phase 2 Randomized Clinical Trial,” JAMA Oncology 9, no. 9 (2023): 1205–1213. 37410476 10.1001/jamaoncol.2023.2132 PMC10326732

[225] P. Zhou , D. Chen , B. Zhu , et al., “Stereotactic Body Radiotherapy Is Effective in Modifying the Tumor Genome and Tumor Immune Microenvironment in Non‐Small Cell Lung Cancer or Lung Metastatic Carcinoma,” Frontiers in Immunology 11 (2020): 594212. 33552051 10.3389/fimmu.2020.594212 PMC7862546

[226] F. Tong , Y. Sun , Y. Zhu , et al., “Making “Cold” Tumors “Hot”‐ Radiotherapy Remodels the Tumor Immune Microenvironment of Pancreatic Cancer to Benefit From Immunotherapy: A Case Report,” Frontiers in Immunology 14 (2023): 1277810. 38179049 10.3389/fimmu.2023.1277810 PMC10765511

[227] L. Sevenich , “Turning “Cold” Into “Hot” Tumors‐Opportunities and Challenges for Radio‐Immunotherapy Against Primary and Metastatic Brain Cancers,” Frontiers in Oncology 9 (2019): 163. 30941312 10.3389/fonc.2019.00163 PMC6433980

[228] Y. Ding , Y. Feng , Y. Ye , et al., “High and Low Dose Radiotherapy Combined With ICIs for MSS Colorectal Cancer Patients With Liver Metastases: A Phase I Study (HaRyPOT),” Frontiers in Oncology 15 (2025): 1503517. 39980556 10.3389/fonc.2025.1503517 PMC11839429

[229] W. Wu , T. Chia , J. Lu , et al., “IL‐2Rα‐Biased Agonist Enhances Antitumor Immunity by Invigorating Tumor‐Infiltrating CD25(+)CD8(+) T Cells,” Nature Cancer 4, no. 9 (2023): 1309–1325. 37550516 10.1038/s43018-023-00612-0

[230] L. Codarri Deak , V. Nicolini , M. Hashimoto , et al., “PD‐1‐cis IL‐2R Agonism Yields Better Effectors From Stem‐Like CD8(+) T Cells,” Nature 610, no. 7930 (2022): 161–172. 36171284 10.1038/s41586-022-05192-0 PMC9534752

[231] P. Monk , E. Lam , A. Mortazavi , et al., “A Phase I Study of High‐Dose Interleukin‐2 With Sorafenib in Patients With Metastatic Renal Cell Carcinoma and Melanoma,” Journal of Immunotherapy 37, no. 3 (2014): 180–186. 24598448 10.1097/CJI.0000000000000023 PMC3966917

[232] T. Flaadt , R. L. Ladenstein , M. Ebinger , et al., “Anti‐GD2 Antibody Dinutuximab Beta and Low‐Dose Interleukin 2 After Haploidentical Stem‐Cell Transplantation in Patients With Relapsed Neuroblastoma: A Multicenter, Phase I/II Trial,” Journal of Clinical Oncology 41, no. 17 (2023): 3135–3148. 36854071 10.1200/JCO.22.01630 PMC10256422

[233] J. M. Wrangle , V. Velcheti , M. R. Patel , et al., “ALT‐803, an IL‐15 Superagonist, in Combination With Nivolumab in Patients With Metastatic Non‐Small Cell Lung Cancer: A Non‐Randomised, Open‐Label, Phase 1b Trial,” The Lancet Oncology 19, no. 5 (2018): 694–704. 29628312 10.1016/S1470-2045(18)30148-7 PMC6089612

[234] T. J. Herzog , J. L. Hays , J. N. Barlin , et al., “ARTISTRY‐7: Phase III Trial of Nemvaleukin Alfa Plus Pembrolizumab vs Chemotherapy for Platinum‐Resistant Ovarian Cancer,” Future Oncology 19, no. 23 (2023): 1577–1591. 37334673 10.2217/fon-2023-0246

[235] R. Nguyen , E. Doubrovina , C. M. Mousset , et al., “Cooperative Armoring of CAR and TCR T Cells by T Cell‐Restricted IL15 and IL21 Universally Enhances Solid Tumor Efficacy,” Clinical Cancer Research 30, no. 8 (2024): 1555–1566. 37910044 10.1158/1078-0432.CCR-23-1872 PMC11018485

[236] T. J. Guzik , R. Nosalski , P. Maffia , and G. R. Drummond , “Immune and Inflammatory Mechanisms in Hypertension,” Nature Reviews Cardiology 21, no. 6 (2024): 396–416. 38172242 10.1038/s41569-023-00964-1

[237] R. M. Awad , Y. De Vlaeminck , F. Meeus , et al., “In Vitro Modelling of Local Gene Therapy With IL‐15/IL‐15Rα and a PD‐L1 Antagonist in Melanoma Reveals an Interplay Between NK Cells and CD4(+) T Cells,” Scientific Reports 13, no. 1 (2023): 18995. 37923822 10.1038/s41598-023-45948-w PMC10624833

[238] S. Martomo and J. Patel , “Evaluation of the Clinical Molecule Anti‐Human‐PD‐L1/IL‐15 KD033 in the Human‐PD‐1/PD‐L1‐Expressing Murine Model Demonstrates PD‐L1 Targeting of IL‐15 in Vivo,” Cancer Immunology, Immunotherapy 72, no. 6 (2023): 1941–1950. 36454338 10.1007/s00262-022-03331-0 PMC10198867

[239] S. A. Martomo , D. Lu , Z. Polonskaya , et al., “Single‐Dose Anti‐PD‐L1/IL‐15 Fusion Protein KD033 Generates Synergistic Antitumor Immunity With Robust Tumor‐Immune Gene Signatures and Memory Responses,” Molecular Cancer Therapeutics 20, no. 2 (2021): 347–356. 33293344 10.1158/1535-7163.MCT-20-0457

[240] Y. Shi , W. Zheng , K. Yang , et al., “Intratumoral Accumulation of Gut Microbiota Facilitates CD47‐Based Immunotherapy via STING Signaling,” Journal of Experimental Medicine 217, no. 5 (2020): e20192282. 32142585 10.1084/jem.20192282 PMC7201921

[241] S. F. Erttmann , P. Swacha , K. M. Aung , et al., “The Gut Microbiota Prime Systemic Antiviral Immunity via the cGAS‐STING‐IFN‐I Axis,” Immunity 55, no. 5 (2022): 847–861.e10. 35545033 10.1016/j.immuni.2022.04.006

[242] J. Gao , X. Zhao , S. Hu , et al., “Gut Microbial DL‐Endopeptidase Alleviates Crohn's Disease via the NOD2 Pathway,” Cell Host & Microbe 30, no. 10 (2022): 1435–1449.e9. 36049483 10.1016/j.chom.2022.08.002

[243] J. Wang , N. Zhu , X. Su , Y. Gao , and R. Yang , “Gut‐Microbiota‐Derived Metabolites Maintain Gut and Systemic Immune Homeostasis,” Cells 12, no. 5 (2023): 793. 36899929 10.3390/cells12050793 PMC10000530

[244] I. Sieminska and J. Baran , “Myeloid‐Derived Suppressor Cells in Colorectal Cancer,” Frontiers in Immunology 11 (2020): 1526. 32849517 10.3389/fimmu.2020.01526 PMC7426395

[245] Y. Che , S. Fu , H. Wang , et al., “Correlation of the Gut Microbiota and Antitumor Immune Responses Induced by a Human Papillomavirus Therapeutic Vaccine,” ACS Infectious Diseases 8, no. 12 (2022): 2494–2504. 36342280 10.1021/acsinfecdis.2c00305

[246] R. C. Simpson , E. R. Shanahan , R. A. Scolyer , and G. V. Long , “Towards Modulating the Gut Microbiota to Enhance the Efficacy of Immune‐Checkpoint Inhibitors,” Nature Reviews Clinical Oncology 20, no. 10 (2023): 697–715. 10.1038/s41571-023-00803-9 37488231

[247] B. Routy , E. Le Chatelier , L. Derosa , et al., “Gut Microbiome Influences Efficacy of PD‐1‐Based Immunotherapy Against Epithelial Tumors,” Science 359, no. 6371 (2018): 91–97. 29097494 10.1126/science.aan3706

[248] Y. Lu , X. Yuan , M. Wang , et al., “Gut Microbiota Influence Immunotherapy Responses: Mechanisms and Therapeutic Strategies,” Journal of Hematology & Oncology 15, no. 1 (2022): 47. 35488243 10.1186/s13045-022-01273-9 PMC9052532

[249] W. Cheng , C. Wu , and J. Yu , “The Role of Gut Microbiota in Cancer Treatment: Friend or Foe?,” Gut 69, no. 10 (2020): 1867–1876. 32759302 10.1136/gutjnl-2020-321153 PMC7497589

[250] B. Routy , J. G. Lenehan , W. H. Miller , et al., “Fecal Microbiota Transplantation Plus Anti‐PD‐1 Immunotherapy in Advanced Melanoma: A Phase I Trial,” Nature Medicine 29, no. 8 (2023): 2121–2132. 10.1038/s41591-023-02453-x 37414899

[251] D. Davar , A. K. Dzutsev , J. A. McCulloch , et al., “Fecal Microbiota Transplant Overcomes Resistance to Anti‐PD‐1 Therapy in Melanoma Patients,” Science 371, no. 6529 (2021): 595–602. 33542131 10.1126/science.abf3363 PMC8097968

[252] E. N. Baruch , I. Youngster , G. Ben‐Betzalel , et al., “Fecal Microbiota Transplant Promotes Response in Immunotherapy‐Refractory Melanoma Patients,” Science 371, no. 6529 (2021): 602–609. 33303685 10.1126/science.abb5920

[253] K. Zarrabi , U. Vaishampayan , and P. Barata , “Enhancing the Immunogenicity of Nivolumab Plus Ipilimumab With Live Bacterial Supplementation in Metastatic Renal Cell Carcinoma,” European Urology Focus 10, no. 6 (2024): 879–881. 39837716 10.1016/j.euf.2025.01.003

[254] R. Barragan‐Carrillo , Z. Zengin , and S. Pal , “Microbiome Modulation for the Treatment of Solid Neoplasms,” Journal of Clinical Oncology 43, no. 24 (2025): 2734–2738. 40644647 10.1200/JCO-25-00374 PMC12352565

[255] M. Lv , M. Chen , R. Zhang , et al., “Manganese Is Critical for Antitumor Immune Responses via cGAS‐STING and Improves the Efficacy of Clinical Immunotherapy,” Cell Research 30, no. 11 (2020): 966–979. 32839553 10.1038/s41422-020-00395-4 PMC7785004

[256] H. Kim , J. Y. Hong , W. Jeon , J. Lee , Y. J. Lee , and I. Ha , “Melittin Regulates Iron Homeostasis and Mediates Macrophage Polarization in Rats With Lumbar Spinal Stenosis,” Biomedicine & Pharmacotherapy 156 (2022): 113776. 36244265 10.1016/j.biopha.2022.113776

[257] P. Handa , S. Thomas , V. Morgan‐Stevenson , et al., “Iron Alters Macrophage Polarization Status and Leads to Steatohepatitis and Fibrogenesis,” Journal of Leukocyte Biology 105, no. 5 (2019): 1015–1026. 30835899 10.1002/JLB.3A0318-108R

[258] F. Voli , E. Valli , L. Lerra , et al., “Intratumoral Copper Modulates PD‐L1 Expression and Influences Tumor Immune Evasion,” Cancer Research 80, no. 19 (2020): 4129–4144. 32816860 10.1158/0008-5472.CAN-20-0471

[259] F. Gao , W. You , L. Zhang , et al., “Copper Chelate Targeting Externalized Phosphatidylserine Inhibits PD‐L1 Expression and Enhances Cancer Immunotherapy,” Journal of the American Chemical Society 147, no. 7 (2025): 5796–5807. 39797790 10.1021/jacs.4c14394

[260] C. Kanellopoulou , A. B. George , E. Masutani , et al., “Mg(2+) Regulation of Kinase Signaling and Immune Function,” Journal of Experimental Medicine 216, no. 8 (2019): 1828–1842. 31196981 10.1084/jem.20181970 PMC6683994

[261] L. Butterfield and Y. Najjar , “Immunotherapy Combination Approaches: Mechanisms, Biomarkers and Clinical Observations,” Nature Reviews Immunology 24, no. 6 (2024): 399–416. 10.1038/s41577-023-00973-8 PMC11460566 38057451

[262] C. Luchini , F. Bibeau , M. Ligtenberg , et al., “ESMO Recommendations on Microsatellite Instability Testing for Immunotherapy in Cancer, and Its Relationship With PD‐1/PD‐L1 Expression and Tumour Mutational Burden: A Systematic Review‐Based Approach,” Annals of Oncology 30, no. 8 (2019): 1232–1243. 31056702 10.1093/annonc/mdz116

[263] A. M. Goodman , S. Kato , L. Bazhenova , et al., “Tumor Mutational Burden as an Independent Predictor of Response to Immunotherapy in Diverse Cancers,” Molecular Cancer Therapeutics 16, no. 11 (2017): 2598–2608. 28835386 10.1158/1535-7163.MCT-17-0386 PMC5670009

[264] J. Shim , H. Kim , H. Cha , et al., “HLA‐Corrected Tumor Mutation Burden and Homologous Recombination Deficiency for the Prediction of Response to PD‐(L)1 Blockade in Advanced Non‐Small‐Cell Lung Cancer Patients,” Annals of Oncology 31, no. 7 (2020): 902–911. 32320754 10.1016/j.annonc.2020.04.004

[265] F. Huber and M. Bassani‐Sternberg , “Defects in Antigen Processing and Presentation: Mechanisms, Immune Evasion and Implications for Cancer Vaccine Development,” Nature Reviews Immunology 26, no. 1 (2026): 23–34. 10.1038/s41577-025-01208-8 40781552

[266] F. Wang , G. Chen , M. Qiu , et al., “Neoadjuvant Treatment of IBI310 Plus Sintilimab in Locally Advanced MSI‐H/dMMR Colon Cancer: A Randomized Phase 1b Study,” Cancer Cell 43, no. 10 (2025): 1958–1967.e2. 41043438 10.1016/j.ccell.2025.09.004

[267] Z. Yu , G. Yang , J. Yue , et al., “Case Report: Successful Treatment of a Case of Lynch Syndrome With Double Primary Ovarian and Rectal Cancer,” Frontiers in Oncology 15 (2025): 1534979. 41127008 10.3389/fonc.2025.1534979 PMC12537393

[268] R. Li , Y. Lin , X. Shen , et al., “Case Report: Failed Response to anti‐PD‐1 Immunotherapy in a Colon Cancer Patient With High Microsatellite Instability,” Frontiers in Oncology 15 (2025): 1636122. 41127013 10.3389/fonc.2025.1636122 PMC12537366

[269] F. Davoudi , A. Moradi , H. Sadeghirad , and A. Kulasinghe , “Tissue Biomarkers of Immune Checkpoint Inhibitor Therapy,” Immunology and Cell Biology 102, no. 3 (2024): 179–193. 38228572 10.1111/imcb.12723

[270] Y. Liu , H. Jiang , Z. Fang , et al., “Progenitor‐Exhausted T Cell as Prognostic Indicator in Esophageal Squamous Cell Carcinoma: Illuminating Their Key Contribution to Tumor Immunity,” Frontiers in Immunology 16 (2025): 1659077. 41080578 10.3389/fimmu.2025.1659077 PMC12510862

[271] M. Mulholland , A. Chalou , S. H. A. Andersson , et al., “Progenitor Exhausted PD‐1(+) T Cells Are Cellular Targets of Immune Checkpoint Inhibition in Atherosclerosis,” Nature Cardiovascular Research 4, no. 10 (2025): 1311–1328. 10.1038/s44161-025-00713-2 PMC12520982 41057609

[272] J. Fumet , C. Latour , L. Nuttin , et al., “Tumor‐Associated Macrophages Produce PGE2 to Promote CD8+ T Cell Exhaustion and Drive Resistance to PD‐L1 Blockade in Microsatellite Stable Colorectal Cancer,” Cancer Research 86, no. 3 (2025): 785–801. 10.1158/0008-5472.CAN-25-0079 41196020

[273] N. Y. Lin , S. Fukuoka , S. Koyama , et al., “Microbiota‐Driven Antitumour Immunity Mediated by Dendritic Cell Migration,” Nature 644, no. 8078 (2025): 1058–1068. 40659786 10.1038/s41586-025-09249-8 PMC12390848

[274] Y. Gao , P. Xu , D. Sun , et al., “Faecalibacterium Prausnitzii Abrogates Intestinal Toxicity and Promotes Tumor Immunity to Increase the Efficacy of Dual CTLA4 and PD‐1 Checkpoint Blockade,” Cancer Research 83, no. 22 (2023): 3710–3725. 37602831 10.1158/0008-5472.CAN-23-0605

[275] A. C. Huang , M. A. Postow , R. J. Orlowski , et al., “T‐Cell Invigoration to Tumour Burden Ratio Associated With anti‐PD‐1 Response,” Nature 545, no. 7652 (2017): 60–65. 28397821 10.1038/nature22079 PMC5554367

[276] V. Anagnostou , C. Ho , G. Nicholas , et al., “ctDNA Response After Pembrolizumab in Non‐Small Cell Lung Cancer: Phase 2 Adaptive Trial Results,” Nature Medicine 29, no. 10 (2023): 2559–2569. 10.1038/s41591-023-02598-9 PMC10579094 37814061

[277] H. Halse , A. J. Colebatch , P. Petrone , et al., “Multiplex Immunohistochemistry Accurately Defines the Immune Context of Metastatic Melanoma,” Scientific Reports 8, no. 1 (2018): 11158. 30042403 10.1038/s41598-018-28944-3 PMC6057961

[278] C. Niveau , M. Cettour‐Cave , S. Mouret , et al., “MCT1 Lactate Transporter Blockade Re‐Invigorates Anti‐Tumor Immunity Through Metabolic Rewiring of Dendritic Cells in Melanoma,” Nature Communications 16, no. 1 (2025): 1083. 10.1038/s41467-025-56392-x PMC11772620 39870647

[279] R. J. Argüello , A. J. Combes , R. Char , et al., “SCENITH: A Flow Cytometry‐Based Method to Functionally Profile Energy Metabolism With Single‐Cell Resolution,” Cell Metabolism 32, no. 6 (2020): 1063–1075.e7. 33264598 10.1016/j.cmet.2020.11.007 PMC8407169

[280] E. A. Chong , C. Alanio , J. Svoboda , et al., “Pembrolizumab for B‐Cell Lymphomas Relapsing After or Refractory to CD19‐Directed CAR T‐Cell Therapy,” Blood 139, no. 7 (2022): 1026–1038. 34496014 10.1182/blood.2021012634 PMC9211527

[281] M. Pan and B. Li , “T Cell Receptor Convergence Is an Indicator of Antigen‐Specific T Cell Response in Cancer Immunotherapies,” Elife 11 (2022): e81952. 36350695 10.7554/eLife.81952 PMC9683788

[282] M. Sacco , et al., “Deep Learning Discriminates Thymic Epithelial Tumors' Histological Subtypes Using Digital Pathology,” Annals of Oncology (2025). 10.1016/j.annonc.2025.12.003 41390119

[283] S. Andani , B. Chen , J. Ficek‐Pascual , et al., “Histopathology‐Based Protein Multiplex Generation Using Deep Learning,” Nature Machine Intelligence 7, no. 8 (2025): 1292–1307. 10.1038/s42256-025-01074-y PMC12364712 40842484

[284] G. De Velasco , Y. Je , D. Bossé , et al., “Comprehensive Meta‐Analysis of Key Immune‐Related Adverse Events From CTLA‐4 and PD‐1/PD‐L1 Inhibitors in Cancer Patients,” Cancer Immunology Research 5, no. 4 (2017): 312–318. 28246107 10.1158/2326-6066.CIR-16-0237 PMC5418853

[285] I. Puzanov , A. Diab , K. Abdallah , et al., “Managing Toxicities Associated With Immune Checkpoint Inhibitors: Consensus Recommendations From the Society for Immunotherapy of Cancer (SITC) Toxicity Management Working Group,” Journal for ImmunoTherapy of Cancer 5, no. 1 (2017): 95. 29162153 10.1186/s40425-017-0300-z PMC5697162

[286] Q. Yin , L. Wu , L. Han , et al., “Immune‐Related Adverse Events of Immune Checkpoint Inhibitors: A Review,” Frontiers in Immunology 14 (2023): 1167975. 37304306 10.3389/fimmu.2023.1167975 PMC10247998

[287] S. Pichon , G. Zebian , C. Bureau , et al., “Life‐Threatening Immune‐Related Adverse Events in the Intensive Care Unit: A Narrative Review,” Intensive Care Medicine 51, no. 12 (2025): 2289–2304. 41123622 10.1007/s00134-025-08155-x

[288] S. Iwama , T. Kobayashi , and H. Arima , “Management, Biomarkers and Prognosis in People Developing Endocrinopathies Associated With Immune Checkpoint Inhibitors,” Nature Reviews Endocrinology 21, no. 5 (2025): 289–300. 10.1038/s41574-024-01077-6 39779950

[289] S. Iwama , A. De Remigis , M. K. Callahan , S. F. Slovin , J. D. Wolchok , and P. Caturegli , “Pituitary Expression of CTLA‐4 Mediates Hypophysitis Secondary to Administration of CTLA‐4 Blocking Antibody,” Science Translational Medicine 6, no. 230 (2014): 230ra45. 10.1126/scitranslmed.3008002 24695685

[290] A. Ribas , R. Dummer , I. Puzanov , et al., “Oncolytic Virotherapy Promotes Intratumoral T Cell Infiltration and Improves Anti‐PD‐1 Immunotherapy,” Cell 170, no. 6 (2017): 1109–1119.e10. 28886381 10.1016/j.cell.2017.08.027 PMC8034392

[291] T. Nopsopon , M. Wu , and D. Sardana , “Analysis of Association of Radiation Therapy With Risk of Adverse Events in Patients Receiving Immunotherapy Using Pooled Trial Data Matched by Propensity Score,” JAMA Oncology 8, no. 7 (2022): 1072. 35511138 10.1001/jamaoncol.2022.0877

[292] G. Zhou , N. Zhang , K. Meng , and F. Pan , “Interaction Between Gut Microbiota and Immune Checkpoint Inhibitor‐Related Colitis,” Frontiers in Immunology 13 (2022): 1001623. 36389768 10.3389/fimmu.2022.1001623 PMC9648670

[293] A. Diab , H. Gogas , S. Sandhu , et al., “Bempegaldesleukin Plus Nivolumab in Untreated Advanced Melanoma: The Open‐Label, Phase III PIVOT IO 001 Trial Results,” Journal of Clinical Oncology 41, no. 30 (2023): 4756–4767. 37651676 10.1200/JCO.23.00172 PMC10602507

[294] J. S. Weber , M. S. Carlino , A. Khattak , et al., “Individualised Neoantigen Therapy mRNA‐4157 (V940) Plus Pembrolizumab Versus Pembrolizumab Monotherapy in Resected Melanoma (KEYNOTE‐942): A Randomised, Phase 2b Study,” Lancet 403, no. 10427 (2024): 632–644. 38246194 10.1016/S0140-6736(23)02268-7

[295] P. Abraham and D. Johnson , “Long‐Term Toxicities of Immune Checkpoint Inhibitors,” Drugs 85, no. 12 (2025): 1535–1549. 41028650 10.1007/s40265-025-02243-4 PMC12615533

[296] J. Wright , A. Powers , and D. Johnson , “Endocrine Toxicities of Immune Checkpoint Inhibitors,” Nature Reviews Endocrinology 17, no. 7 (2021): 389–399. 10.1038/s41574-021-00484-3 PMC8769055 33875857

[297] A. Haugh and A. Daud , “Distinguishing Between Help and Harm: Helper T Cell Subsets and Immune‐Related Adverse Events,” Journal of Clinical Investigation 134, no. 20 (2024): e184310. 39403930 10.1172/JCI184310 PMC11473163

[298] R. Reschke , R. J. Sullivan , E. J. Lipson , A. H. Enk , T. F. Gajewski , and J. C. Hassel , “Targeting Molecular Pathways to Control Immune Checkpoint Inhibitor Toxicities,” Trends in Immunology 46, no. 1 (2025): 61–73. 39732529 10.1016/j.it.2024.11.014 PMC13426271

[299] Y. Hailemichael , D. H. Johnson , N. Abdel‐Wahab , et al., “Interleukin‐6 Blockade Abrogates Immunotherapy Toxicity and Promotes Tumor Immunity,” Cancer Cell 40, no. 5 (2022): 509–523.e6. 35537412 10.1016/j.ccell.2022.04.004 PMC9221568

[300] E. Perez‐Ruiz , L. Minute , I. Otano , et al., “Prophylactic TNF Blockade Uncouples Efficacy and Toxicity in Dual CTLA‐4 and PD‐1 Immunotherapy,” Nature 569, no. 7756 (2019): 428–432. 31043740 10.1038/s41586-019-1162-y

[301] M. J. Walsh , L. R. Ali , P. Lenehan , et al., “Blockade of Innate Inflammatory Cytokines TNFα, IL‐1β, or IL‐6 Overcomes Virotherapy‐Induced Cancer Equilibrium to Promote Tumor Regression,” Immunotherapy Advances 3, no. 1 (2023): ltad011. 37461742 10.1093/immadv/ltad011 PMC10349916

[302] S. Wang and T. Chan , “Navigating Established and Emerging Biomarkers for Immune Checkpoint Inhibitor Therapy,” Cancer Cell 43, no. 4 (2025): 641–664. 40154483 10.1016/j.ccell.2025.03.006 PMC13171157

[303] L. Jiang , C. You , Y. Xiao , et al., “Radiogenomic Analysis Reveals Tumor Heterogeneity of Triple‐Negative Breast Cancer,” Cell Reports Medicine 3, no. 7 (2022): 100694. 35858585 10.1016/j.xcrm.2022.100694 PMC9381418

[304] V. Pietrobon and F. Marincola , “Hypoxia and the Phenomenon of Immune Exclusion,” Journal of Translational Medicine 19, no. 1 (2021): 9. 33407613 10.1186/s12967-020-02667-4 PMC7788724

[305] L. Munn and R. Jain , “Vascular Regulation of Antitumor Immunity,” Science 365, no. 6453 (2019): 544–545. 31395771 10.1126/science.aaw7875 PMC7321824

[306] M. Hutchings , F. Morschhauser , G. Iacoboni , et al., “Glofitamab, a Novel, Bivalent CD20‐Targeting T‐Cell‐Engaging Bispecific Antibody, Induces Durable Complete Remissions in Relapsed or Refractory B‐Cell Lymphoma: A Phase I Trial,” Journal of Clinical Oncology 39, no. 18 (2021): 1959–1970. 33739857 10.1200/JCO.20.03175 PMC8210975

[307] G. Cartron , R. Houot , Y. Al Tabaa , et al., “Glofitamab in Refractory or Relapsed Diffuse Large B Cell Lymphoma After Failing CAR‐T Cell Therapy: A Phase 2 LYSA Study,” Nature Cancer 6, no. 7 (2025): 1173–1183. 40181090 10.1038/s43018-025-00941-2

[308] G. Arena and R. Chiarle , “Mechanisms of Resistance to Novel Immunotherapies in B‐Cell Lymphomas: Focus on CAR T and Bispecific Antibodies,” Cancers (Basel) 17, no. 21 (2025): 3453. 41228246 10.3390/cancers17213453 PMC12609497

[309] H. Kwon and C. Lee , “The Emergence of a New Paradigm With Signal 1 and Signal 2 T‐Cell Engagers in Hematology,” Seminars in Hematology 62, no. 4 (2025): 245–254. 41456996 10.1053/j.seminhematol.2025.11.005

[310] T. Roider , B. J. Brinkmann , V. Kim , et al., “An Autologous Culture Model of Nodal B‐Cell Lymphoma Identifies Ex Vivo Determinants of Response to Bispecific Antibodies,” Blood Advances 5, no. 23 (2021): 5060–5071. 34587238 10.1182/bloodadvances.2021005400 PMC9153026

[311] L. van Hooren , S. M. Handgraaf , D. J. Kloosterman , et al., “CD103(+) Regulatory T Cells Underlie Resistance to Radio‐Immunotherapy and Impair CD8(+) T Cell Activation in Glioblastoma,” Nature Cancer 4, no. 5 (2023): 665–681. 37081259 10.1038/s43018-023-00547-6 PMC10212765

[312] Z. Huang , J. Chen , T. Zhu , et al., “Cancer‐Associated Fibroblasts in the Tumor Microenvironment: Heterogeneity, Crosstalk Mechanisms, and Therapeutic Implications,” Molecular Cancer 25, no. 1 (2025): 19. 41413569 10.1186/s12943-025-02533-1 PMC12853664

[313] B. A. Helmink , S. M. Reddy , J. Gao , et al., “B Cells and Tertiary Lymphoid Structures Promote Immunotherapy Response,” Nature 577, no. 7791 (2020): 549–555. 31942075 10.1038/s41586-019-1922-8 PMC8762581

[314] W. Xu , C. Ma , W. Liu , et al., “Prognostic Value, DNA Variation and Immunologic Features of a Tertiary Lymphoid Structure‐Related Chemokine Signature in Clear Cell Renal Cell Carcinoma,” Cancer Immunology, Immunotherapy 71, no. 8 (2022): 1923–1935. 35043231 10.1007/s00262-021-03123-y PMC10992571

[315] W. Xu , J. Lu , W. Liu , et al., “Heterogeneity in Tertiary Lymphoid Structures Predicts Distinct Prognosis and Immune Microenvironment Characterizations of Clear Cell Renal Cell Carcinoma,” Journal for ImmunoTherapy of Cancer 11, no. 12 (2023): e006667. 38040418 10.1136/jitc-2023-006667 PMC10693897

[316] W. Xu , J. Lu , X. Tian , et al., “Unveiling the Impact of Tertiary Lymphoid Structures on Immunotherapeutic Responses of Clear Cell Renal Cell Carcinoma,” MedComm 5, no. 1 (2024): e461. 38222314 10.1002/mco2.461 PMC10784869

[317] M. B. Hugaboom , L. V. Wirth , K. Street , et al., “Presence of Tertiary Lymphoid Structures and Exhausted Tissue‐Resident T Cells Determines Clinical Response to PD‐1 Blockade in Renal Cell Carcinoma,” Cancer Discovery 15, no. 5 (2025): 948–968. 39992403 10.1158/2159-8290.CD-24-0991 PMC12048281

[318] P. Cakmak , J. H. Lun , A. Singh , et al., “Spatial Immune Profiling Defines a Subset of Human Gliomas With Functional Tertiary Lymphoid Structures,” Immunity 58, no. 11 (2025): 2847–2863.e8. 41125076 10.1016/j.immuni.2025.09.018

[319] G. Kellermann , N. Leulliot , J. Cherfils‐Vicini , M. Blaud , and P. Brest , “Activated B‐Cells Enhance Epitope Spreading to Support Successful Cancer Immunotherapy,” Frontiers in Immunology 15 (2024): 1382236. 38571942 10.3389/fimmu.2024.1382236 PMC10989059

[320] X. Sun , W. Liu , L. Sun , et al., “Maturation and Abundance of Tertiary Lymphoid Structures Are Associated With the Efficacy of Neoadjuvant Chemoimmunotherapy in Resectable Non‐Small Cell Lung Cancer,” Journal for ImmunoTherapy of Cancer 10, no. 11 (2022): e005531. 37011953 10.1136/jitc-2022-005531 PMC9644367

[321] M. Chelvanambi , R. J. Fecek , J. L. Taylor , and W. J. Storkus , “STING Agonist‐Based Treatment Promotes Vascular Normalization and Tertiary Lymphoid Structure Formation in the Therapeutic Melanoma Microenvironment,” Journal for ImmunoTherapy of Cancer 9, no. 2 (2021): e001906. 33526609 10.1136/jitc-2020-001906 PMC7852948

[322] M. Ukita , J. Hamanishi , H. Yoshitomi , et al., “CXCL13‐Producing CD4+ T Cells Accumulate in the Early Phase of Tertiary Lymphoid Structures in Ovarian Cancer,” JCI Insight 7, no. 12 (2022): e157215. 35552285 10.1172/jci.insight.157215 PMC9309049

